# Propylene glycol inactivates respiratory viruses and prevents airborne transmission

**DOI:** 10.15252/emmm.202317932

**Published:** 2023-11-16

**Authors:** Christine T Styles, Jie Zhou, Katie E Flight, Jonathan C Brown, Charlotte Lewis, Xinyu Wang, Michael Vanden Oever, Thomas P Peacock, Ziyin Wang, Rosie Millns, John S O'Neill, Alexander Borodavka, Joe Grove, Wendy S Barclay, John S Tregoning, Rachel S Edgar

**Affiliations:** ^1^ Department of Infectious Disease Imperial College London London UK; ^2^ MRC‐University of Glasgow Centre for Virus Research Glasgow UK; ^3^ Department of Biochemistry University of Cambridge Cambridge UK; ^4^ MRC Laboratory of Molecular Biology Cambridge UK; ^5^ Present address: University College London London UK; ^6^ Present address: Life Edit Therapeutics Morrisville NC USA

**Keywords:** airborne transmission, influenza, propylene glycol, SARS‐CoV‐2, virucide, Evolution & Ecology, Microbiology, Virology & Host Pathogen Interaction

## Abstract

Viruses are vulnerable as they transmit between hosts, and we aimed to exploit this critical window. We found that the ubiquitous, safe, inexpensive and biodegradable small molecule propylene glycol (PG) has robust virucidal activity. Propylene glycol rapidly inactivates a broad range of viruses including influenza A, SARS‐CoV‐2 and rotavirus and reduces disease burden in mice when administered intranasally at concentrations commonly found in nasal sprays. Most critically, vaporised PG efficiently abolishes influenza A virus and SARS‐CoV‐2 infectivity within airborne droplets, potently preventing infection at levels well below those tolerated by mammals. We present PG vapour as a first‐in‐class non‐toxic airborne virucide that can prevent transmission of existing and emergent viral pathogens, with clear and immediate implications for public health.

## Graphical abstract

Existing and emerging viruses pose a great threat to the human population. We show the non‐toxic small molecule propylene glycol (PG) rapidly inactivates airborne and surface virus particles, including SARS‐CoV‐2 and influenza A virus (IAV). PG can therefore limit infection transmission.

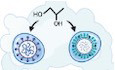

**The paper explained**

**Problem**

Viruses pose a tremendous threat to the worldwide population. Interventions that reduce virus transmission such as lockdowns or social distancing are highly disruptive to society and upgrading ventilation systems is costly. Additionally, there are substantial health and environmental concerns with continuous use of current disinfectants.

**Results**

In this study, we show the widely used, inexpensive and biodegradable non‐toxic molecule propylene glycol prevents infection by many different human viruses, including SARS‐CoV‐2 and influenza A virus. In mouse models where influenza was introduced via the nose, the application of PG along with influenza reduced clinical symptoms of disease and increased survival. Furthermore, safe levels of PG vapour neutralised viruses within airborne and surface droplets.

**Impact**

Propylene glycol vapour could be used to reduce the amount of airborne virus, therefore limiting transmission of respiratory viruses such as influenza and SARS‐CoV‐2, the causative agent of the COVID‐19 pandemic. Additionally, PG is often included as a “non‐active” ingredient in nasal sprays, and this could provide individuals with protection from infection. Although further testing is required outside the laboratory, our data suggest that PG is a valuable weapon in our arsenal to combat the spread of existing and emerging human diseases.

## Introduction

The COVID‐19 pandemic has claimed > 6.6 million lives so far (World Health Organization, 2022a), and the World Health Organization estimates seasonal influenza mortality at 290,000–650,000 people annually (World Health Organization, 2022b). Beyond this health burden, respiratory viruses cause severe economic and societal costs, recently estimated for the UK government alone at £23 billion/year during future influenza‐type pandemics and £8 billion/year for seasonal influenza (National Engineering Policy Centre, 2022). Public health and social measures used to combat respiratory virus transmission include mask‐wearing, physical distancing, lockdown and travel restrictions (Talic *et al*, 2021). Such measures are primarily evidenced by observational studies rather than randomised controlled trials (Glasziou *et al*, 2021; Hirt *et al*, 2022) and require compliance (Fischer *et al*, 2021; Howard *et al*, 2021). Other strategies involve improving ventilation and frequent disinfection to remove infectious virus from the environment, but both come with significant drawbacks, and there is growing concern over the health and environmental consequences of prolonged, widespread use of disinfectants (Curran *et al*, 2019; Rai *et al*, 2020; Burridge *et al*, 2021; Ghafoor *et al*, 2021; Xiao *et al*, 2022). Natural ventilation is not always suitable, increasing risk from air pollutants and vector‐borne diseases along with thermal and energy considerations in colder climates. Expensive engineering solutions like mechanical ventilation require coordinated action across government, health, transport, business, housing and environmental sectors, and a significant culture shift to prioritise infection resilience in indoor environments alongside net zero objectives (National Engineering Policy Centre, 2022). There is an urgent unmet need for novel non‐pharmaceutical interventions to combat emerging and seasonal diseases.

Propylene glycol (PG, propan‐1,2‐diol) is a synthetic liquid compound, whose amphiphilic properties are utilised in a wide range of products and industries: food and drink, cosmetics and pharmaceuticals, including oral, topical, intravenous and nebulised drug delivery (European Medicines Agency, 2014). PG is considered a “Generally Recognised As Safe” (GRAS) molecule, efficiently metabolised and excreted from mammals and is approved for widespread applications by the US Food and Drug Agency (FDA) and European Medicines Agency (EMA) (European Medicines Agency, 2014; Food and Drug Administration (FDA), 2019). PG is largely used as a vehicle or humectant in these preparations due to its water‐absorbing properties; however, it has both anti‐bacterial (Robertson *et al*, 1942; Puck *et al*, 1943; Nalawade *et al*, 2015) and anti‐fungal activity (Faergemann & Fredriksson, 1980; Singh *et al*, 2018). Studies conducted in the 1940s show PG vapour reduced the infectivity of aerosolised bacterial pathogens in mouse models, preventing sepsis‐induced mortality (Robertson *et al*, 1942; Puck *et al*, 1943; Lester *et al*, 1952). In human trials from the same era, vaporised PG reduced the airborne bacterial burden in army barracks (Mather & McClure, 1945), and the introduction of PG vapour on a children's convalescent ward reduced the incidence of undefined respiratory illnesses between 1941 and 1944 (Harris & Strokes, 1945). One study suggested PG vapour could protect mice against disease when exposed to airborne influenza (Robertson *et al*, 1941) and reduce vaccinia‐ and influenza‐mediated fatality in chick embryos (Dunham & MacNeal, 1943; MacNeal & Dunham, 1944). These findings preceded the advent of molecular virology, so the impact of PG on virus particle infectivity was never directly assessed and remains poorly defined.

We tested the hypothesis that PG is virucidal and could reduce respiratory virus transmission by droplet, aerosol and fomite routes.

## Results

### PG inactivates influenza A virus and reduces disease severity

**Figure 1:**
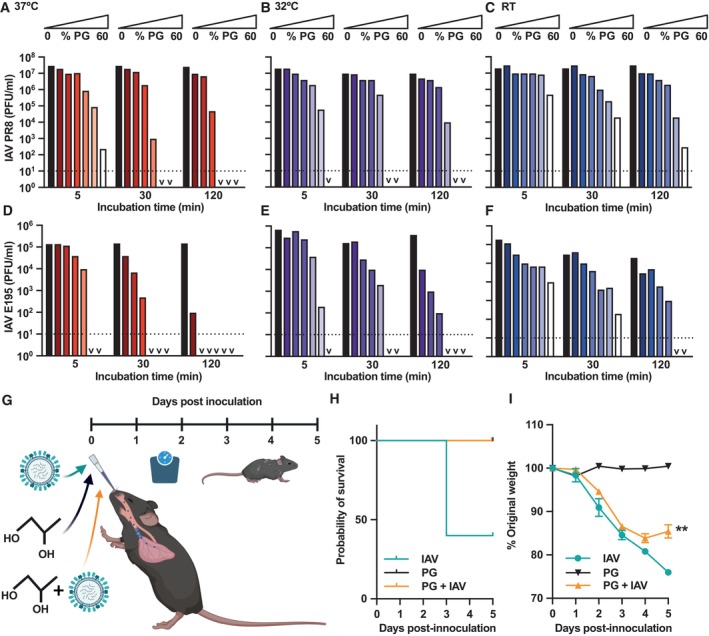
Propylene glycol (PG) reduces influenza A virus infectivity *in vitro* and *in vivo* A–FIAV strains PR8 (A–C) and E195 (D–F) were incubated with 0–60% PG for 5–120 min at (A/D) 37°C, (B/E) 32°C or (C/F) room temperature (RT) and infectivity assessed by plaque assay (*N* = 2); PFU = plaque forming units. 2‐way ANOVA ([PG] × time): (A) [PG] ****P < 0.0001, time ****P < 0.0001, interaction ****P < 0.0001; (B) [PG] ****P < 0.0001, time ****P < 0.0001, interaction ***P < 0.001; (C) [PG] ****P < 0.0001, time ****P < 0.0001, interaction ****P > 0.0001. (D) [PG] ****P < 0.0001, time ****P < 0.0001 interaction ***P < 0.001, (E) [PG] ****P < 0.0001, time ****P < 0.0001, interaction ****P < 0.0001. (F) [PG] ****P < 0.0001, time ****P < 0.0001, interaction ****P < 0.0001. v = < 10^1^; dashed line = limit of detection. Representative replicate shown, see Fig EV1 for repeat.G
*In vivo* methodology. Mice were intranasally inoculated with 50 μl total volume of 20% PG in PBS, 5 × 10^4^ PFU H1N1 Cal09 IAV in PBS, or 20% PG + IAV and monitored for 5 days (*N* = 5 mice/group; mean ± SD).HMouse survival after infection.IWeight loss after infection. Mixed‐effect analysis: [Time] ****P < 0.0001, [PG] ****P < 0.0001; IAV alone versus PG + IAV day 5 **P = 0.0056. For % PG (v/v) conversion to g/l or g/kg see Appendix Table S1. Source data are available online for this figure.

**Figure EV1:**
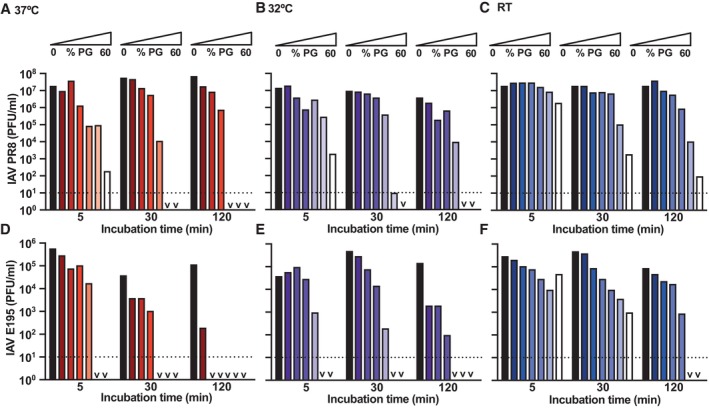
Propylene glycol (PG) inactivates lab strain PR8 IAV and H1N1 2009 pandemic IAV A–FIAV strains PR8 (A–C) and E195 (D–F) were incubated with 0–60% PG for 5–120 min at (A/D) 37°C, (B/E) 32°C or (C/F) room temperature (RT) and infectivity assessed by plaque assay (*N* = 2); PFU = plaque forming units. 2‐way ANOVA ([PG] × time): (A) [PG] ****P < 0.0001, time ****P < 0.0001, interaction ****P < 0.0001; (B) [PG] ****P < 0.0001, time ****P < 0.0001, interaction ***P < 0.001; (C) [PG] ****P < 0.0001, time ****P < 0.0001, interaction ****P > 0.0001. (D) [PG] ****P < 0.0001, time ****P < 0.0001, interaction ***P < 0.001, (E) [PG] ****P < 0.0001, time ****P < 0.0001, interaction ****P < 0.0001. (F) [PG] ****P < 0.0001, time ****P < 0.0001, interaction ****P < 0.0001. v = < 10^1^; dashed line = limit of detection. Representative replicate shown, see Fig 1 for repeat. Source data are available online for this figure.

To examine whether PG exhibits virucidal activity, we first focused on influenza A virus (IAV), a causative agent of seasonal outbreaks in humans and birds that significantly burdens healthcare systems and the poultry farming industry worldwide (World Health Organization, 2022b), and responsible for the worst pandemics of the previous century (Neumann *et al*, 2009). The prototypical IAV lab strain A/PR8/8/34 (henceforth referred to as IAV) was incubated with different concentrations of PG, and infectivity was determined by titration. PG dramatically reduced IAV infection of cultured cells, with virucidal activity dependent on both PG concentration and incubation time (Figs 1A–C and EV1A–C). These results were mirrored with the 2009 influenza pandemic clinical isolate H1N1 A/England/195/09 (E195; Figs 1D–F and EV1D–F). PG‐mediated IAV inactivation was temperature‐dependent, with progressively greater virucidal activity evident at 32°C (nasal and skin temperature) and 37°C (body temperature) compared with 20°C (room temperature, RT). PG had potent virucidal activity at physiological temperatures, reducing IAV infectivity by ~10,000‐fold within 5 min and to undetectable levels after 30 min.

**Figure EV2:**
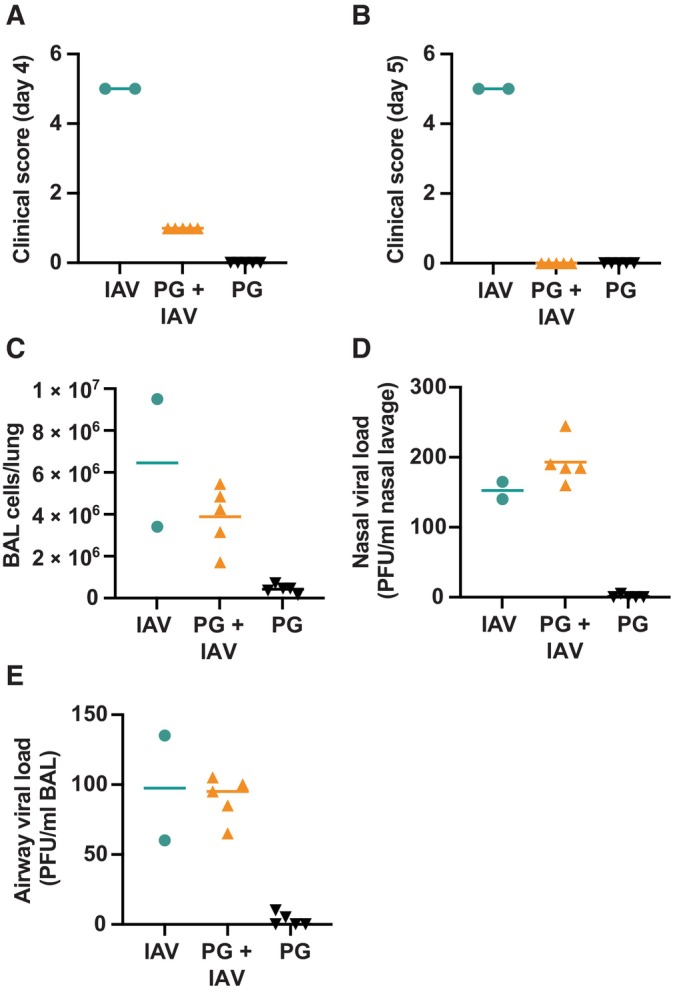
Concomitant inhalation of IAV and PG reduces clinical burden A–EMice were intranasally inoculated with PG alone (20% PG in PBS), H1N1 Cal09 IAV alone (5 × 10^4^ PFU in PBS), or PG + IAV (50 μl total volume for all groups; *N* = 5 mice/group) and monitored for 5 days (see Fig 1D). Clinical scores on day 4 (A) and day 5 (B), BAL cell count (C), viral nasal load (D) and airway viral load (E) on day 5 post‐infection. 3/5 mice in the IAV‐only group were culled prior to day 5 collection point due to poor clinical scores, limiting the power of this study to detect statistically significant differences between groups from day 5 *post‐mortem* immunological and virological assays, but decreased clinical score and inflammatory BAL cell counts were observed in the PG + IAV group compared to IAV alone. Source data are available online for this figure.

To determine the translational potential of PG‐mediated virucidal activity against IAV, we then investigated combined inhalation of the 2009 pandemic strain influenza H1N1 A/California/7/2009 and PG *in vivo*. 20% PG was the lowest concentration to yield statistically significant reduction in IAV infectivity at nasal temperature (Figs 1B and E, and EV1B and E). Therefore, mice were intranasally inoculated with 20% PG alone, IAV alone or IAV and 20% PG in combination, with disease progression tracked over 5 days (Fig 1G). Mice were inoculated immediately after IAV and PG were combined (< 1 min), with negligible PG‐mediated inactivation prior to infection (Fig EV5D). Mice receiving PG alone showed no adverse effects over 5 days, consistent with its long‐established biological safety in mammals. Following inhalation of IAV alongside PG, mice showed enhanced survival and reduced clinical signs compared to mice that inhaled IAV alone (Figs 1H and EV2). 3/5 mice within the IAV group showed such poor clinical scores that they were humanely culled 3 days after infection whereas no mice in the IAV + PG group reached this severity limit, demonstrating the protective nature of PG during infection. Mice also lost significantly less weight when PG was co‐administered with IAV than with IAV alone (Fig 1I). Analysis of the remaining mice on day 5 post‐infection showed PG inhalation reduced clinical score and bronchioalveolar lavage cell counts (Fig EV2A–C), despite equivalent nasal and airway viral loads at this late time post‐infection (Fig EV2D and E). Although further investigations are required to determine whether PG treatment reduces peak inflammatory cell infiltrates and viral loads at earlier times post‐infection, we conclude that PG can safely reduce the infectivity of influenza A virus *in vitro* and *in vivo*.

### PG has broad‐spectrum virucidal activity

**Figure 2:**
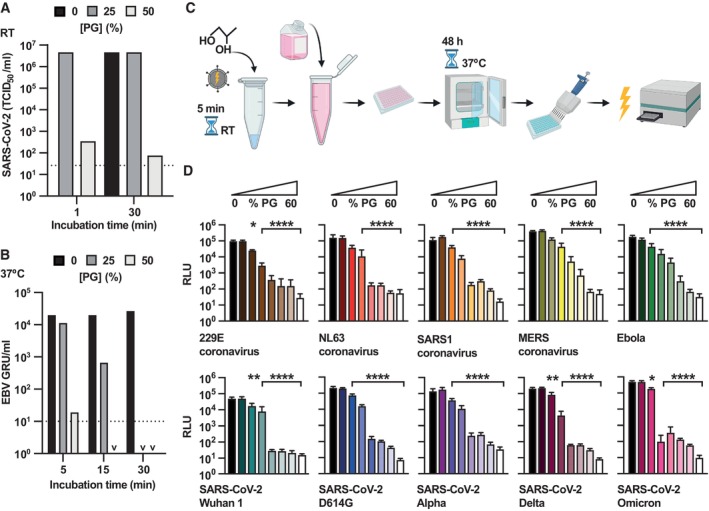
PG inactivates SARS‐CoV‐2, EBV and many different pseudoviruses ASARS‐CoV‐2 incubated with 0–50% [PG] for 1–30 min at RT and infectivity assessed by TCID50 assay (*N* = 2). 2‐way ANOVA ([PG] × time): [PG] ****P < 0.0001, time *P < 0.05, interaction **P < 0.01. Dotted line = limit of detection. Representative replicate shown, see Fig EV3 for repeat.BEBV incubated with 0–50% [PG] for 5–30 min at 37°C and infectivity assessed by titration (*N* = 2; GRU = green Raji units). 2‐way ANOVA ([PG] × time): [PG] ****P < 0.0001, time ****P < 0.0001, interaction ****P < 0.0001; v = < 10^1^. Representative replicate shown, see Fig EV3 for repeat.C, DMethodology (C) and results (D) of lentivirus pseudotypes expressing different glycoproteins incubated with 0–60% PG for 5 min at RT before assessing infectivity by bioluminescence (*N* = 2, *n* = 3; mean ± SD). White bars = mock‐infected. 1‐way ANOVA [PG]: Multiple comparisons **P < 0.01, ***P < 0.001, ****P < 0.0001. Source data are available online for this figure.

**Figure EV3:**
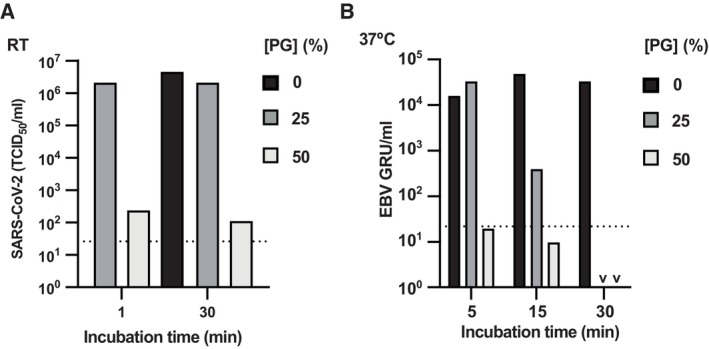
PG inactivates SARS‐CoV‐2 and EBV SARS‐CoV‐2 incubated with 0–50% [PG] for 1–30 min at RT and infectivity assessed by TCID50 assay (*N* = 2, *n* = 4). 2‐way ANOVA ([PG] × time): [PG] ****P < 0.0001, time *P < 0.05, interaction **P < 0.01. Dotted line = limit of detection. Representative replicate shown, see Fig 2 for repeat.EBV incubated with 0–50% [PG] for 5–30 min at 37°C and infectivity assessed by titration (*N* = 2; GRU = green Raji units). 2‐way ANOVA ([PG] × time): [PG] ****P < 0.0001, time ****P < 0.0001, interaction ****P < 0.0001; v = < 10^1^. Representative replicate shown, see Fig 2 for repeat. Source data are available online for this figure.

We next asked whether PG could inactivate other enveloped viruses, including the virus responsible for the COVID‐19 pandemic; severe acute respiratory syndrome coronavirus 2 (SARS‐CoV‐2). We found that PG inactivated the IC19 strain of SARS‐CoV‐2 (McKay *et al*, 2020) with even greater efficiency than observed for IAV (Figs 1C and F, and 2A). After 1 min treatment with 50% PG at room temperature, SARS‐CoV‐2 infectivity decreased by > 10,000‐fold, indicating clear virucidal activity that persisted over longer time frames (Figs 2A and EV3A). PG also efficiently reduced the infectivity of the enveloped double‐stranded DNA gamma‐herpesvirus Epstein Barr (EBV), a lifelong infection carried by most of the human population and associated with numerous cancers (Farrell, 2019). Similar to IAV and SARS‐CoV‐2, PG showed robust virucidal activity against EBV, with > 1,000‐fold reduction in viral titre upon incubation with 50% PG (Figs 2B and EV3B).

To explore the broader context of PG's activity against disease‐causing viruses, we employed a pseudovirus system engineered to express viral envelope glycoproteins from diverse human pathogens, including NL63 and 229E seasonal coronaviruses, severe acute respiratory syndrome coronavirus (SARS‐CoV), middle eastern respiratory syndrome coronavirus (MERS) and Ebola. Using this platform, we also tested PG against glycoproteins from different SARS‐CoV‐2 variants of concern such as omicron. Using a bioluminescence‐based assay for infectivity (Fig 2C), PG significantly limited the infection capability of every different pseudovirus, rapidly reducing entry into susceptible cells in a dose‐dependent manner (Fig 2D; Appendix Fig S1). Although PG consistently reduced infectivity, the concentration required varied between the different pseudovirus‐expressed glycoproteins, recapitulating the variation in the specific potency of PG against IAV, SARS‐CoV‐2 and EBV virus particles. This suggests different PG virucidal thresholds and modes of action against specific viruses, but an overall background of broad‐spectrum virucidal activity.

**Figure 3:**
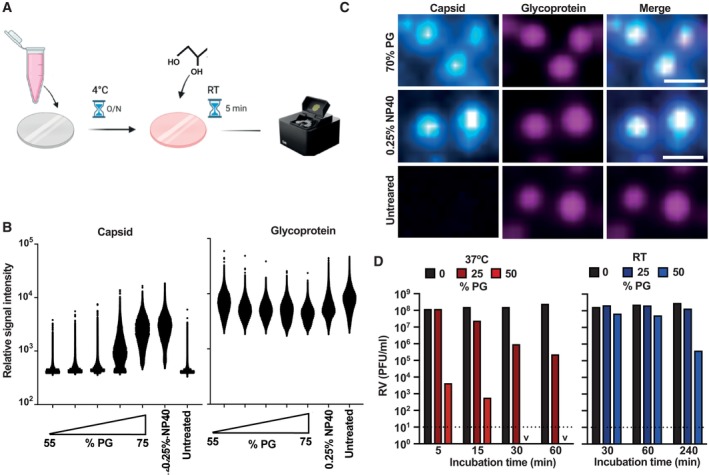
PG mediates viral envelope permeabilisation and inactivates non‐enveloped rotavirus (RV) A, BMethodology (A) and virus particle signal intensities (B) of concentrated SARS‐CoV‐2 pseudovirus immobilised onto coverslips then incubated with 55–75% [PG] for 5 min at RT (*N* = 3). Pseudovirus particles were stained with anti‐capsid and anti‐glycoprotein primary antibodies followed by fluorescently labelled secondary antibodies and then imaged using the Oxford Nanoimager at 100× oil immersion at 488 and 640 nm.CRepresentative enlarged image of immobilised SARS‐CoV‐2 pseudovirus incubated with 70% PG at RT for 5 min, 0.25% NP40 Cell Lysis Buffer at RT for 5 min or PBS control. “Merge” denotes an overlay of both channels. Scale bar = 400 nm.DRotavirus was incubated with 0–50% [PG] for 1–240 min at RT or 37°C and infectivity assessed by titration (*N* = 2); PFU = plaque forming units. 2 way ANOVA ([PG] × time), 37°C: [PG] ****P < 0.0001, time ****P < 0.0001, interaction ****P < 0.0001, RT: [PG] ***P < 0.001, time ****P < 0.0001, interaction ****P < 0.0001. v = < 10^1^; dashed line = limit of detection. Representative replicate shown, see Fig EV4 for repeat. Source data are available online for this figure.

**Figure EV4:**
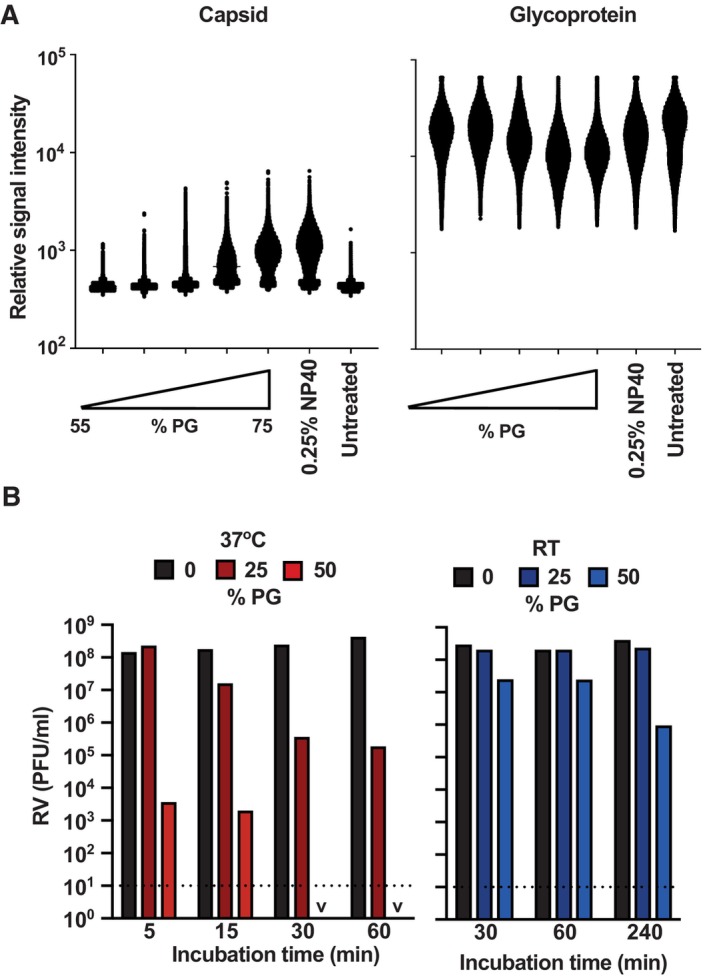
PG mediated viral envelope permeabilisation Virus particle signal intensities of concentrated VSV‐G pseudovirus immobilised onto coverslips then incubated with 55–75% [PG] for 5 min at RT (*N* = 3). Pseudovirus particles were stained with anti‐capsid and anti‐glycoprotein primary antibodies followed by fluorescently labelled secondary antibodies then imaged using the Oxford Nanoimager at 100× oil immersion at 488 and 640 nm.Rotavirus was incubated with 0–50% [PG] for 1–240 min at RT or 37°C and infectivity assessed by titration (*N* = 2); PFU = plaque forming units. 2‐way ANOVA ([PG] × time), 37°C: [PG] ****P < 0.0001, time ****P < 0.0001, interaction ****P < 0.0001, RT: [PG] ***P < 0.001, time ****P < 0.0001, interaction ****P < 0.0001. v = < 10^1^; dashed line = limit of detection. Representative replicate shown, see Fig 3 for repeat. Source data are available online for this figure.

One way that PG could restrict infection is by disrupting the viral phospholipid envelope, as shown for bacterial membranes (Rubiano *et al*, 2020). To assess this, we employed a lentivirus‐based pseudovirus assay, in which concentrated SARS‐CoV‐2 (Wuhan strain) pseudovirus was immobilised onto coverslips and then incubated with PG before staining with anti‐capsid and anti‐glycoprotein antibodies, followed by fluorescently labelled secondary antibodies (Fig 3A). Signal intensity in the capsid and glycoprotein channels was assessed to determine envelope and glycoprotein integrity, with a higher capsid signal indicative of membrane permeabilisation. A significant loss of SARS‐CoV‐2 pseudovirus envelope integrity occurs between 65 and 75% PG treatment, with capsid antibody binding at 75% PG solution equivalent to the NP‐40 detergent control (Fig 3B and C). The same pattern was observed for pseudovirus containing glycoprotein from vesicular stomatitis virus (VSV‐G; Fig EV4A).

SARS‐CoV‐2 Wuhan strain pseudovirus infectivity is restricted at PG concentrations above 20% (v/v) and abolished with 40% PG (Fig 2D), much lower than the PG concentrations required for significant permeabilisation of the viral envelope. Whilst PG‐mediated membrane disruption likely contributes to pseudovirus inactivation, direct effects on viral proteins cannot be discounted, although we observed no consistent difference in glycoprotein signal upon PG treatment with the antibodies used in this study (Figs 3B and C, and EV4A). Therefore, we assessed whether PG treatment could reduce the infectivity of non‐enveloped rotavirus (RV), a causative agent of severe gastroenteritis in children (Crawford *et al*, 2017). PG could exert a virucidal effect against non‐enveloped viruses by altering the structure of surface proteins that mediate host membrane penetration or by direct capsid disruption (Kumar *et al*, 2018). Incubation of the simian RV strain SA11 with PG resulted in time‐, temperature‐ and concentration‐dependent virus inactivation (Figs 3D and EV4B), with similar kinetics to enveloped IAV and EBV (Figs 1A–F, 2B, EV1 and EV3B). This indicates that PG can interfere with viral protein structure to render RV and potentially other non‐enveloped viruses non‐infectious.

### Vaporised PG inactivates airborne viruses

Respiratory droplets and aerosols that are exhaled/expelled by talking, sneezing or coughing from infected individuals represent a major transmission route for many pathogens, particularly respiratory viruses such as influenza and SARS‐CoV‐2 (Leung, 2021). Artificially generated aerosols of SARS‐CoV‐2 and IAV remain infectious for at least 3 h and 1 h, respectively (Kormuth *et al*, 2018; van Doremalen *et al*, 2020), with viable SARS‐CoV‐2 aerosols identified at > 2 m distance from infectious patients (Lednicky *et al*, 2020). The COVID‐19 pandemic has highlighted the clear and pressing need for effective, safe and economical ways to inactivate infectious particles from contaminated air. Current virucidal disinfectants are unsafe for human consumption and often environmentally harmful (Curran *et al*, 2019; Rai *et al*, 2020; Burridge *et al*, 2021; Ghafoor *et al*, 2021; Xiao *et al*, 2022). PG is biodegradable and non‐toxic, with numerous studies showing PG vapour can be safely inhaled for long durations without adverse effects, testing up to 41 mg PG/l air (Robertson & Loosli, 1947; Montharu *et al*, 2010; Werley *et al*, 2011; Fowles *et al*, 2013; Phillips *et al*, 2017; Dalton *et al*, 2018; Langston *et al*, 2021).

**Figure 4:**
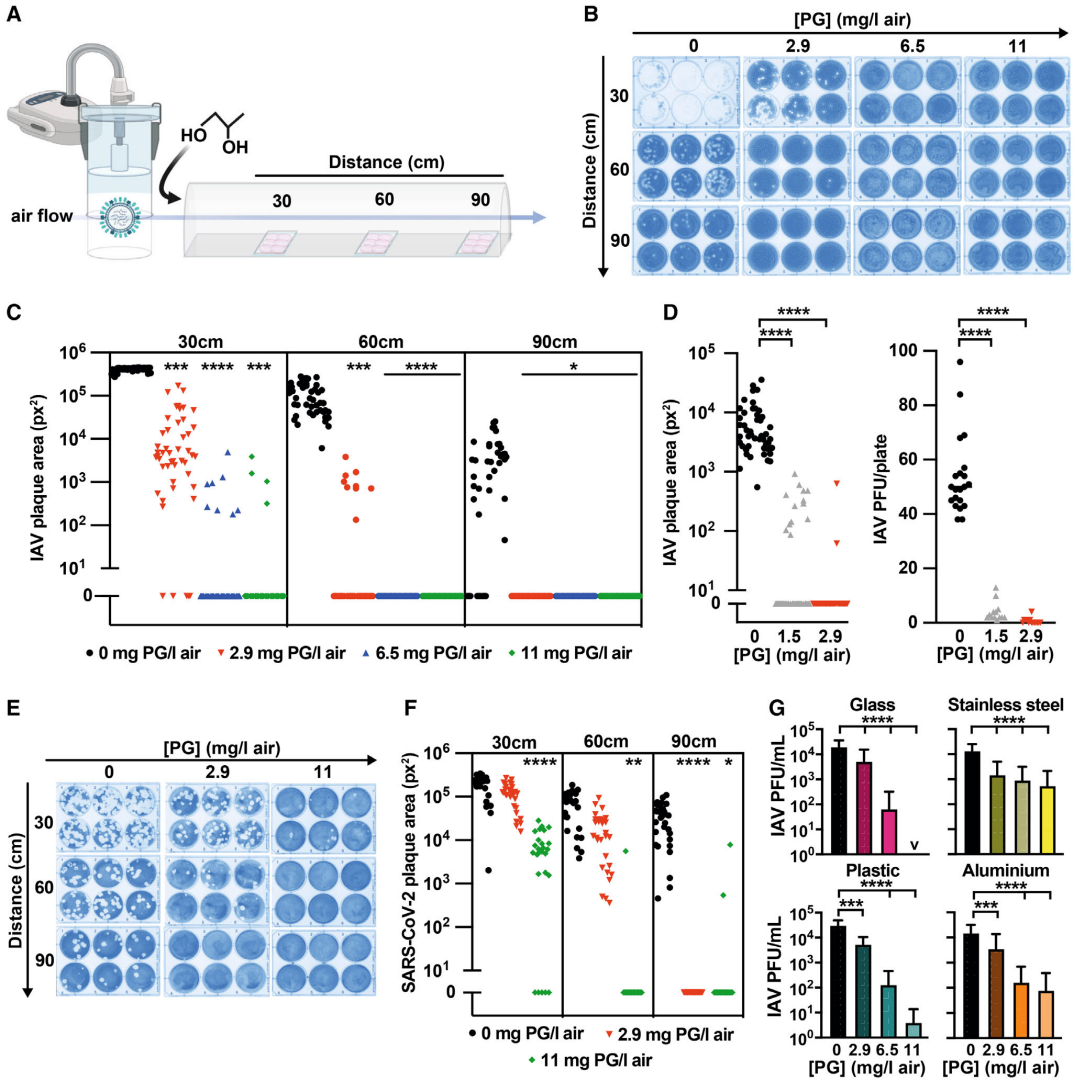
PG vapour efficiently inactivates airborne IAV and SARS‐CoV‐2 AVirus transmission tunnel schematic.B, C(B) PG vapour was introduced into the transmission tunnel to a final concentration of 0–11 mg/l air prior to nebulisation of 10^6^ PFU IAV. Representative IAV culture plates; (C) viral plaque area on culture plates at 30 cm, 60 cm and 90 cm distance was computationally analysed using ImageJ ColonyArea plugin (*N* = 8, *n* = 6); 2‐way ANOVA ([PG] × distance): [PG] ****P < 0.0001, distance ****P < 0.0001, interaction ****P < 0.0001.DPG vapour was introduced into the transmission tunnel to a final PG concentration of 0–2.9 mg/l air prior to nebulisation of 10^4^ PFU IAV. Viral plaque area at 30 cm distance analysed as for (B) (*N* = 8, *n* = 6; 1‐way ANOVA [PG]: ****P < 0.0001) and plaques counted (1‐way ANOVA [PG]: ****P < 0.0001).E, F(E) PG vapour was introduced into the transmission tunnel to a final concentration of 0–11 mg/l air prior to nebulisation of 3 × 10^4^ PFU SARS‐CoV‐2 Delta variant. Representative SARS‐CoV‐2 culture plates; (F) viral plaque area was assessed as per (B) (*N* = 5, *n* = 6); 2‐way ANOVA ([PG] × distance): [PG] ****P < 0.0001, distance ****P < 0.0001, interaction ****P < 0.0001.G10^5^ PFU IAV on fomite model surfaces (plastic, stainless steel, aluminium and glass) were exposed to vaporised PG (0–11 mg/l air) and infectivity assessed by plaque assay after 25 min (*N* ≥ 4, *n* ≥ 3; mean ± SD). 1‐way ANOVA [PG]: *P < 0.05, **P < 0.01, ***P < 0.001, ****P < 0.0001. Source data are available online for this figure.

The condensation of vaporised or aerosolised PG with airborne aqueous respiratory droplets is very energetically favourable and occurs rapidly in atmospheric air at room temperature. We predicted that low levels of vaporised PG would condense with respiratory droplets in sufficient amounts to inactivate any airborne virus particles therein. To model infection by airborne IAV and SARS‐CoV‐2, we used a bespoke transmission tunnel system (Fig 4A) (Singanayagam *et al*, 2020). Within the transmission tunnel, permissive cell monolayers at different distances were exposed to airborne virus droplets (4–6 μm) in the presence of total vaporised PG concentrations from 0 to 11 mg/l air (Appendix Fig S2). Following exposure, viral plaque area was computationally derived via two independent methods. In line with our predictions and the 1941 pathogenesis study (Robertson *et al*, 1941), PG vapour reduced airborne IAV and SARS‐CoV‐2 infectivity in a dose‐dependent manner (Fig 4B–F, Appendix Fig S3), abolishing infection within a distance of < 1 m. Vapour was a more efficacious virucide than PG in solution (Figs 1A–F, EV1, 2 and EV3), as was PG within nebulised droplets (Figs 1A–C vs. EV5), mirroring inhalation in mice (Fig 1G–I).

**Figure EV5:**
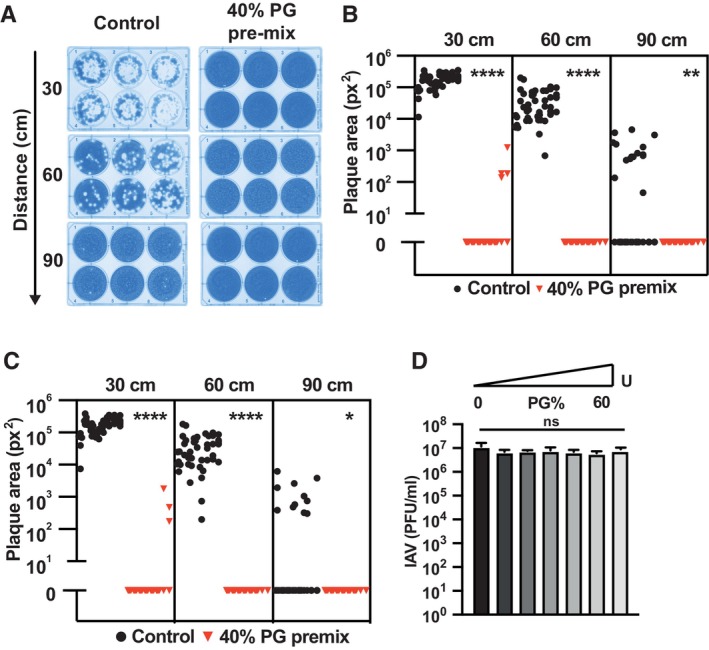
Concomitant nebulisation of PG and IAV prevents infection A–C10^6^ PFU IAV was mixed with PG or PBS control to 40% final concentration and immediately nebulised (< 1 min incubation) into the virus transmission tunnel. (A) Representative plates. Viral plaque area on tissue culture plates at 30 cm, 60 cm and 90 cm were computationally analysed using ImageJ ColonyArea plugin (B) and viral plaque macros (C) (*N* = 8; *n* = 6). 2‐way ANOVA ([PG] × distance): [PG] ****P < 0.0001, distance ****P < 0.0001, interaction ****P < 0.0001.DIAV was mixed with PG to 0–60% final concentration and immediately diluted for plaque assay (*N* = 2; mean ± SD) (< 1 min incubation). 1‐way ANOVA [PG] *P* > 0.05. Source data are available online for this figure.

Whilst the transmission tunnel has some limitations as a model of viral dissemination through droplets and aerosols (see Appendix Fig S3), our findings clearly demonstrate efficient and rapid PG‐mediated inactivation of airborne IAV and SARS‐CoV‐2, at virus levels that far exceed the estimated amounts expelled by speaking, coughing or sneezing (Gustin *et al*, 2013; Lindsley *et al*, 2015; Singanayagam *et al*, 2020). 1.5 mg PG/l air was the lowest exposure we could consistently generate with our experimental system, and it effectively abolished infectivity when less IAV was nebulised into the tunnel to mimic an amount comparable to multiple human coughs (Fig 4D; Appendix Fig S3). Given the strong correlation between initial viral dose, infection probability and disease severity (Chu *et al*, 2004; Memoli *et al*, 2015; Watson *et al*, 2015; Han *et al*, 2019; Spinelli *et al*, 2021; Van Damme *et al*, 2021), clinical studies are now required to identify the optimum aerosolised PG levels that effectively reduce viral transmission under “real‐world” conditions.

Alongside airborne routes, viruses are also transmitted indirectly via contact with surfaces contaminated by the deposition of virus‐containing respiratory droplets and subsequent mechanical transfer to mucous membranes (fomite transmission) (Leung, 2021). Infectious SARS‐CoV‐2 and SARS‐CoV can be recovered up to 72 h after deposition on surfaces, including plastic and stainless steel (van Doremalen *et al*, 2020), and viable H1N1 IAV is recoverable for up to 2 weeks from stainless steel (Thompson & Bennett, 2017). Importantly then, PG vapour inactivated IAV upon varied fomites, with PG at 11 mg/l air sufficient to significantly reduce infectious IAV on all surfaces tested (Fig 4G). As such, we propose PG as the first‐in‐class example of a safe, inexpensive, and environmentally neutral broad‐spectrum virucide, for inactivation of airborne and surface‐bound viruses.

## Discussion

With the increasing threat from emerging pathogens, we need new tools that can immediately be deployed to attenuate viral transmission within social, healthcare and transport settings. For example, sanitising ambulances between patients caused delays and disruption during the COVID‐19 pandemic. PG vapour is a potentially valuable resource for limiting diverse virus infections by multiple routes, including aerosol, droplet and fomite transmission; an economical and effective virucide that is much safer to ingest and inhale compared to other disinfectants and fumigation systems, whilst also avoiding their negative environmental consequences and toxicity (Ghafoor *et al*, 2021). Prospective application of PG as an infection prevention measure requires further investigation beyond laboratory settings, but our results strongly suggest we should leverage its virucidal capacity. PG is already an excipient in non‐prescription nasal sprays so this intervention could be rapidly implemented, and vapour generation utilises existing technologies.

This study demonstrates that PG has broad‐spectrum virucidal activity, against both enveloped and non‐enveloped viruses. Conversely, most conventional antiseptics and disinfectants used within hospitals such as ethanol hand gels exhibit poor activity against non‐enveloped viruses including human norovirus and RVs (World Health Organization, 2009). Although beyond the scope of this study, it is of commercial and pharmaceutical interest to determine whether PG mediates virucidal activity against other non‐enveloped viruses and to further refine its mechanistic action against viral envelopes, glycoproteins and/or capsids. Recently, PG was shown to disrupt rotavirus replication factories inside cells, demonstrating the potential sensitivity of phase‐separated viroplasms to this small molecule, further extending its potential as a broad‐spectrum antiviral with a range of mechanistic approaches (Geiger *et al*, 2021). In addition to virucidal activity, studies from the 1940s suggest PG vapour is bactericidal against many aerosolised bacterial species (Robertson *et al*, 1942; Puck *et al*, 1943; Lester *et al*, 1952). Re‐evaluating these findings using modern experimental methodologies is paramount, given the threat from antimicrobial resistance. As PG appears to act via biophysical disruption of lipid membranes or protein structure, this presents a substantial, if not insurmountable, barrier to evolutionary escape by pathogens.

## Conclusion

Propylene glycol is already approved for use within pharmaceutical, cosmetic and food industries, yet its inherent virucidal activity has not been examined or exploited. PG in nasal sprays, nebulisers and sanitisers could protect vulnerable individuals, whereas direct inactivation of airborne human viruses by PG vapour could potentially reduce the overall infectious burden and transmission rates in clinical and commercial settings.

## Materials and Methods

### Cell culture

All media and supplements were supplied by Gibco‐Life Technologies, and cells were maintained in a humidified incubator at 37°C with 5% CO2. Madin‐Darby Canine Kidney (MDCK; RRID:CVCL_0422), African green monkey kidney (Vero E6; RRID:CVCL_DX71), human hepatoma‐derived 7 cell line (Huh7; RRID:CVCL_B7T1) and human embryonic kidney cells (293T; RRID:CVCL_0063) transduced with an ACE2 lentiviral vector (ACE2‐293T, as described previously; Peacock *et al*, 2021b) were routinely cultured in Dulbecco's modified Eagle's Medium (DMEM). Raji cells (human, RRID:CVCL_0511) were cultured in RPMI‐1640 medium (RPMI). Media was supplemented with 10% foetal calf serum (FCS), GlutaMAX and penicillin/streptomycin. ACE2‐293T cells were additionally supplemented with 1 μg/ml puromycin. Vero E6 cells overexpressing ACE2 and TMPRSS2 (VAT cells, as described previously; Rihn *et al*, 2021), were additionally supplemented with non‐essential amino acids, 0.2 mg/ml Hygromycin B and 2 mg/ml Geneticin™ (G418 Sulfate). Cells were routinely tested for mycoplasma contamination using Mycoplasma PCR Detection Kit (Cambridge Bioscience).

### IAV infectivity plaque assay

Influenza strain A/PR8/8/34 (H1N1) was primarily used in this study and referred to as IAV, and additional influenza strain A/England/195/09 was referred to as E195 and treated in the same way as IAV. IAV was propagated in confluent MDCK cells in the presence of 1 mg/ml TPCK‐treated trypsin (Worthington Bioscience) in serum‐free medium (SFM) (DMEM, penicillin/streptomycin and GlutaMAX). IAV was incubated with 0–60% v/v concentration of propylene glycol (PG) (Sigma) for 5–120 min as described, at either room temperature (RT) or at 32 and 37°C. See Appendix Table S1 for conversion % v/v PG solution to g/l. Following PG treatment, the virus/PG suspensions were serially diluted in SFM with the initial 10^−1^ dilution being the detection limit for IAV infectivity in this assay. Confluent MDCK cells were incubated with each serial dilution for 1 h at 37°C, then input virus was removed by aspiration and cells overlaid with SFM containing 0.14% BSA (Sigma), 0.8% Avicel© (FMC BioPolymer) and 1 mg/ml TPCK‐treated trypsin. After 72 h, cells were fixed in 8–10% formalin/PBS, stained with 0.1% toluidine blue (Sigma) and viral plaque forming units (PFU) assessed.

### IAV fomite infectivity assay with PG vapour

Two microliter droplets containing 10^5^ PFU IAV in SFM were pipetted onto stainless steel discs, polystyrene plastic, aluminium foil sections or glass discs within 6 well plates. Plates were placed in a sealed polystyrene chamber and exposed to vaporised PG (0–11 mg/l air) using a MicroFogger 2 (WorkshopScience). After 25 min, virus was recovered in 1 ml SFM, serially diluted and quantified by plaque assay on MDCK as described above.

### EBV infectivity assay

Prototypical laboratory strain Epstein–Barr Virus (EBV) containing a GFP cassette was incubated with 0–50% v/v PG for 5–120 min at 37°C. Following treatment, virus/PG suspensions were serially diluted in RPMI with the initial 10^−1^ dilution being the detection limit for EBV infectivity in this assay. 5 × 10^4^ Raji cells were added to each dilution and incubated for 48 h at 37°C. RPMI containing 20 nM TPA and 5 mM sodium butyrate was added and a viral titre determined after 24 h using fluorescent microscopy to identify GFP‐expressing cells (Green Raji units; GRU).

### Lentivirus pseudotype infectivity assay

Pseudotype lentiviruses were generated in HEK 293T cells as described previously (Peacock *et al*, 2021b). Briefly, 293T cells were co‐transfected with plasmids encoding desired envelope glycoprotein, firefly luciferase reporter (pCSGW) and pCAGGs‐GAG‐POL using Lipofectamine 3000 (Thermo Fisher) and pseudovirus harvested at 48 h and 72 h post‐transfection. A control pseudovirus was also constructed without a viral glycoprotein component (“bald” pseudovirus; Appendix Fig S1). Pseudoviruses used in this study contained glycoproteins from five different SARS‐CoV‐2 variants (Wuhan‐1 (McKay *et al*, 2020), D614G (Zhou *et al*, 2022), Alpha (Zhou *et al*, 2022), Delta (preprint: Newman *et al*, 2021) and Omicron (preprint: Newman *et al*, 2021)), middle eastern respiratory syndrome coronavirus (MERS‐CoV) (McKay *et al*, 2020), SARS‐CoV (McKay *et al*, 2020), NL63 and 229E coronaviruses (McKay *et al*, 2020), Ebola (Long *et al*, 2015), amphotropic murine leukaemia virus (MLV‐A) (Long *et al*, 2015) or Indiana vesicular stomatitis virus (VSV‐G) (Long *et al*, 2015).

Pseudoviruses were treated with 0–60% v/v [PG] for 5 min at RT and then diluted in growth media. Pseudoviruses were plated in triplicate onto confluent ACE2‐293T cells (SARS‐CoV‐2 variants, SARS‐CoV, NL63, Ebola, MLV‐A, VZV‐G and “bald”) or Huh7 cells (MERS‐CoV and 229E) and incubated for 48 h. Luciferase activity was measured using a Firefly luciferase assay system kit (Promega), on a FLUOstar Omega plate reader (BMG Labtech). Each analysed plate contained triplicate uninfected cells to control for background luminescence.

### SARS‐CoV‐2 TCID50 infectivity assay

The strain of SARS‐CoV‐2 used for infectivity assays was SARS‐CoV‐2/England/IC19 and is henceforth referred to as “SARS‐CoV‐2” (McKay *et al*, 2020). Vero E6 cells in assay diluent (DMEM, 0.3% BSA, NEAA, penicillin/streptomycin) were seeded into 96‐well plates and incubated at 37°C for 24 h. SARS‐CoV‐2 was incubated with 0–50% v/v PG for 1–30 min at RT. Virus/PG suspension was then added to the first column of confluent Vero E6 cells and a log_10_/half‐log_10_ dilution series immediately performed in assay diluent. Technical replicates were performed for each sample. Plates were incubated for 5 days before adding an equal volume of crystal violet stain (0.1% w/v) to live cells. Wells were scored for either an intact, stained cell sheet or the absence of cells due to virus‐induced cytopathic effect. For each condition, the Spearman‐Karber method was used to calculate the 50% tissue culture infectious dose (TCID50) of virus.

### Rotavirus TCID50 infectivity assay

Group A rotavirus (RV, simian strain SA11 G3P[2]) was incubated with 0–50% PG for 1–240 min at RT or 37°C. After PG incubation, RV was serially diluted with serum‐free DMEM (supplemented with 0.5 mg/ml porcine trypsin, penicillin/streptomycin, NEAA, and GlutaMAX) and applied to confluent monolayers of green monkey kidney cells (MA104, RRID:CVCL_3846) seeded in 48‐well plates. Five days after infection, the number of wells with cells showing virus‐induced cytopathic effect (CPE) was recorded. Tissue culture infectious dose 50% (TCID50) was calculated using the Reed and Muench method and converted to plaque‐forming units (PFU) by multiplying a conversion factor of 0.70 PFU/TCID50 (Distefano *et al*, 1995).

### Pseudovirus membrane permeability assay

HEK293T cells were co‐transfected with an HIV packaging construct (pCMV‐dR891), a luciferase reporter plasmid (CSLW) and 200 ng/μl of an expression vector encoding VSV‐G or SARS‐CoV‐2 Wuhan Spike glycoprotein. Supernatants were collected and filtered at 48/72 h post‐transfection and pooled before centrifugation at 86,363 *g* for 2 h at 4°C using a TH‐641 Swinging Bucket Rotor (Thermo Fisher Scientific). The viral pellet was resuspended in 1,000 μl of OptiMEM (Gibco). 25 mm coverslips were sequentially washed in ddH_2_0, ethanol, and methanol for 2 min, then rinsed in acetone and treated with 2% 3‐amino‐propyltriethoxysilane (diluted in acetone) for 5 min, rinsed twice in ddH_2_0, then mounted in Attofluor chambers (Thermo Fisher Scientific). 150 μl of concentrated virus and 150 μl of PBS was added to the coverslips then incubated overnight on a shaker at 4°C. They were then rinsed in PBS and fixed in 4% EM‐grade formaldehyde (Thermo Scientific, diluted in PBS) for 10 min, then rinsed 3× with PBS, treated with PG (diluted in PBS as described) at RT for 5 min and rinsed 10× with PBS. Samples were blocked with 2% BSA/PBS (Thermo Scientific) for 10 min, rinsed 3× with PBS and incubated for 1 h with mouse anti‐SARS‐CoV‐2 spike antibody (clone 1A9; Genetex Inc. [GTX632604], 1 μg/ml) or anti‐VSV‐G glycoprotein antibody (Clone 8G5F11; Kerafast [EB0010], 0.25 μg/ml) diluted in PBS, on a rocker at RT. Following incubation, samples were rinsed twice with PBS, fixed with formaldehyde as described above and permeabilised for 5 min as required using NP40 Cell Lysis Buffer (Invitrogen) diluted 1 in 3 in PBS. Samples were then blocked as described above, rinsed 3× with PBS and incubated for 1 h with anti‐HIV1 p55 + p24 + p17 antibody (Abcam [ab63917], diluted 1:4,000 in PBS) on a rocker at RT. Samples were then rinsed 3× with PBS and incubated with Goat anti‐Mouse IgG Alexa Fluor 647 (Invitrogen [A32728], 5 μg/ml) and Goat anti‐Rabbit IgG Alexa Fluor 488 (Invitrogen [A32731], 5 μg/ml) on a rocker at RT. Samples were rinsed 3× with PBS and post‐fixed. Samples were imaged on an Oxford Nanoimager with 100× oil immersion at 488 nm (Alexa Fluor 488) and 640 nm (Alexa Fluor 647) laser illumination. Images were captured from across several regions of the coverslip, with each image covering a 50 μm × 80 μm area. Images were analysed using FIJI (Schindelin *et al*, 2012). Virus particles were identified using the Find Maxima function, using either the GagPol capsid (488 nm) or glycoprotein (640 nm) channels as reference, according to the experiment. Signal intensities were measured in each channel for every particle. Typically, > 10,000 virus particles were quantified per coverslip sample.

### Virus transmission tunnel experiments with airborne IAV and SARS‐CoV‐2

A custom‐built transmission tunnel system described by Singanayagam *et al* (2020) (Fig 4A) was used to assess aerosolised PG‐mediated inactivation of nebulised IAV and SARS‐CoV‐2 (B.1.617.2, Delta variant; preprint: Peacock *et al*, 2021a) viruses. Briefly, the transmission tunnel holds 3 tissue culture plates at intervals of 30, 60 and 90 cm from the nebuliser (Aerogen Pro nebuliser; Aerogen). A bias flow pump maintains directional airflow from the nebuliser chamber to the exposure tunnel that deposits airborne virus within 4–6 μm diameter droplets across the tissue culture plates during a 10 min exposure period.

All transmission tunnel experiments were performed within a class I (SARS‐CoV‐2) or class II (IAV) biological safety cabinet. MDCK or VAT cells were seeded into 6 well plates for IAV or SARS‐CoV‐2 analysis, respectively. Before placing in the transmission tunnel, MDCK cells were transferred to SFM and VAT cells were transferred to assay medium (MEM, penicillin/streptomycin, 4 mM L‐glutamine, 0.4% (w/v) BSA, 0.32% NaHCO_2_ and 20 mM HEPES). To assess inactivation of IAV and SARS‐CoV‐2 by aerosolised PG, concentrations between 0 and 11 mg/l air were introduced into the exposure tunnel using a MicroFogger 2 (WorkshopScience). To quantify the total airborne PG concentration (vapour plus droplets), sampling was performed using a SKC biosampler attached to the tunnel that collected PG into 1 ml distilled water. Samples were analysed on an Osmomat 3000 (Gonotec) and total PG concentration calculated by comparison to a standard curve of PG concentrations (Appendix Fig S2). After PG was aerosolised within the tunnel, permissive cells were exposed to either 10^4^–10^6^ PFU nebulised IAV in PBS or 3 × 10^4^ nebulised PFU SARS‐CoV‐2 in PBS by starting the directional airflow for 10 min. Cells were then incubated for 1 h at 37°C and 5% CO_2_ in a humidified incubator. For IAV, exposed medium was aspirated and cells overlaid with SFM supplemented with 0.8% v/w Avicel© and before fixation and staining 72 h later as previously described. For SARS‐CoV‐2, assay medium supplemented with Avicel© (0.75% w/v final concentration) was added directly to cells and incubated for 72 h before cells were fixed and stained with crystal violet (0.1% w/v) containing 30% EtOH for > 30 min.

Transmission tunnel tissue culture plates were imaged using a Bio‐Rad Gel Visualiser and each well within the plate was independently computationally analysed. Virus‐mediated clearance of cells (plaque area) was quantified using two independent ImageJ FIJI platform systems (Schindelin *et al*, 2012); the plugin ColonyArea (Guzmán *et al*, 2014) and the macro viral plaque (Cacciabue *et al*, 2019). ColonyArea quantifies the % well area containing stained cells and viral plaque quantifies the % area lacking stained cells due to viral cytopathic effect. Calculations were consistently between the two methods (Fig 4 and Appendix Fig S3; Appendix Fig S3D for correlation). Data are presented as plaque area in square pixels (px^2^). Plaque area shows a strong linear correlation with plaque number at lower viral inputs (*R* ^2^ = 0.72, Spearman's *r* = 0.98, *P* < 0.0001), that becomes non‐linear at higher viral inputs as plaques begin to overlap. To encompass the broadest dynamic range, plaque area is reported for transmission tunnel data.

To assess PG‐mediated inactivation of IAV during aerosolisation (Fig EV5), PBS (0% PG control) or 40% PG was added to 10^6^ PFU and then immediately nebulised into the chamber (< 1 min incubation at RT), MDCK cells exposed to the aerosolised PG/IAV mixture and infectious IAV quantified as above.

### Combined inhalation of PG and IAV *in vivo*

Six to eight‐week‐old female BALB/c mice (RRID:IMSR_JAX:000651) were obtained from Charles River UK Ltd (Portscatho, UK) and kept in specific‐pathogen‐free (SPF) conditions in accordance with United Kingdom's Home Office guidelines. Mice were maintained in autoclaved individually ventilated cages (IVC) under positive pressure. Mice were housed in groups of five animals per cage with *ad libitum* access to food and water. All work was approved by the Animal Welfare and Ethical Review Board (AWERB) at Imperial College London.

For infections, adult mice were anaesthetised using isoflurane and inoculated intranasally (i.n.) with 50 μl final volume of either 5 × 10^4^ PFU H1N1 A/California/7/2009 influenza virus (IAV), 5 × 10^4^ IAV and 20% PG, or 20% PG alone in sterile PBS (see Appendix Table S1 for conversion of % v/v PG solution to g/kg mouse weight). Mice were inoculated immediately after IAV and PG were mixed. Mice were weighed after infection. Mice were scored for signs of clinical illness after infection based on an adapted scoring system (Graham *et al*, 1991). Scores were given (0–5, with 0 being in healthy condition) for coat, activity, stance and breathing. Investigators were not blinded to treatment. Mice were culled using 100 μl intraperitoneal pentobarbitone (20 mg dose, Pentoject, Animalcare Ltd. UK), and tissues collected and cells in bronchoalveolar lavage (BAL) were counted as previously described (Groves *et al*, 2018). Nasal lavage and BAL were processed for viral load by plaque assay on MDCK cells as described above.

### Virus biological safety levels

All experiments with IAV, EBV, RV and pseudoviruses were conducted in class 2 microbiology safety cabinets in Biological Safety Level 2 laboratories. All experiments with SARS‐CoV‐2 were conducted in a class 1 microbiology safety cabinet in a Biological Safety Level 3 laboratory.

### Statistical analysis

Data were analysed in GraphPad Prism and presented as mean ± standard deviation (SD) or representative replicate data as noted. At least 2 independent biological replicates were performed for each experimental condition, of which details can be found in respective figure legends and summarised in Appendix Table S2. Statistical tests for virus assays were performed on log_10_‐transformed data (*Y* = log_10_[*Y* + 1]). Detailed information for one‐way and two‐way ANOVA analyses including F values, degrees of freedom and replicate details is shown in Appendix Table S2. Statistical test outcomes including multiple comparisons are summarised in Appendix Table S3. Investigators were not blinded to treatment during *in vivo* experiments.

### Graphics

Synopsis, Figs 1G, 2C, 3A and 4A graphics were created with BioRender.com.

## Author contributions

**Christine T Styles:** Data curation; formal analysis; supervision; investigation; visualization; methodology; writing – original draft; writing – review and editing. **Jie Zhou:** Investigation; methodology; writing – review and editing. **Katie E Flight:** Investigation; methodology; writing – review and editing. **Jonathan C Brown:** Formal analysis; investigation; methodology; writing – review and editing. **Charlotte Lewis:** Formal analysis; investigation; visualization; methodology; writing – review and editing. **Xinyu Wang:** Formal analysis; investigation; methodology; writing – review and editing. **Michael Vanden Oever:** Investigation; methodology; writing – review and editing. **Thomas P Peacock:** Investigation; methodology; writing – review and editing. **Ziyin Wang:** Investigation; writing – review and editing. **Rosie Millns:** Investigation; writing – review and editing. **John S O'Neill:** Supervision; funding acquisition; project administration; writing – review and editing. **Alexander Borodavka:** Supervision; funding acquisition; methodology; project administration; writing – review and editing. **Joe Grove:** Supervision; funding acquisition; methodology; project administration; writing – review and editing. **Wendy S Barclay:** Supervision; funding acquisition; methodology; project administration; writing – review and editing. **John S Tregoning:** Formal analysis; supervision; methodology; project administration; writing – review and editing. **Rachel S Edgar:** Conceptualization; data curation; formal analysis; supervision; funding acquisition; visualization; methodology; writing – original draft; project administration; writing – review and editing.

## Disclosure and competing interests statement

The authors declare that they have no conflict of interest.

## For more information

https://www.imperial.ac.uk/people/rachel.edgar.

## Supporting information



Appendix

Expanded View Figures PDF

Source Data for Expanded View

PDF+

Source Data for Figure 1

Source Data for Figure 2

Source Data for Figure 3

Source Data for Figure 4

## Data Availability

This study includes no data deposited in external repositories.
