# Author Correction: Propylene glycol inactivates respiratory viruses and prevents airborne transmission

**DOI:** 10.1038/s44321-024-00078-2

**Published:** 2024-06-06

**Authors:** Christine T Styles, Jie Zhou, Katie E Flight, Jonathan C Brown, Charlotte Lewis, Xinyu Wang, Michael Vanden Oever, Thomas P Peacock, Ziyin Wang, Rosie Millns, John S O’Neill, Alexander Borodavka, Joe Grove, Wendy S Barclay, John S Tregoning, Rachel S Edgar

**Affiliations:** 1 Department of Infectious Disease, Imperial College London, London, UK; 2 MRC-University of Glasgow Centre for Virus Research, Glasgow, UK; 3 Department of Biochemistry, University of Cambridge, Cambridge, UK; 4 MRC Laboratory of Molecular Biology, Cambridge, UK; 5 Present Address: University College London, London, UK; 6 Present Address: Life Edit Therapeutics, Morrisville, NC 27560 USA

## Abstract

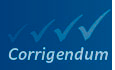

**Correction to:** *EMBO Molecular Medicine* (2023) 15:e17932. 10.15252/emmm.202317932 | Published online 16 November 2023

The Abstract is updated.

A citation is added.

In the Abstract, the sentence—

“We present PG vapour as a first-in-class non-toxic airborne virucide that can prevent transmission of existing and emergent viral pathogens, with clear and immediate implications for public health”.

Is updated to: Changes are in bold.

“We present PG vapour as a **novel** non-toxic airborne virucide that can prevent transmission of existing and emergent viral pathogens, with clear and immediate implications for public health”.

The citation is added:

Desai G, Ramachandran G, Goldman E, Esposito W, Galione A, Lal A, Choueiri TK, Fay A, Jordan W, Schaffner DW, Caravanos J, Grignard E, Mainelis G (2023). Efficacy of Grignard Pure to inactivate airborne phage MS2, a common SARS-CoV-2 surrogate. Environ Sci Technol 57(10):4231–4240

Author Statement:

The authors note that they missed a citation to Desai et al., 2023, which reported the inactivation of bacteriophage MS2 by another agent, triethylene glycol. Although Desai et al did not test for virucidal activity against clinically important human pathogens, in vivo efficacy or potential toxicity, for the avoidance of doubt the statement “first-in-class non-toxic airborne virucide” in the Abstract was thus corrected to “novel non-toxic airborne virucide”.

