# Supplementary material for: Propylene glycol inactivates respiratory viruses and prevents airborne transmission
Source: EMBO Mol Med. 2023 Nov 16;15(12):e17932. doi: 10.15252/emmm.202317932 (PMC10701621; doi:10.15252/emmm.202317932)
Supplement: Supplementary file 1 — Appendix [file EMMM-15-e17932-s008.pdf]

Appendix

Propylene glycol inactivates respiratory viruses and prevents airborne transmission

Authors: Christine T. Styles[1], Jie Zhou[1], Katie E. Flight[1†], Jonathan C. Brown[1], Charlotte Lewis[2], Xinyu Wang[3], Michael Vanden Oever[1,††], Thomas P. Peacock[1], Ziyin Wang[1], Rosie Millns[1], John S. O'Neill[4], Alexander Borodavka[3], Joe Grove[2], Wendy S. Barclay[1], John S. Tregoning[1] and Rachel S. Edgar[1*]

*Correspondence: rachel.edgar@imperial.ac.uk

This PDF file includes:

Appendix Figure S1: Page 2
Appendix Figure S2: Page 3
Appendix Figure S3: Page 4
Appendix Table S1: Page 5
Appendix Table S2: Page 6
Appendix Table S3: Page 9

Appendix Figure S1. PG exhibits virucidal activity against pseudoviruses expressing viral glycoproteins.

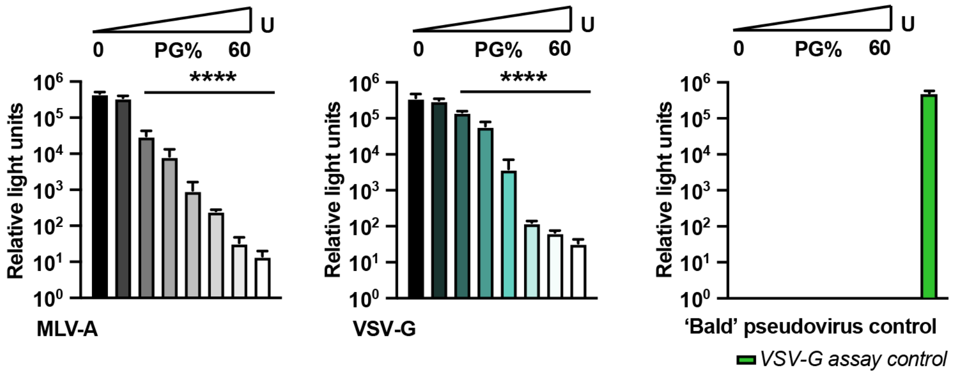

Lentivirus pseudotypes containing different viral glycoproteins from MLV-A (amphotropic murine leukaemia virus) and VSV-G (vesicular stomatis virus), and a 'bald' pseudovirus without viral glycoprotein were incubated with 0-60% [PG] for 5min at RT. Virus infectivity was assessed by firefly luciferase luminescence (N=2, n=3; mean±SD). White bars show background luminescence from mock infected cells. 1-way ANOVA [PG] ****P<0.0001.

Appendix Figure S2. Quantification of PG vapor within the virus transmission tunnel.

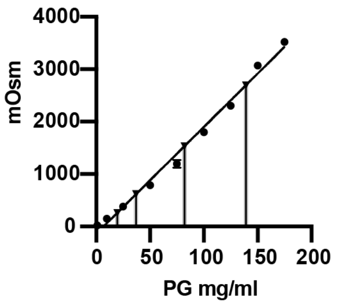

MicroFogger duration (s)	Av. total [PG] (droplet + vapor) collected in SKC sampler	Av. total [PG] in virus transmission tunnel (mg/L air)
5	19.57 ± 2.77	1.54 ± 0.28
10	37.21 ± 2.20	2.93 ± 0.17
20	82.04 ± 9.35	6.46 ± 0.74
30	139.09 ± 12.52	10.96 ± 0.99

PG vapor was introduced into the transmission tunnel using a MicroFogger 2 device activated for different durations (0, 5, 10, 20 and 30s)(N=3 for each duration). Air flow was switched on and total airborne PG within the tunnel (droplets plus vapor) collected in a connected SKC sampler. Osmolarity of the PG samples was assessed (N=3, n=3; mean±SD) and their concentration determined by comparison to the osmolarity of different [PG] standards.

Appendix Figure S3. Virus transmission tunnel: Computational determination of IAV and SARS-CoV-2 plaque area is consistent between ImageJ ColonyArea and ViralPlaque analyses.

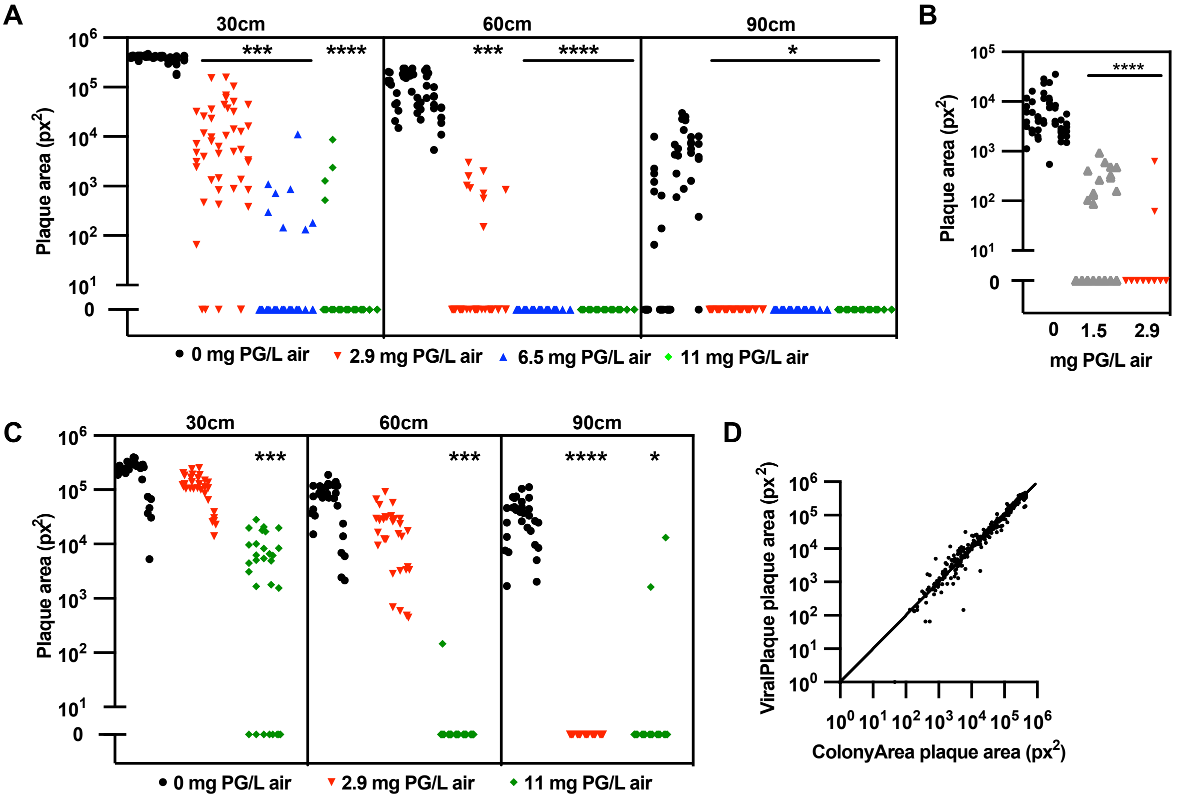

As an *in vitro* model of viral transmission, the IVT has caveats to be considered: nebulised virus droplets are uniform compared to the heterogenous nature of aerosols and respiratory droplets and they also lack respiratory secretions and accompanying salts, glycoproteins and lipids found *in vivo.* (**A)** PG vapor was introduced into the virus transmission tunnel to a final concentration of 0-11 mg/L air prior to nebulization of 10^6 PFU IAV. Viral plaque area on tissue culture plates at 30, 60 and 90cm from nebulizer was computationally analysed using ImageJ ViralPlaque plugin (N=8, n=6). 2-way ANOVA ([PG] x distance): [PG] ****P<0.0001, distance ****P<0.0001, interaction ****P<0.0001. (**B**). PG vapor was introduced into the virus transmission tunnel to a final concentration of 0-2.9 mg/L air prior to nebulization of 10^4 PFU IAV. Viral plaque area on plates at 30cm distance from nebulizer was computationally analysed as per (**A**) (N=8, n=6); 1-way ANOVA [PG] ****P<0.0001. (**C**) PG vapor was introduced into the virus transmission tunnel to a final concentration of 0-11 mg PG air prior to nebulization of $3x10^4$ PFU SARS-CoV-2. Viral plaque area was assessed as per (**A**)(N=5, n=6). Differential spacial distribution of IAV and SARS-CoV-2 particles on cell culture plates may reflect specific PG inactivation thresholds or differential infection efficiency of respective permissive cells. 2-way ANOVA [treatment x distance]: treatment ***P<0.001, distance ****P<0.0001, interaction ****P<0.0001. (**D**) Comparison of ColonyArea and ViralPlaque analyses (R^2= 0.9648).

Appendix Table S1: Conversion of PG solution from % v/v to g/L or g/kg mouse weight.

PG solution conversion from % (v/v) to g/L

**% PG**	RT density (g/L)	**g/L of PG at RT**	40°C density (g/L)	**g/L of PG at 40°C**
**0**	0.998	**0**	0.993	**0**
**10**	1.006	**100.6**	0.998	**99.8**
**20**	1.014	**202.8**	1.004	**200.8**
**30**	1.022	**306.6**	1.01	**303**
**40**	1.03	**412**	1.016	**406.4**
**50**	1.038	**519**	1.023	**511.5**
**60**	1.046	**627.6**	1.029	**617.4**

Intranasal PG dose per mouse (g/kg)

- Specific gravity 20% PG solution: 1.017 g/L
- Volume: 0.00005 L
- Total solution mass: 1.017*0.00005 = 0.00005085 g
- PG mass: 0.00005085/5 = 0.00001017 g
- **PG dose for average 30g mouse:** 0.00001017*(1000/30) = **0.000339 g/kg**

Appendix Table S2. Degrees of freedom, replicates and F value for data analysed by ANOVA statistical methods. N=biological replicates, n=technical replicates. ANOVA multiple comparisons: Dunnett (1-way) & Tukey (2-way).

Figure	ANOVA Type	Figure section		Degree of Freedom	F value (DFn, DFd)	Replicates
1A/EV1A	2-way ANOVA (Dunnett's)	IAV RT	Interaction	12	F (12, 21) = 27.09	N=2
			Time	2	F (2, 21) = 108.8	
			PG level	6	F (6, 21) = 369.7	
1B/EV1B	2-way ANOVA (Dunnett's)	IAV 32	Interaction	12	F (12, 21) = 5.572	N=2
			Time	2	F (2, 21) = 26.63	
			PG level	6	F (6, 21) = 113.5	
1C/EV1C	2-way ANOVA (Dunnett's)	IAV 37	Interaction	12	F (12, 21) = 13.17	N=2
			Time	2	F (2, 21) = 61.50	
			PG level	6	F (6, 21) = 109.7	
1D/EV1D	2-way ANOVA (Dunnett's)	IAV 37	Interaction	12	F (12, 21) = 16.32	N=2
			Time	2	F (2, 21) = 135.1	
			PG level	6	F (6, 21) = 113.7	
1E/EV1E	2-way ANOVA (Dunnett's)	IAV 32	Interaction	12	F (12, 21) = 3.959	N=2
			Time	2	F (2, 21) = 30.80	
			PG level	6	F (6, 21) = 84.95	
1F/EV1F	2-way ANOVA (Dunnett's)	IAV RT	Interaction	12	F (12, 21) = 16.47	N=2
			Time	2	F (2, 21) = 153.9	
			PG level	6	F (6, 21) = 100.2	
2A/EV3A	2-way ANOVA (Tukey's)	SARS-CoV-2	Interaction	4	F (4, 6) = 0.9989	N=2, n=4
			Time	2	F (1.0, 3.0) = 1.002	
			PG level	2	F (2, 3) = 26.72	
2B/EV3B	2-way ANOVA (Tukey's)	EBV	Interaction	8	F (4, 9) = 35.13	N=2
			Time	4	F (2, 9) = 62.90	
			PG level	2	F (2, 9) = 281.5	
2D	1-way ANOVA (Dunnett's)	Ebola- Pseudovirus	Treatment (between columns)	6	F (6, 35) = 219.7	N=2, n=3
			Residual (within columns)	35		
		NL63 Coronavirus- Pseudovirus	Treatment (between columns)	6	F (6, 35) = 43.52	N=2, n=3
			Residual (within columns)	35		
		SARS-CoV-1- Pseudovirus	Treatment (between columns)	6	F (6, 35) = 671.6	N=2, n=3
			Residual (within columns)	35		
		SARS-CoV-2 Wuhan 1- Pseudovirus	Treatment (between columns)	6	F (6, 35) = 375.2	N=2, n=3
			Residual (within columns)	35		
		SARS-CoV-2 D614G- Pseudovirus	Treatment (between columns)	6	F (6, 35) = 3528	N=2, n=3
			Residual (within columns)	35		
		SARS-CoV-2 Alpha- Pseudovirus	Treatment (between columns)	6	F (6, 35) = 458.0	N=2, n=3
			Residual (within columns)	35		
		VZV- Pseudovirus	Treatment (between columns)	6	F (7, 40) = 100.1	N=2, n=3
			Residual (within columns)	35		
		Ampho- Pseudovirus	Treatment (between columns)	6	F (7, 40) = 381.7	N=2, n=3
			Residual (within columns)	35		

		SARS-CoV-2 Delta	Treatment (between columns)	6	F (6, 35) = 538.9	
		Pseudovirus	Residual (within columns)	35		N=2, n=3
		SARS-CoV-2 Omicron	Treatment (between columns)	6	F (6, 35) = 288.9	
		Pseudovirus	Residual (within columns)	35		N=2, n=3
		MERS	Treatment (between columns)	6	F (6, 35) = 113.4	
		Pseudovirus	Residual (within columns)	35		N=2, n=3
		229E	Treatment (between columns)	6	F (6, 35) = 104.7	
		Pseudovirus	Residual (within columns)	F (6, 35) = 458.0		N=2, n=3
		RV 37	Interaction	6	F (6, 12) = 82.27	
	2-way ANOVA (Dunnett's)		Time	3	F (3, 12) = 228.6	
			PG level	2	F (2, 12) = 3362	N=2
		RV RT	Interaction	4	F (4, 9) = 24.25	
	2-way ANOVA (Dunnett's)		Time	2	F (2, 9) = 19.16	
**3D/EV4B**			PG level	2	F (2, 9) = 117.0	N=2
		Plaque Area	Interaction	6	F (6, 56) = 8.559	
	2-way ANOVA (Tukey's)		PG level	3	F (3, 28) = 108.3	
**4C**			Distance	2	F (1.923, 53.85) = 71.08	N=8, n=6
	1-way ANOVA (Dunnett's)	Plaque Area	Treatment (between columns)	2	F (2, 15) = 43.34	
			Residual (within columns)	15		N=8, n=6
	1-way ANOVA (Dunnett's)	Plaque Count	Treatment (between columns)	2	F (2, 45) = 157.3	
**4D**			Residual (within columns)	45		N=8, n=6
		Plaque Area	Interaction	4	F (4, 24) = 18.00	
	2-way ANOVA (Tukey's)		PG level	2	F (2, 12) = 67.87	
**4F**			Distance	2	F (1.286, 15.43) = 47.09	N=5, n=6
		IAV-Plastic	Treatment (between columns)	3	F (3, 59) = 102.8	
			Residual (within columns)	59		N=4-6, n=3
		IAV-Metal (SS)	Treatment (between columns)	3	F (3, 44) = 16.92	
			Residual (within columns)	44		N=4-6, n=3
		IAV-Metal (A)	Treatment (between columns)	3	F (3, 49) = 18.82	
			Residual (within columns)	49		N=4-6, n=3
	1-way ANOVA (Dunnett's)	IAV-Glass	Treatment (between columns)	3	F (3, 53) = 39.28	
**4G**			Residual (within columns)	53		N=4-6, n=3
		Plaque Area	Interaction	2	F (2, 28) = 12.21	
**EV5B Colony Area**	2-way ANOVA (Tukey's)		PG level	1	F (1, 14) = 374.5	
			Distance	2	F (1.602, 22.43) = 21.82	N=8, n=6
		Plaque Area	Interaction	2	F (2, 28) = 16.41	
**EV5B ViralPlaque**	2-way ANOVA (Tukey's)		PG level	1	F (1, 14) = 188.0	
			Distance	2	F (1.530, 21.42) = 28.60	N=8, n=6
		IAV	Interaction	6	F (6, 7) = 1.045	
	2-way ANOVA (Dunnett's)					
**EV5C**			PG level	7		N=2
**Appendix**				**Degree of Freedom**	**F (DFn, DFd)**	**Replicates**
	1-way ANOVA (Dunnett's)	MLV-A-	Treatment (between columns)	7	F (7, 40) = 381.7	
**1**		Pseudovirus	Residual (within columns)	40		N=2, n=3

		VSV-G -	Treatment (between columns)	7	F (7, 40) = 100.1	
		Pseudovirus	Residual (within columns)	40		N=2, n=3
		Bald (no glycoproteins)	Treatment (between columns)	8	F (8, 45) = 272.4	
		Pseudovirus	Residual (within columns)	45		N=2, n=3
**3A**	2-way ANOVA (Tukey's)	Plaque Area	Interaction	6	F (3, 28) = 118.1	N=8, n=6
			PG level	3	F (1.903, 53.28) = 67.24	
			Distance	2	F (28, 56) = 1.042	
**3B**	1-way ANOVA (Dunnett's)	Plaque Area	Treatment (between columns)	2	F (2, 15) = 43.34	N=8, n=6
			Residual (within columns)	15		
**3C**	2-way ANOVA (Tukey's)	Plaque Area	Interaction	4	F (2, 12) = 67.87	N=5, n=6
			PG level	2	F (1.286, 15.43) = 47.09	
			Distance	2	F (12, 24) = 0.7451	

Appendix Table S3. Summary of multiple comparison statistical significance.

Figure				Significance	P value
1A/EV1A	IAV 37	5	Control vs. 10% PG	ns	0.967
			Control vs. 20% PG	ns	0.9998
			Control vs. 30% PG	ns	0.1714
			Control vs. 40% PG	***	0.0002
			Control vs. 50% PG	****	<0.0001
			Control vs. 60% PG	****	<0.0001
		30	Control vs. 10% PG	ns	0.9986
			Control vs. 20% PG	ns	0.6133
			Control vs. 30% PG	*	0.0352
			Control vs. 40% PG	****	<0.0001
			Control vs. 50% PG	****	<0.0001
			Control vs. 60% PG	****	<0.0001
		120	Control vs. 10% PG	ns	0.5706
			Control vs. 20% PG	ns	0.2177
			Control vs. 30% PG	****	<0.0001
			Control vs. 40% PG	****	<0.0001
			Control vs. 50% PG	****	<0.0001
			Control vs. 60% PG	****	<0.0001
1B/EV1B	IAV 32	5	Control vs. 10% PG	ns	>0.9999
			Control vs. 20% PG	ns	0.9459
			Control vs. 30% PG	ns	0.4041
			Control vs. 40% PG	ns	0.5468
			Control vs. 50% PG	*	0.0105
			Control vs. 60% PG	****	<0.0001
		30	Control vs. 10% PG	ns	>0.9999
			Control vs. 20% PG	ns	0.9939
			Control vs. 30% PG	ns	0.9642
			Control vs. 40% PG	ns	0.1487
			Control vs. 50% PG	****	<0.0001
			Control vs. 60% PG	****	<0.0001
		120	Control vs. 10% PG	ns	0.9906
			Control vs. 20% PG	ns	0.5468
			Control vs. 30% PG	ns	0.6134
			Control vs. 40% PG	***	0.0007
			Control vs. 50% PG	****	<0.0001
			Control vs. 60% PG	****	<0.0001
1C/EV1C	IAV RT	5	Control vs. 10% PG	ns	0.9756
			Control vs. 20% PG	ns	>0.9999
			Control vs. 30% PG	ns	>0.9999

			Control vs. 40% PG	ns	0.9687
			Control vs. 50% PG	ns	0.6634
			Control vs. 60% PG	**	0.0011
		30	Control vs. 10% PG	ns	0.9993
			Control vs. 20% PG	ns	0.6322
			Control vs. 30% PG	ns	0.5351
			Control vs. 40% PG	*	0.0301
			Control vs. 50% PG	****	<0.0001
			Control vs. 60% PG	****	<0.0001
		120	Control vs. 10% PG	ns	0.9993
			Control vs. 20% PG	ns	0.5926
			Control vs. 30% PG	ns	0.1075
			Control vs. 40% PG	**	0.0015
			Control vs. 50% PG	****	<0.0001
			Control vs. 60% PG	****	<0.0001
1D/EV1D	IAV 37	5	Control vs. 10% PG	ns	0.9985
			Control vs. 20% PG	ns	0.7564
			Control vs. 30% PG	ns	0.4897
			Control vs. 40% PG	*	0.0248
			Control vs. 50% PG	****	<0.0001
			Control vs. 60% PG	****	<0.0001
		30	Control vs. 10% PG	ns	0.2969
			Control vs. 20% PG	ns	0.0575
			Control vs. 30% PG	***	0.0006
			Control vs. 40% PG	****	<0.0001
			Control vs. 50% PG	****	<0.0001
			Control vs. 60% PG	****	<0.0001
		120	Control vs. 10% PG	****	<0.0001
			Control vs. 20% PG	****	<0.0001
			Control vs. 30% PG	****	<0.0001
			Control vs. 40% PG	****	<0.0001
			Control vs. 50% PG	****	<0.0001
			Control vs. 60% PG	****	<0.0001
1E/EV1E	IAV 32	5	Control vs. 10% PG	ns	>0.9999
			Control vs. 20% PG	ns	0.9995
			Control vs. 30% PG	ns	0.9902
			Control vs. 40% PG	ns	0.0897
			Control vs. 50% PG	****	<0.0001
			Control vs. 60% PG	****	<0.0001
		30	Control vs. 10% PG	ns	>0.9999
			Control vs. 20% PG	ns	0.5781
			Control vs. 30% PG	ns	0.1051

			Control vs. 40% PG	***	0.0007
			Control vs. 50% PG	****	<0.0001
			Control vs. 60% PG	****	<0.0001
		120	Control vs. 10% PG	*	0.0282
			Control vs. 20% PG	**	0.0039
			Control vs. 30% PG	****	<0.0001
			Control vs. 40% PG	****	<0.0001
			Control vs. 50% PG	****	<0.0001
			Control vs. 60% PG	****	<0.0001
1F/EV1F	IAV RT	5	Control vs. 10% PG	ns	0.9809
			Control vs. 20% PG	ns	0.2232
			Control vs. 30% PG	*	0.0313
			Control vs. 40% PG	**	0.0037
			Control vs. 50% PG	***	0.0006
			Control vs. 60% PG	***	0.0003
		30	Control vs. 10% PG	ns	>0.9999
			Control vs. 20% PG	ns	0.233
			Control vs. 30% PG	*	0.013
			Control vs. 40% PG	****	<0.0001
			Control vs. 50% PG	****	<0.0001
			Control vs. 60% PG	****	<0.0001
		120	Control vs. 10% PG	ns	0.339
			Control vs. 20% PG	ns	0.2639
			Control vs. 30% PG	**	0.0082
			Control vs. 40% PG	****	<0.0001
			Control vs. 50% PG	****	<0.0001
			Control vs. 60% PG	****	<0.0001
1H	IAV mouse		Flu Only vs. PG + Flu	**	0.0056
2A/EV3A	SARS-CoV-2	1	Control vs. 25% PG	ns	0.6902
			Control vs. 50% PG	****	<0.0001
		30	Control vs. 25% PG	ns	0.6902
			Control vs. 50% PG	****	<0.0001
2B/EV3B	EBV	5	Control vs. 25% PG	ns	0.901
			Control vs. 50% PG	****	<0.0001
		15	Control vs. 25% PG	***	0.0001
			Control vs. 50% PG	****	<0.0001
		30	Control vs. 25% PG	****	<0.0001
			Control vs. 50% PG	****	<0.0001
2D	229E- coronavirus pseudovirus		0% PG vs. 10% PG	ns	>0.9999
			0% PG vs. 20% PG	*	0.0214
			0% PG vs. 30% PG	****	<0.0001
			0% PG vs. 40% PG	****	<0.0001

			0% PG vs. 50% PG	****	<0.0001
			0% PG vs. 60% PG	****	<0.0001
	NL63-coronavirus pseudovirus		0% PG vs. 10% PG	ns	>0.9999
			0% PG vs. 20% PG	ns	0.2424
			0% PG vs. 30% PG	****	<0.0001
			0% PG vs. 40% PG	****	<0.0001
			0% PG vs. 50% PG	****	<0.0001
			0% PG vs. 60% PG	****	<0.0001
	SAR1 coronavirus		0% PG vs. 10% PG	ns	0.0547
			0% PG vs. 20% PG	****	<0.0001
			0% PG vs. 30% PG	****	<0.0001
			0% PG vs. 40% PG	****	<0.0001
			0% PG vs. 50% PG	****	<0.0001
			0% PG vs. 60% PG	****	<0.0001
	MERS coronavirus		0% PG vs. 10% PG	ns	>0.9999
			0% PG vs. 20% PG	ns	0.065
			0% PG vs. 30% PG	****	<0.0001
			0% PG vs. 40% PG	****	<0.0001
			0% PG vs. 50% PG	****	<0.0001
			0% PG vs. 60% PG	****	<0.0001
	Ebola pseudovirus		0% PG vs. 10% PG	ns	0.5366
			0% PG vs. 20% PG	****	<0.0001
			0% PG vs. 30% PG	****	<0.0001
			0% PG vs. 40% PG	****	<0.0001
			0% PG vs. 50% PG	****	<0.0001
			0% PG vs. 60% PG	****	<0.0001
	SARS-CoV-2 Wuhan 1		0% PG vs. 10% PG	ns	>0.9999
			0% PG vs. 20% PG	**	0.0013
			0% PG vs. 30% PG	****	<0.0001
			0% PG vs. 40% PG	****	<0.0001
			0% PG vs. 50% PG	****	<0.0001
			0% PG vs. 60% PG	****	<0.0001
	SARS-CoV-2 D614G		0% PG vs. 10% PG	ns	0.8286
			0% PG vs. 20% PG	****	<0.0001
			0% PG vs. 30% PG	****	<0.0001
			0% PG vs. 40% PG	****	<0.0001
			0% PG vs. 50% PG	****	<0.0001
			0% PG vs. 60% PG	****	<0.0001
	SARS-CoV-2 Alpha		0% PG vs. 10% PG	ns	0.6474
			0% PG vs. 20% PG	****	<0.0001
			0% PG vs. 30% PG	****	<0.0001
			0% PG vs. 40% PG	****	<0.0001

			0% PG vs. 50% PG	****	<0.0001
			0% PG vs. 60% PG	****	<0.0001
	SARS-CoV-2 Delta		0% PG vs. 10% PG	ns	0.9991
			0% PG vs. 20% PG	**	0.0031
			0% PG vs. 30% PG	****	<0.0001
			0% PG vs. 40% PG	****	<0.0001
			0% PG vs. 50% PG	****	<0.0001
			0% PG vs. 60% PG	****	<0.0001
	SARS-CoV-2 Omicron		0% PG vs. 10% PG	ns	>0.9999
			0% PG vs. 20% PG	*	0.0445
			0% PG vs. 30% PG	****	<0.0001
			0% PG vs. 40% PG	****	<0.0001
			0% PG vs. 50% PG	****	<0.0001
			0% PG vs. 60% PG	****	<0.0001
3D/EV4B	RT 37	5	0% PG vs. 25% PG	ns	0.8242
			0% PG vs. 50% PG	****	<0.0001
		15	0% PG vs. 25% PG	***	0.0003
			0% PG vs. 50% PG	****	<0.0001
		30	0% PG vs. 25% PG	****	<0.0001
			0% PG vs. 50% PG	****	<0.0001
		60	0% PG vs. 25% PG	****	<0.0001
			0% PG vs. 50% PG	****	<0.0001
	RV RT	30	0% PG vs. 25% PG	ns	0.9851
			0% PG vs. 50% PG	**	0.0067
		60	0% PG vs. 25% PG	ns	0.9882
			0% PG vs. 50% PG	**	0.0045
		120	0% PG vs. 25% PG	ns	0.2909
			0% PG vs. 50% PG	****	<0.0001
4C		Plate 1	0 vs. 2.93 PG mg/L air	***	0.0004
			0 vs. 6.46 PG mg/L air	****	<0.0001
			0 vs. 10.593 PG mg/L air	***	0.0001
		Plate 2	0 vs. 2.93 PG mg/L air	***	0.0007
			0 vs. 6.46 PG mg/L air	****	<0.0001
			0 vs. 10.593 PG mg/L air	****	<0.0001
		Plate 3	0 vs. 2.93 PG mg/L air	*	0.0131
			0 vs. 6.46 PG mg/L air	*	0.0131
			0 vs. 10.593 PG mg/L air	*	0.0131
4D	Plaque area	Plate 1	0 vs. 1.541 PG mg/L air	****	<0.0001
			0 vs. 2.93 PG mg/L air	****	<0.0001
	Plaque count	Plate 1	0 vs. 1.541 PG mg/L air	****	<0.0001
			0 vs. 2.93 PG mg/L air	****	<0.0001
4F		Plate 1	0 vs. 2.93 PG mg/L air	ns	0.4524

			0 vs. 10.593 PG mg/L air	****	<0.0001
		Plate 2	0 vs. 2.93 PG mg/L air	ns	0.1804
			0 vs. 10.593 PG mg/L air	**	0.0051
		Plate 3	0 vs. 2.93 PG mg/L air	****	<0.0001
			0 vs. 10.593 PG mg/L air	*	0.0183
4G		Plastic	Control vs. 10	***	0.0002
			Control vs. 20	****	<0.0001
			Control vs. 30	****	<0.0001
		Stainless steel	Control vs. 10	****	<0.0001
			Control vs. 20	****	<0.0001
			Control vs. 30	****	<0.0001
		Glass	Control vs. 10	****	<0.0001
			Control vs. 20	****	<0.0001
			Control vs. 30	****	<0.0001
		Aluminium	Control vs. 10	***	0.0007
			Control vs. 20	****	<0.0001
			Control vs. 30	****	<0.0001
EV5B	ColonyArea	Plate 1	0% vs 40%	****	<0.0001
		Plate 2	0% vs 40%	****	<0.0001
		Plate 3	0% vs 40%	**	0.003
	ViralPlaque	Plate 1	0% vs 40%	****	<0.0001
		Plate 2	0% vs 40%	****	<0.0001
		Plate 3	0% vs 40%	*	0.0338
EV5C	IAV		0% PG vs. 10% PG	ns	0.3435
			0% PG vs. 20% PG	ns	0.4395
			0% PG vs. 30% PG	ns	0.5515
			0% PG vs. 40% PG	ns	0.3435
			0% PG vs. 50% PG	ns	0.2029
			0% PG vs. 60% PG	ns	0.5515
Appendix 1	MLV-A pseudovirus		0% PG vs. 10% PG	****	<0.0001
			0% PG vs. 20% PG	****	<0.0001
			0% PG vs. 30% PG	****	<0.0001
			0% PG vs. 40% PG	****	<0.0001
			0% PG vs. 50% PG	****	<0.0001
			0% PG vs. 60% PG	****	<0.0001
	VSV-G pseudovirus		0% PG vs. 10% PG	ns	0.0559
			0% PG vs. 20% PG	****	<0.0001
			0% PG vs. 30% PG	****	<0.0001
			0% PG vs. 40% PG	****	<0.0001
			0% PG vs. 50% PG	****	<0.0001
			0% PG vs. 60% PG	****	<0.0001
	Bald' pseudovirus control		0% PG vs. 10% PG	ns	>0.9999

			0% PG vs. 20% PG	ns	>0.9999
			0% PG vs. 30% PG	ns	>0.9999
			0% PG vs. 40% PG	ns	>0.9999
			0% PG vs. 50% PG	ns	>0.9999
			0% PG vs. 60% PG	ns	>0.9999
Appendix 3A		Plate 1	0 vs. 2.93 PG mg/L air	***	0.0003
			0 vs. 6.46 PG mg/L air	***	0.0001
			0 vs. 10.593 PG mg/L air	****	<0.0001
		Plate 2	0 vs. 2.93 PG mg/L air	***	0.0008
			0 vs. 6.46 PG mg/L air	****	<0.0001
			0 vs. 10.593 PG mg/L air	****	<0.0001
		Plate 3	0 vs. 2.93 PG mg/L air	*	0.0134
			0 vs. 6.46 PG mg/L air	*	0.0134
			0 vs. 10.593 PG mg/L air	*	0.0134
Appendix 3B	Plaque area	Plate 1	0 vs. 1.541 PG mg/L air	****	<0.0001
			0 vs. 2.93 PG mg/L air	****	<0.0001
Appendix 3C		Plate 1	0 vs. 2.93 PG mg/L air	ns	0.4524
			0 vs. 10.593 PG mg/L air	****	<0.0001
		Plate 2	0 vs. 2.93 PG mg/L air	ns	0.1804
			0 vs. 10.593 PG mg/L air	**	0.0051
		Plate 3	0 vs. 2.93 PG mg/L air	****	<0.0001
			0 vs. 10.593 PG mg/L air	*	0.0183
