# Supplementary material for: Propylene glycol inactivates respiratory viruses and prevents airborne transmission
Source: EMBO Mol Med. 2023 Nov 16;15(12):e17932. doi: 10.15252/emmm.202317932 (PMC10701621; doi:10.15252/emmm.202317932)
Supplement: Supplementary file 2 — Expanded View Figures PDF [file EMMM-15-e17932-s004.pdf]

# Expanded View Figures

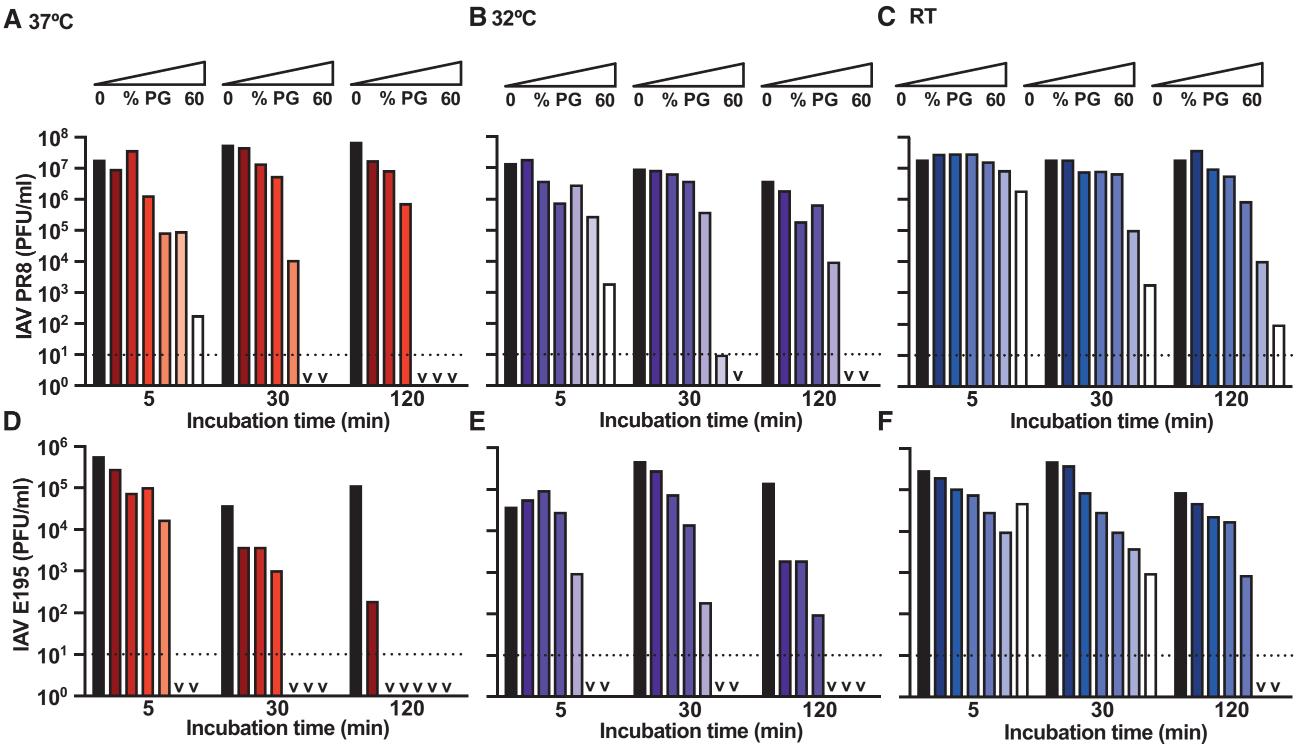


**Figure EV1. Propylene glycol (PG) inactivates lab strain PR8 IAV and H1N1 2009 pandemic IAV.**

A–F IAV strains PR8 (A–C) and E195 (D–F) were incubated with 0–60% PG for 5–120 min at (A/D) 37°C, (B/E) 32°C or (C/F) room temperature (RT) and infectivity assessed by plaque assay ($N = 2$); PFU = plaque forming units. 2-way ANOVA ([PG] × time): (A) [PG] ****$P < 0.0001$, time ****$P < 0.0001$, interaction ****$P < 0.0001$; (B) [PG] ****$P < 0.0001$, time ****$P < 0.0001$, interaction ***$P < 0.001$; (C) [PG] ****$P < 0.0001$, time ****$P < 0.0001$, interaction ****$P > 0.0001$. (D) [PG] ****$P < 0.0001$, time ****$P < 0.0001$, interaction ***$P < 0.001$, (E) [PG] ****$P < 0.0001$, time ****$P < 0.0001$, interaction ****$P < 0.0001$. (F) [PG] ****$P < 0.0001$, time ****$P < 0.0001$, interaction ****$P < 0.0001$. v = $< 10^1$; dashed line = limit of detection. Representative replicate shown, see Fig 1 for repeat.

Source data are available online for this figure.

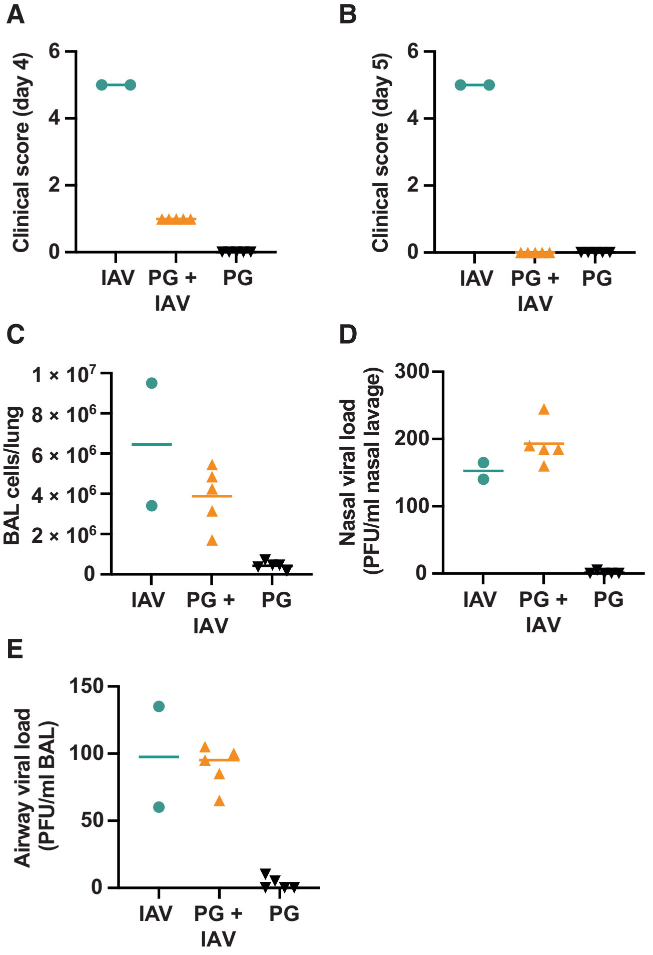


**Figure EV2. Concomitant inhalation of IAV and PG reduces clinical burden.**

A–E Mice were intranasally inoculated with PG alone (20% PG in PBS), H1N1 Cal09 IAV alone ($5 \times 10^4$ PFU in PBS), or PG + IAV (50 μl total volume for all groups; *N* = 5 mice/group) and monitored for 5 days (see Fig 1D). Clinical scores on day 4 (A) and day 5 (B), BAL cell count (C), viral nasal load (D) and airway viral load (E) on day 5 post-infection. 3/5 mice in the IAV-only group were culled prior to day 5 collection point due to poor clinical scores, limiting the power of this study to detect statistically significant differences between groups from day 5 *post-mortem* immunological and virological assays, but decreased clinical score and inflammatory BAL cell counts were observed in the PG + IAV group compared to IAV alone.

Source data are available online for this figure.

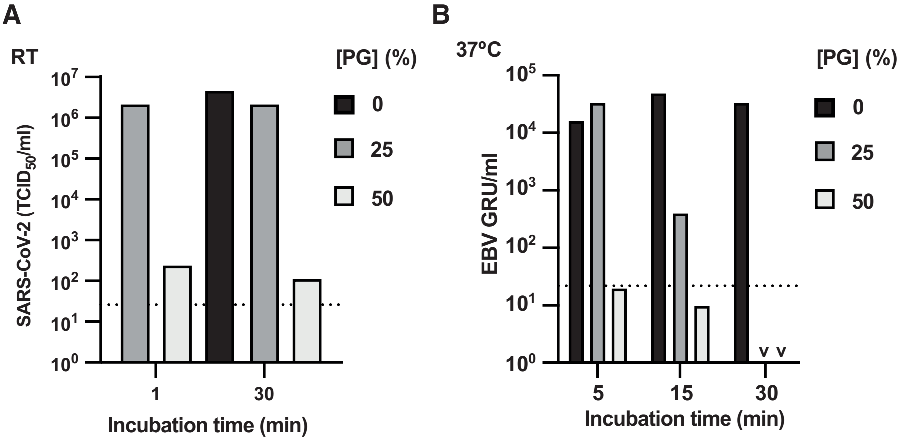


**Figure EV3. PG inactivates SARS-CoV-2 and EBV.**

A SARS-CoV-2 incubated with 0–50% [PG] for 1–30 min at RT and infectivity assessed by TCID50 assay (*N* = 2, *n* = 4). 2-way ANOVA ([PG] × time): [PG] *****P* < 0.0001, time **P* < 0.05, interaction ***P* < 0.01. Dotted line = limit of detection. Representative replicate shown, see Fig 2 for repeat.

B EBV incubated with 0–50% [PG] for 5–30 min at 37°C and infectivity assessed by titration (*N* = 2; GRU = green Raji units). 2-way ANOVA ([PG] × time): [PG] *****P* < 0.0001, time *****P* < 0.0001, interaction *****P* < 0.0001; v = < $10^1$. Representative replicate shown, see Fig 2 for repeat.

Source data are available online for this figure.


© 2023 The Authors

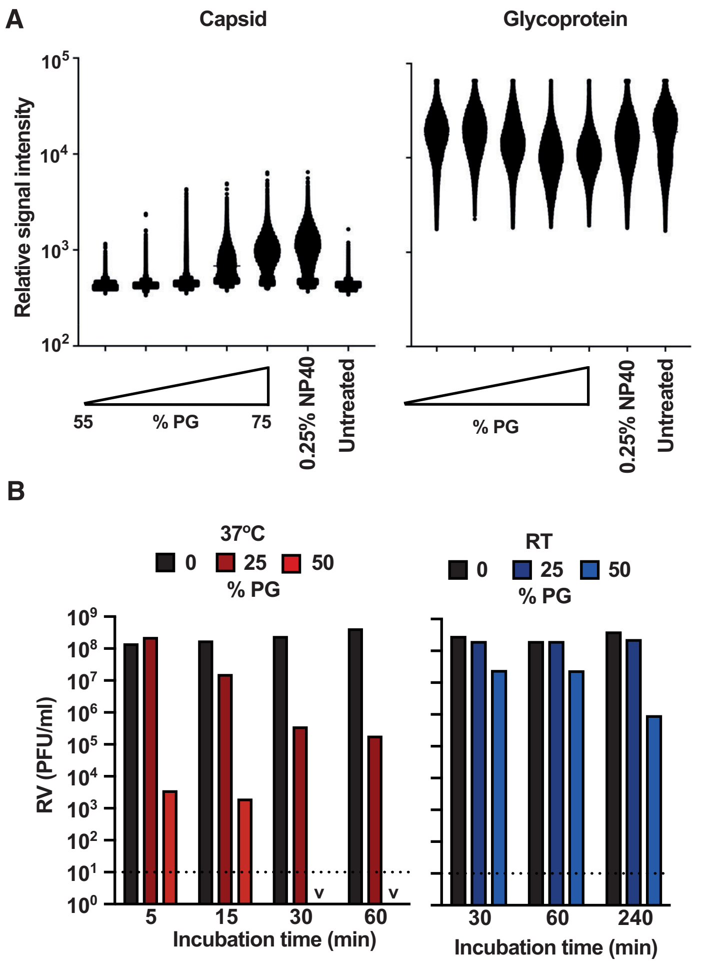


**Figure EV4. PG mediated viral envelope permeabilisation.**

A Virus particle signal intensities of concentrated VSV-G pseudovirus immobilised onto coverslips then incubated with 55–75% [PG] for 5 min at RT ($N = 3$). Pseudovirus particles were stained with anti-capsid and anti-glycoprotein primary antibodies followed by fluorescently labelled secondary antibodies then imaged using the Oxford Nanoimager at 100× oil immersion at 488 and 640 nm.

B Rotavirus was incubated with 0–50% [PG] for 1–240 min at RT or 37°C and infectivity assessed by titration ($N = 2$); PFU = plaque forming units. 2-way ANOVA ([PG] × time), 37°C: [PG] ****$P < 0.0001$, time ****$P < 0.0001$, interaction ****$P < 0.0001$, RT: [PG] ***$P < 0.001$, time ****$P < 0.0001$, interaction ****$P < 0.0001$. v = < $10^1$; dashed line = limit of detection. Representative replicate shown, see Fig 3 for repeat.

Source data are available online for this figure.

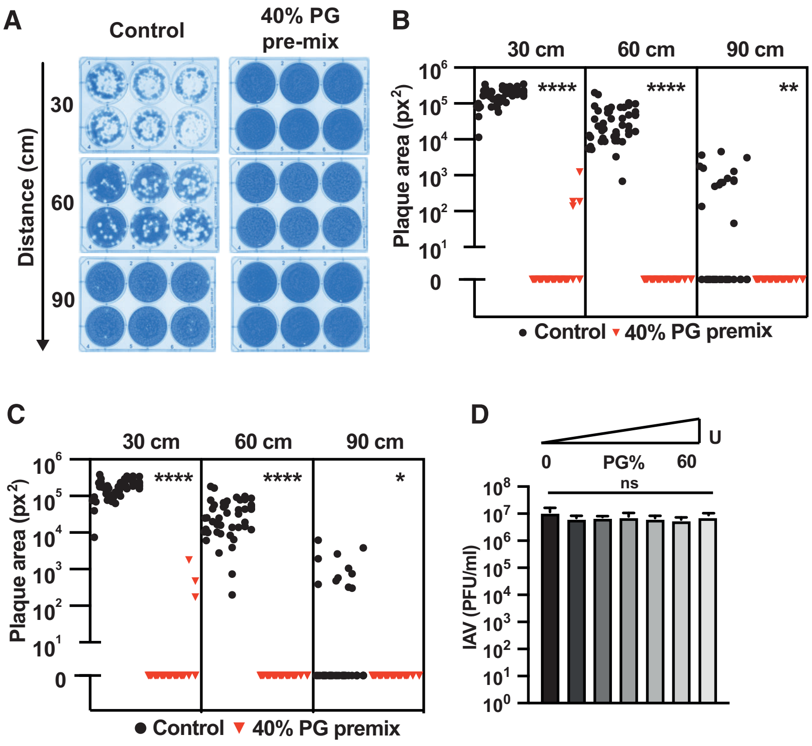


**Figure EV5. Concomitant nebulisation of PG and IAV prevents infection.**

A–C $10^6$ PFU IAV was mixed with PG or PBS control to 40% final concentration and immediately nebulised (< 1 min incubation) into the virus transmission tunnel. (A) Representative plates. Viral plaque area on tissue culture plates at 30 cm, 60 cm and 90 cm were computationally analysed using ImageJ ColonyArea plugin (B) and viral plaque macros (C) ($N$ = 8; $n$ = 6). 2-way ANOVA ([PG] × distance): [PG] ****$P$ < 0.0001, distance ****$P$ < 0.0001, interaction ****$P$ < 0.0001.

D IAV was mixed with PG to 0–60% final concentration and immediately diluted for plaque assay ($N$ = 2; mean ± SD) (< 1 min incubation). 1-way ANOVA [PG] $P$ > 0.05.

Source data are available online for this figure.

 © 2023 The Authors
