# Supplementary figures and images for: Propylene glycol inactivates respiratory viruses and prevents airborne transmission
Source: EMBO Mol Med. 2023 Nov 16;15(12):e17932. doi: 10.15252/emmm.202317932 (PMC10701621; doi:10.15252/emmm.202317932)

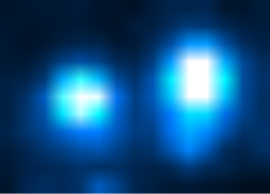

Supplement: Supplementary file 7 — Source Data for Figure 3 [file EMMM-15-e17932-s007.zip › EMM-2023-17932_Figure_3/3C/EMM-2023-17932_0.25%_NP40_capsid.png]

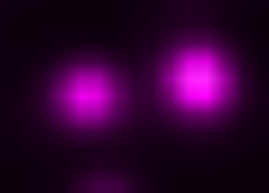

Supplement: Supplementary file 7 — Source Data for Figure 3 [file EMMM-15-e17932-s007.zip › EMM-2023-17932_Figure_3/3C/EMM-2023-17932_0.25%_NP40_glycoprotein.png]

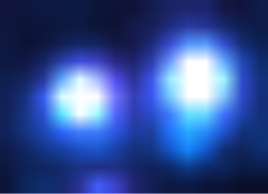

Supplement: Supplementary file 7 — Source Data for Figure 3 [file EMMM-15-e17932-s007.zip › EMM-2023-17932_Figure_3/3C/EMM-2023-17932_0.25%_NP40_merge.png]

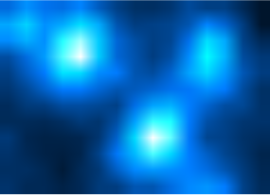

Supplement: Supplementary file 7 — Source Data for Figure 3 [file EMMM-15-e17932-s007.zip › EMM-2023-17932_Figure_3/3C/EMM-2023-17932_70%_PG_capsid.png]

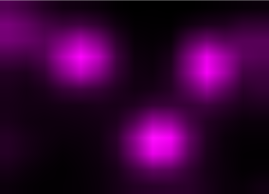

Supplement: Supplementary file 7 — Source Data for Figure 3 [file EMMM-15-e17932-s007.zip › EMM-2023-17932_Figure_3/3C/EMM-2023-17932_70%_PG_glycoprotein.png]

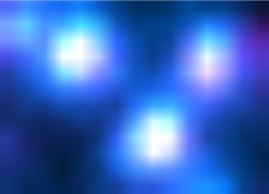

Supplement: Supplementary file 7 — Source Data for Figure 3 [file EMMM-15-e17932-s007.zip › EMM-2023-17932_Figure_3/3C/EMM-2023-17932_70%_PG_merge.png]

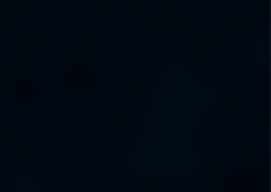

Supplement: Supplementary file 7 — Source Data for Figure 3 [file EMMM-15-e17932-s007.zip › EMM-2023-17932_Figure_3/3C/EMM-2023-17932_untreated_capsid.png]

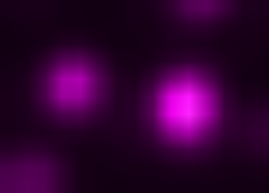

Supplement: Supplementary file 7 — Source Data for Figure 3 [file EMMM-15-e17932-s007.zip › EMM-2023-17932_Figure_3/3C/EMM-2023-17932_untreated_glycoprotein.png]

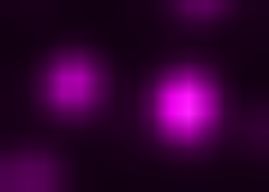

Supplement: Supplementary file 7 — Source Data for Figure 3 [file EMMM-15-e17932-s007.zip › EMM-2023-17932_Figure_3/3C/EMM-2023-17932_untreated_merge.png]

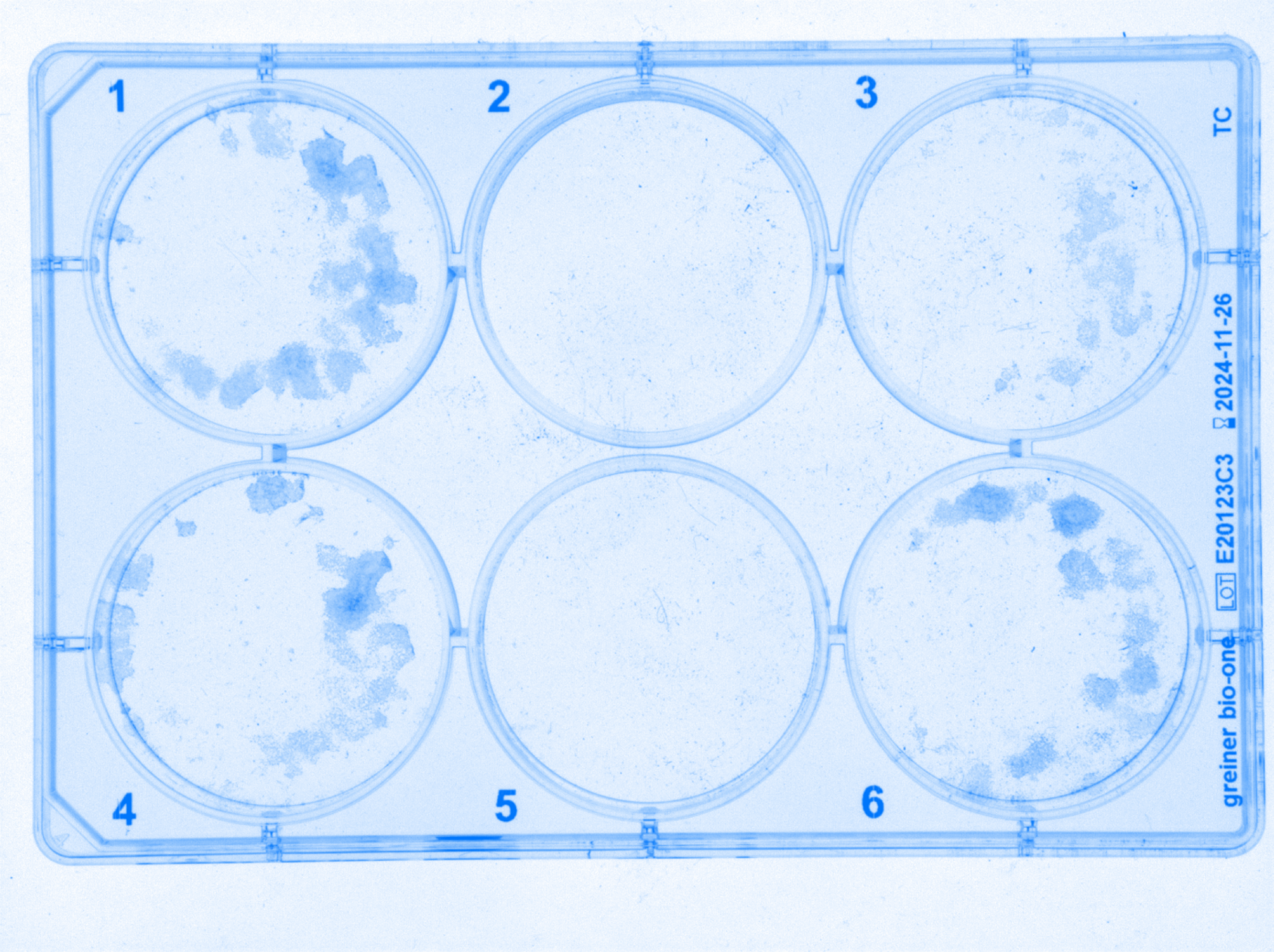

Supplement: Supplementary file 8 — Source Data for Figure 4 [file EMMM-15-e17932-s001.zip › EMM-2023-17932_Figure_4/4B/EMM-2023-17932_0_mg_PG_L_air_p1_IAV.tif]

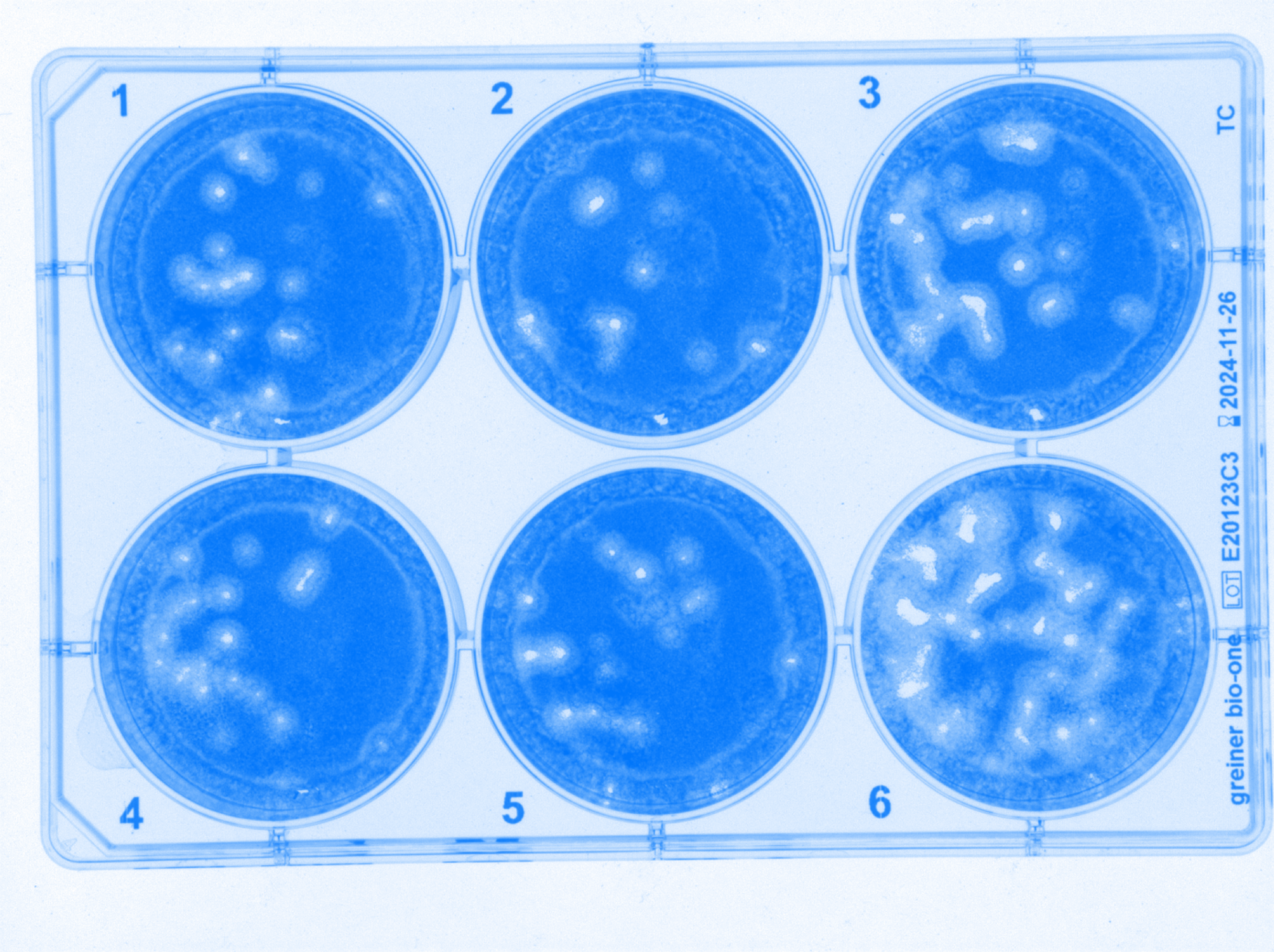

Supplement: Supplementary file 8 — Source Data for Figure 4 [file EMMM-15-e17932-s001.zip › EMM-2023-17932_Figure_4/4B/EMM-2023-17932_0_mg_PG_L_air_p2_IAV.tif]

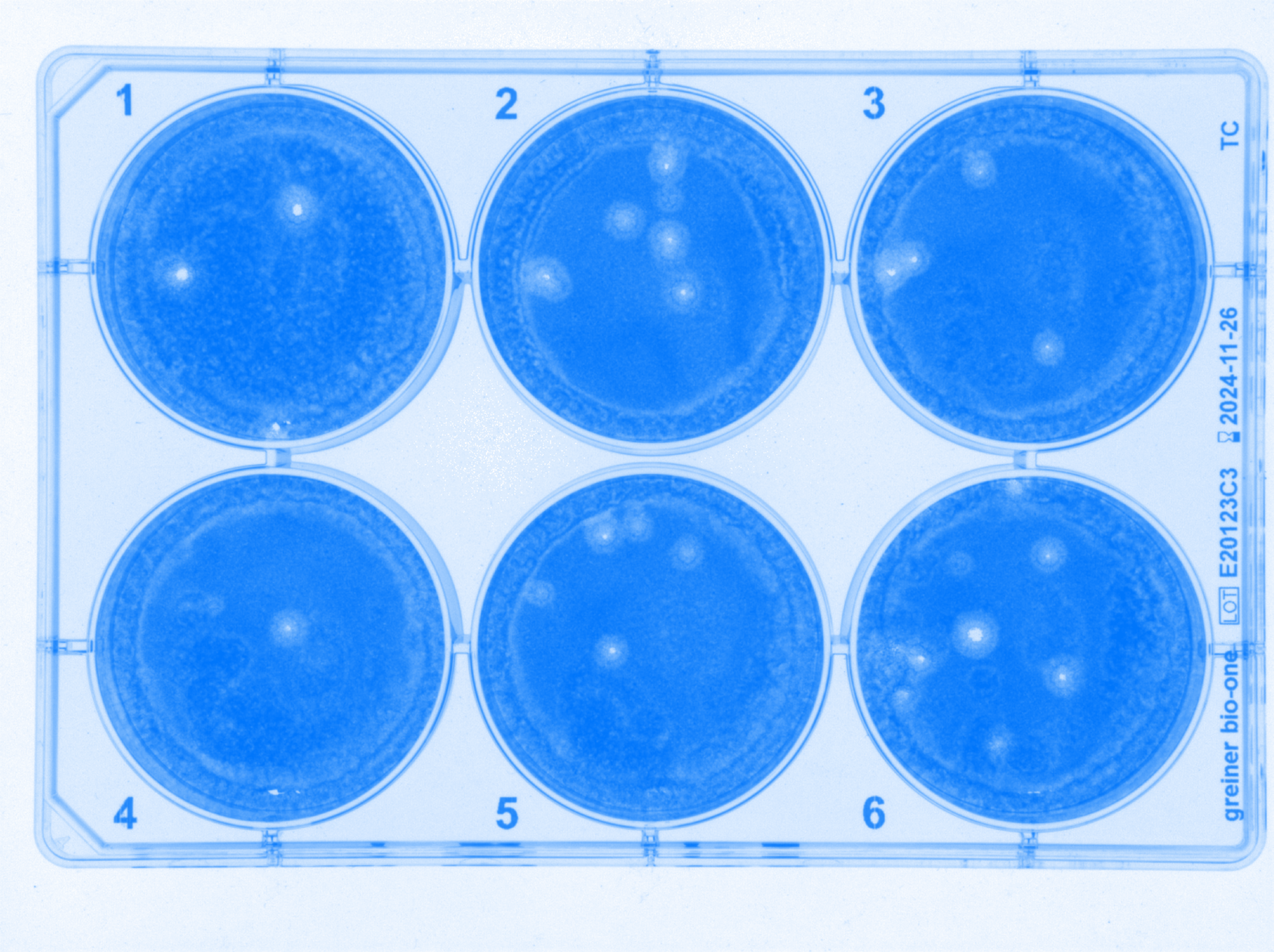

Supplement: Supplementary file 8 — Source Data for Figure 4 [file EMMM-15-e17932-s001.zip › EMM-2023-17932_Figure_4/4B/EMM-2023-17932_0_mg_PG_L_air_p3_IAV.tif]

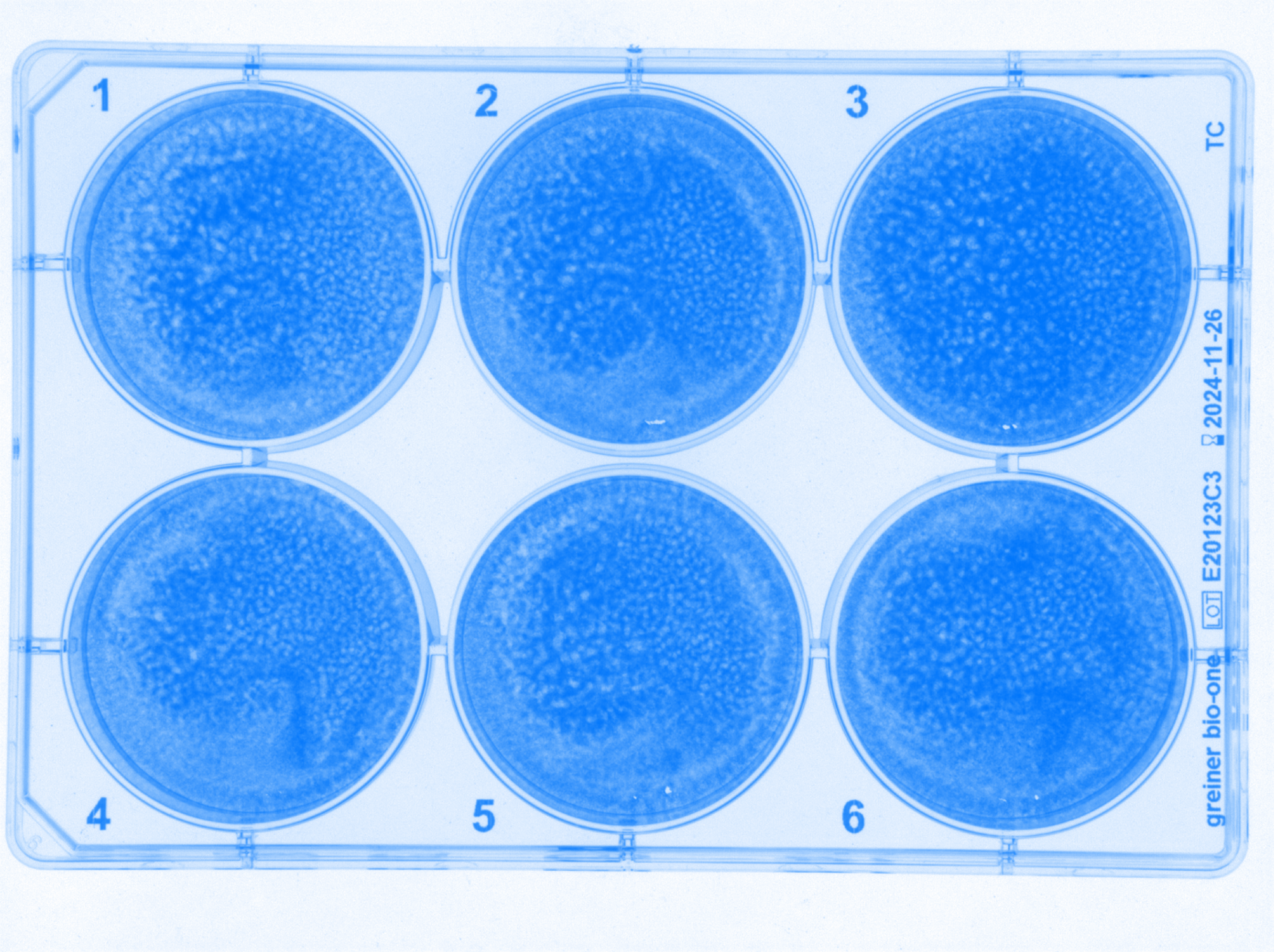

Supplement: Supplementary file 8 — Source Data for Figure 4 [file EMMM-15-e17932-s001.zip › EMM-2023-17932_Figure_4/4B/EMM-2023-17932_11_mg_PG_L_air_p1_IAV.tif]

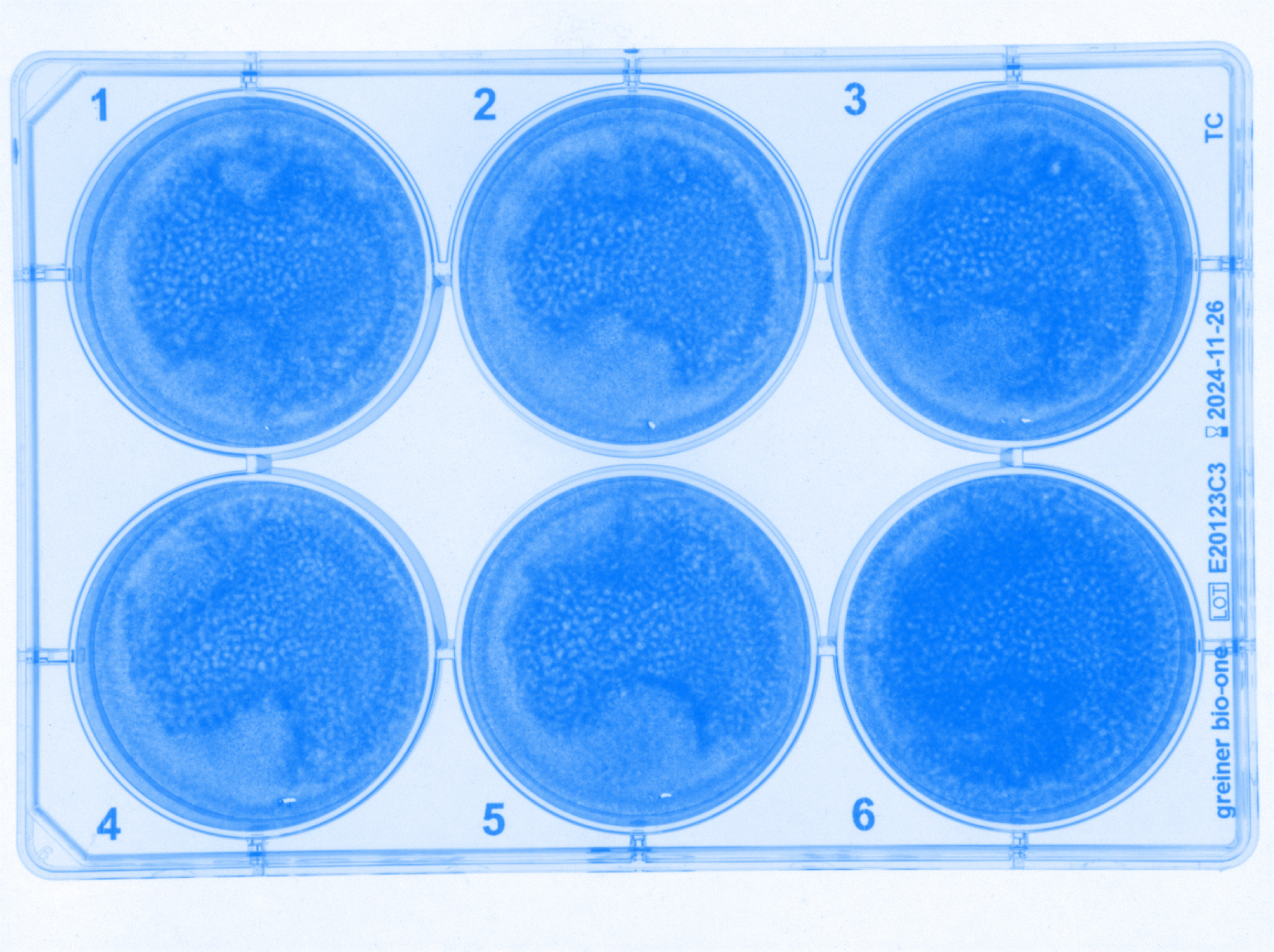

Supplement: Supplementary file 8 — Source Data for Figure 4 [file EMMM-15-e17932-s001.zip › EMM-2023-17932_Figure_4/4B/EMM-2023-17932_11_mg_PG_L_air_p2_IAV.tif]

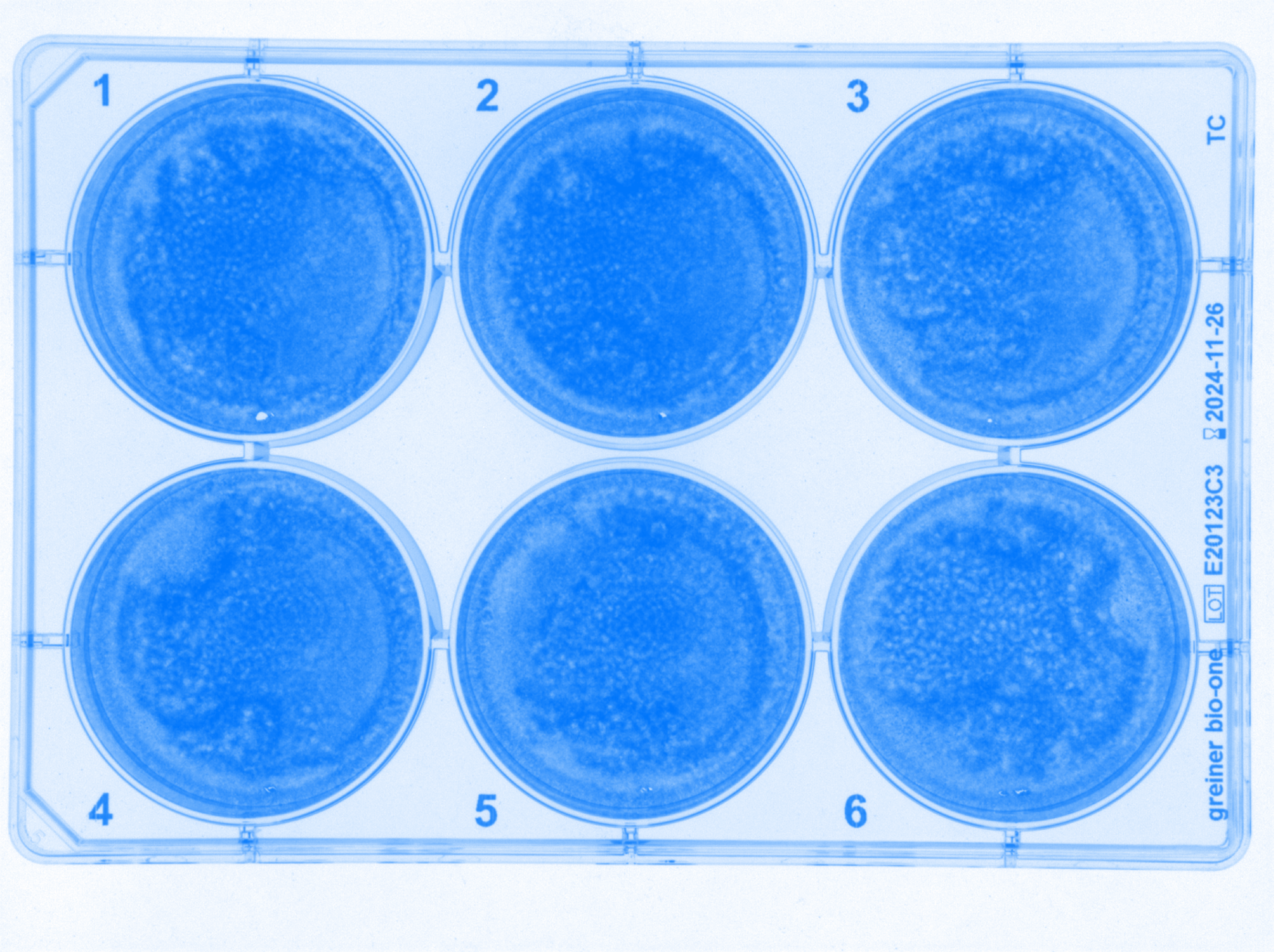

Supplement: Supplementary file 8 — Source Data for Figure 4 [file EMMM-15-e17932-s001.zip › EMM-2023-17932_Figure_4/4B/EMM-2023-17932_11_mg_PG_L_air_p3_IAV.tif]

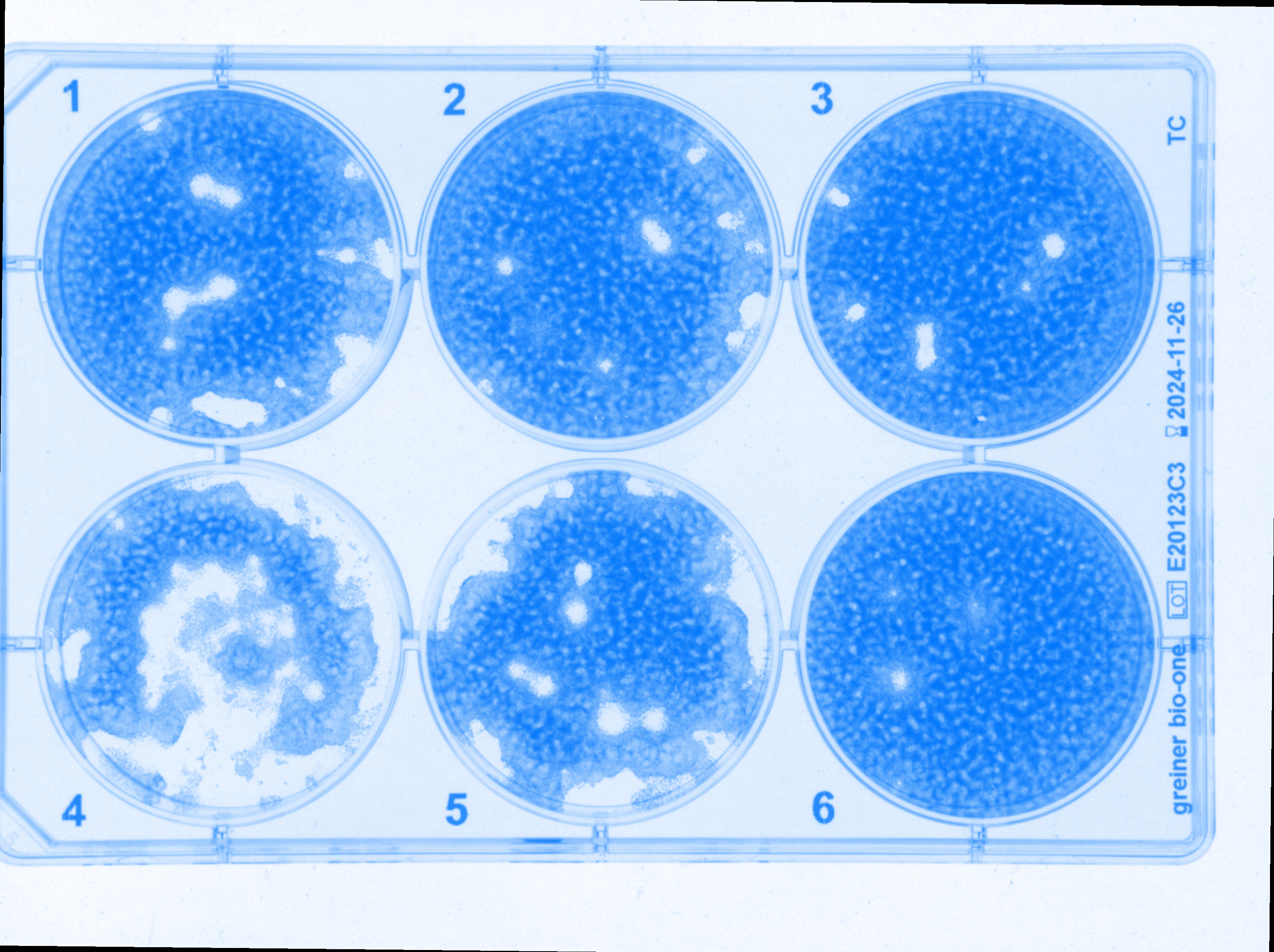

Supplement: Supplementary file 8 — Source Data for Figure 4 [file EMMM-15-e17932-s001.zip › EMM-2023-17932_Figure_4/4B/EMM-2023-17932_2.9_mg_PG_L_air_p1_IAV.tif]

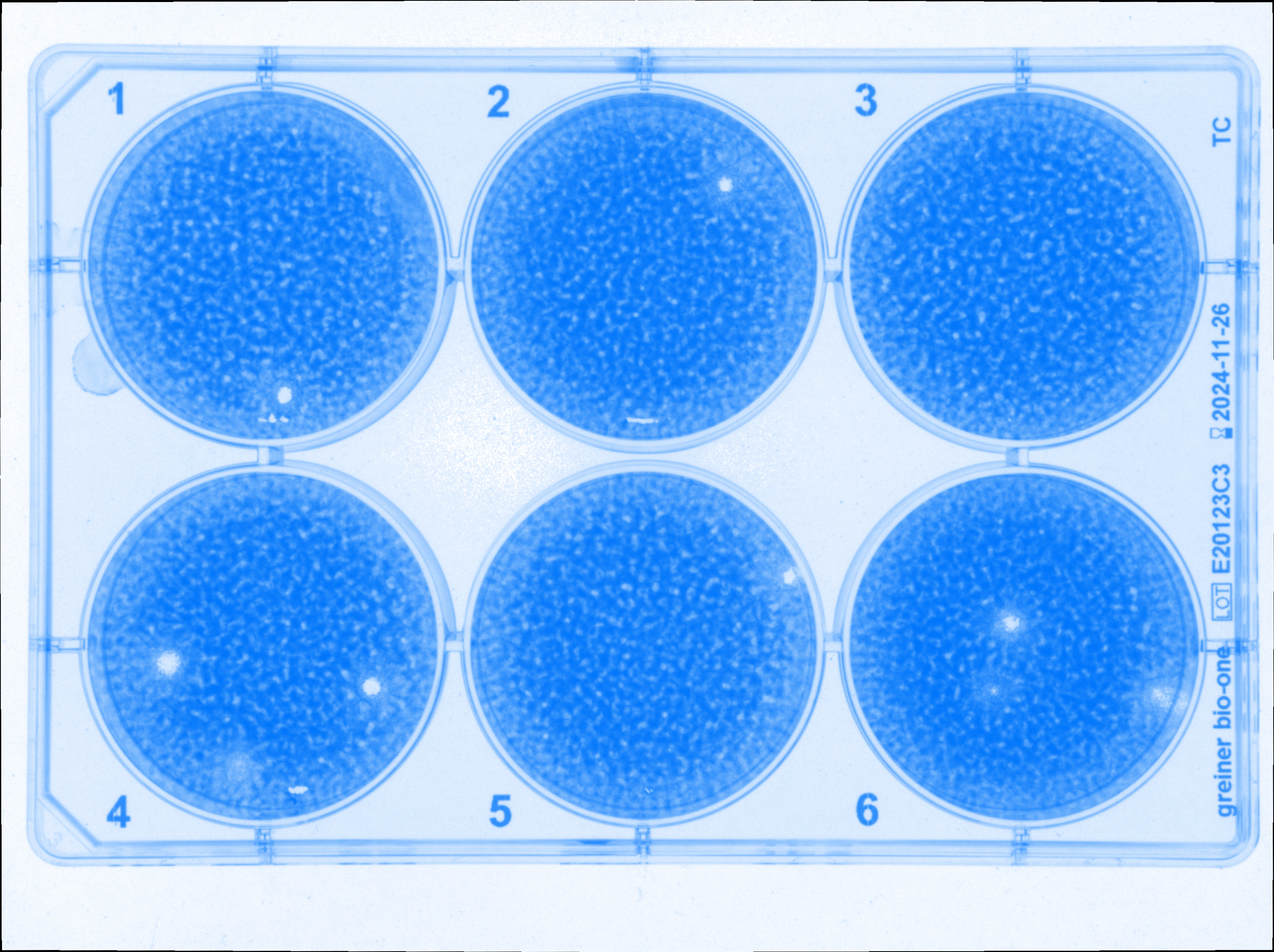

Supplement: Supplementary file 8 — Source Data for Figure 4 [file EMMM-15-e17932-s001.zip › EMM-2023-17932_Figure_4/4B/EMM-2023-17932_2.9_mg_PG_L_air_p2_IAV.tif]

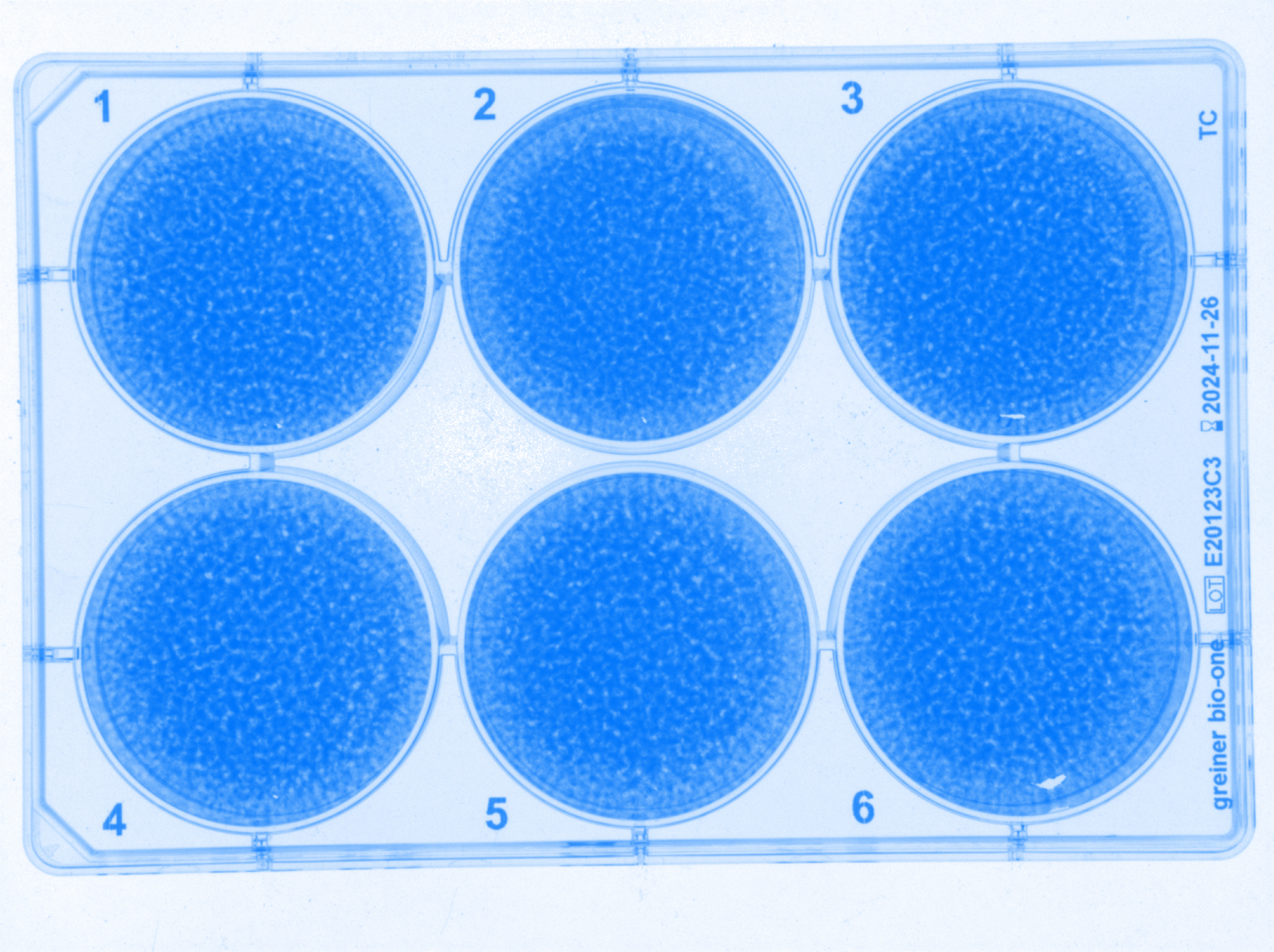

Supplement: Supplementary file 8 — Source Data for Figure 4 [file EMMM-15-e17932-s001.zip › EMM-2023-17932_Figure_4/4B/EMM-2023-17932_2.9_mg_PG_L_air_p3_IAV.tif]

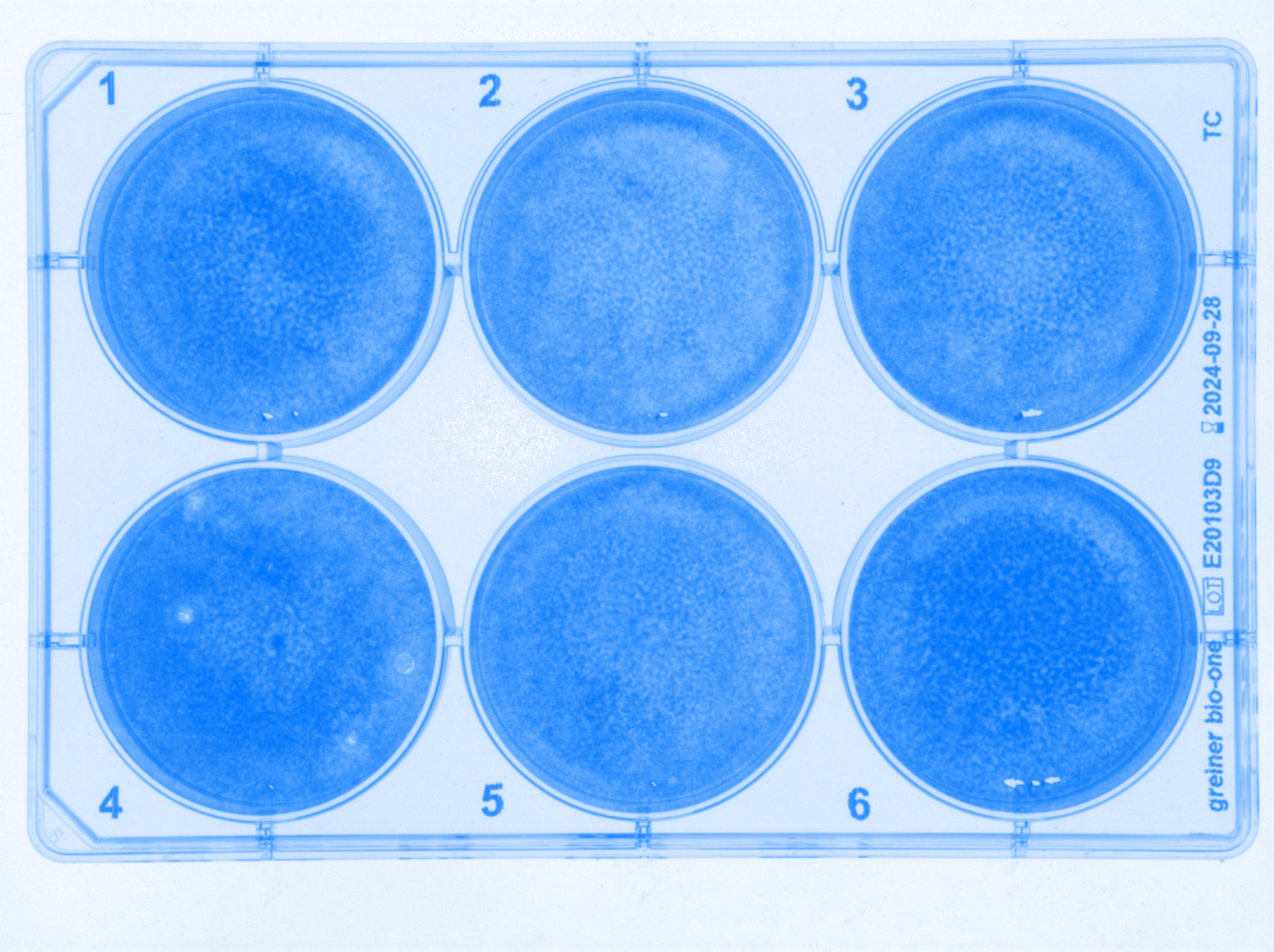

Supplement: Supplementary file 8 — Source Data for Figure 4 [file EMMM-15-e17932-s001.zip › EMM-2023-17932_Figure_4/4B/EMM-2023-17932_6.5_mg_PG_L_air_p1_IAV.tif]

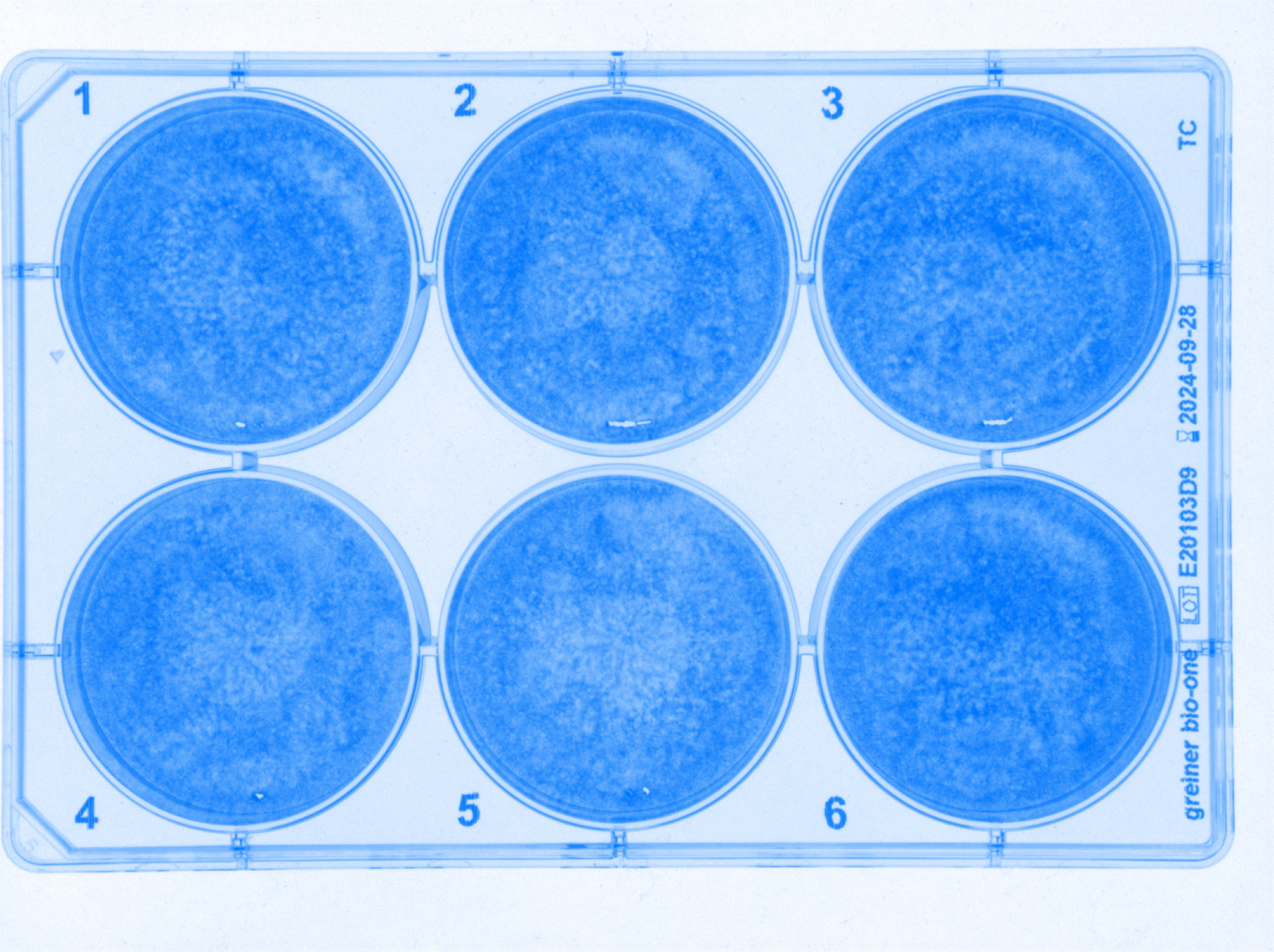

Supplement: Supplementary file 8 — Source Data for Figure 4 [file EMMM-15-e17932-s001.zip › EMM-2023-17932_Figure_4/4B/EMM-2023-17932_6.5_mg_PG_L_air_p2_IAV.tif]

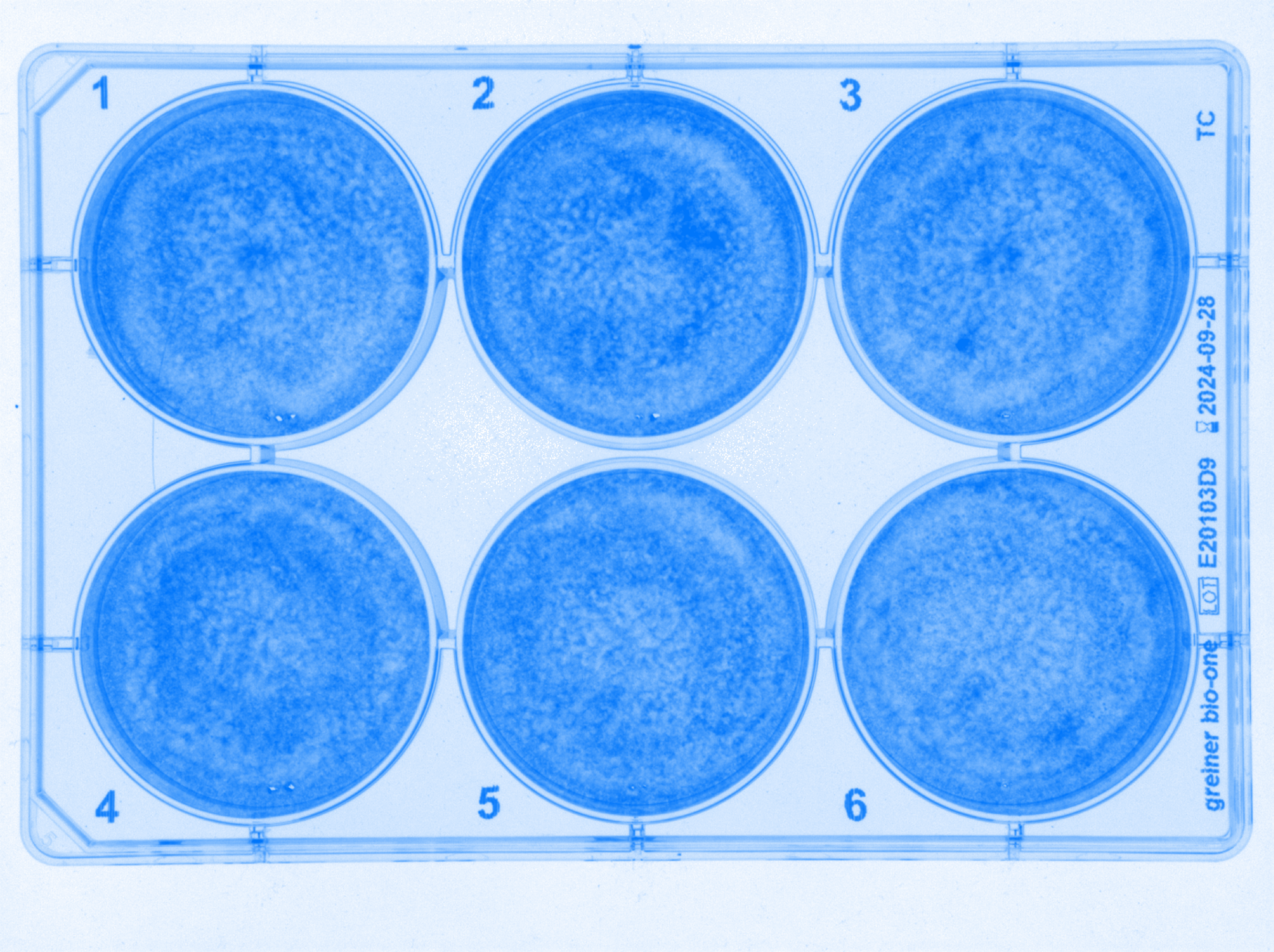

Supplement: Supplementary file 8 — Source Data for Figure 4 [file EMMM-15-e17932-s001.zip › EMM-2023-17932_Figure_4/4B/EMM-2023-17932_6.5_mg_PG_L_air_p3_IAV.tif]

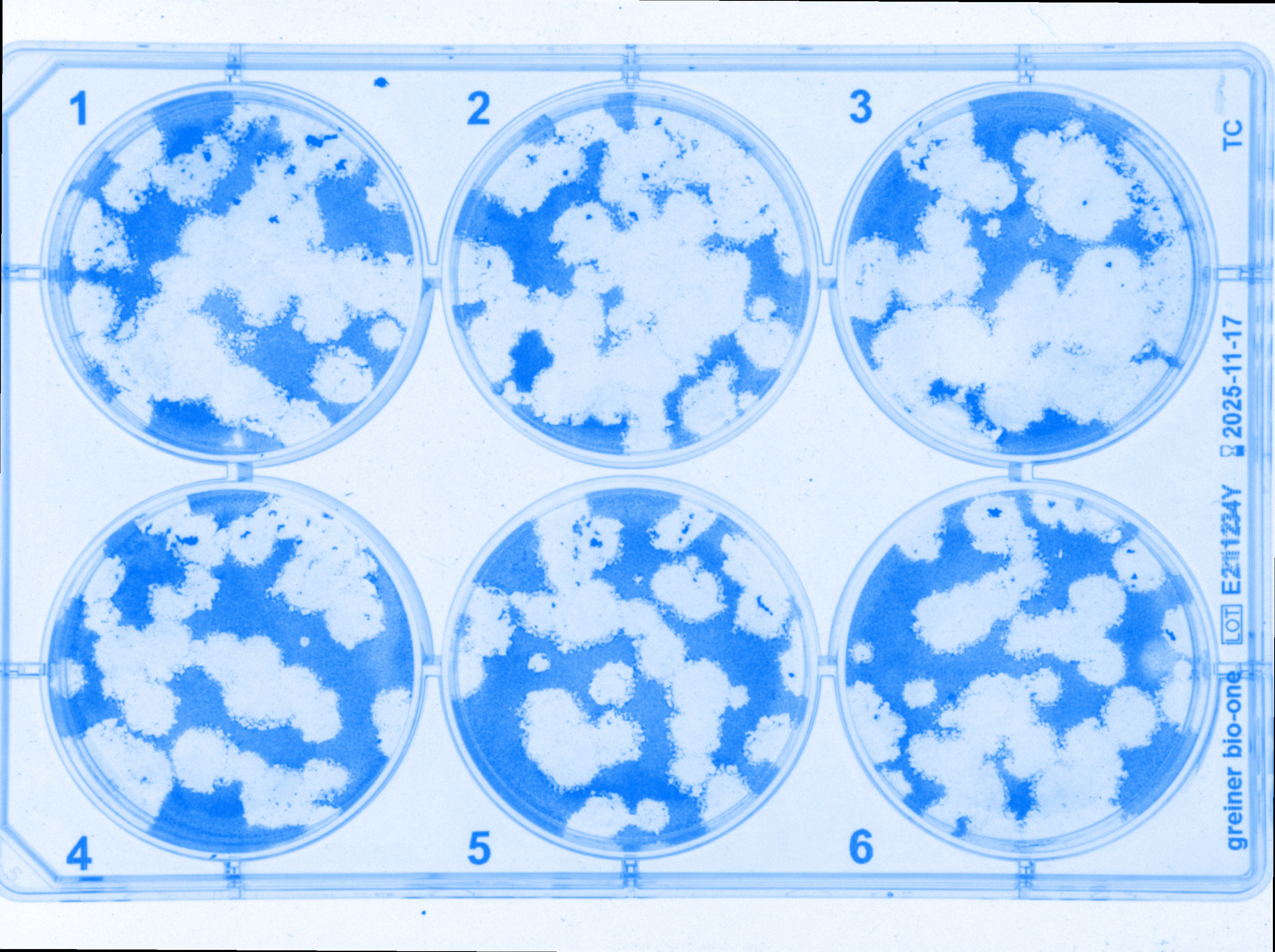

Supplement: Supplementary file 8 — Source Data for Figure 4 [file EMMM-15-e17932-s001.zip › EMM-2023-17932_Figure_4/4E/EMM-2023-17932_0_mg_L_air_PG_p1.tif]

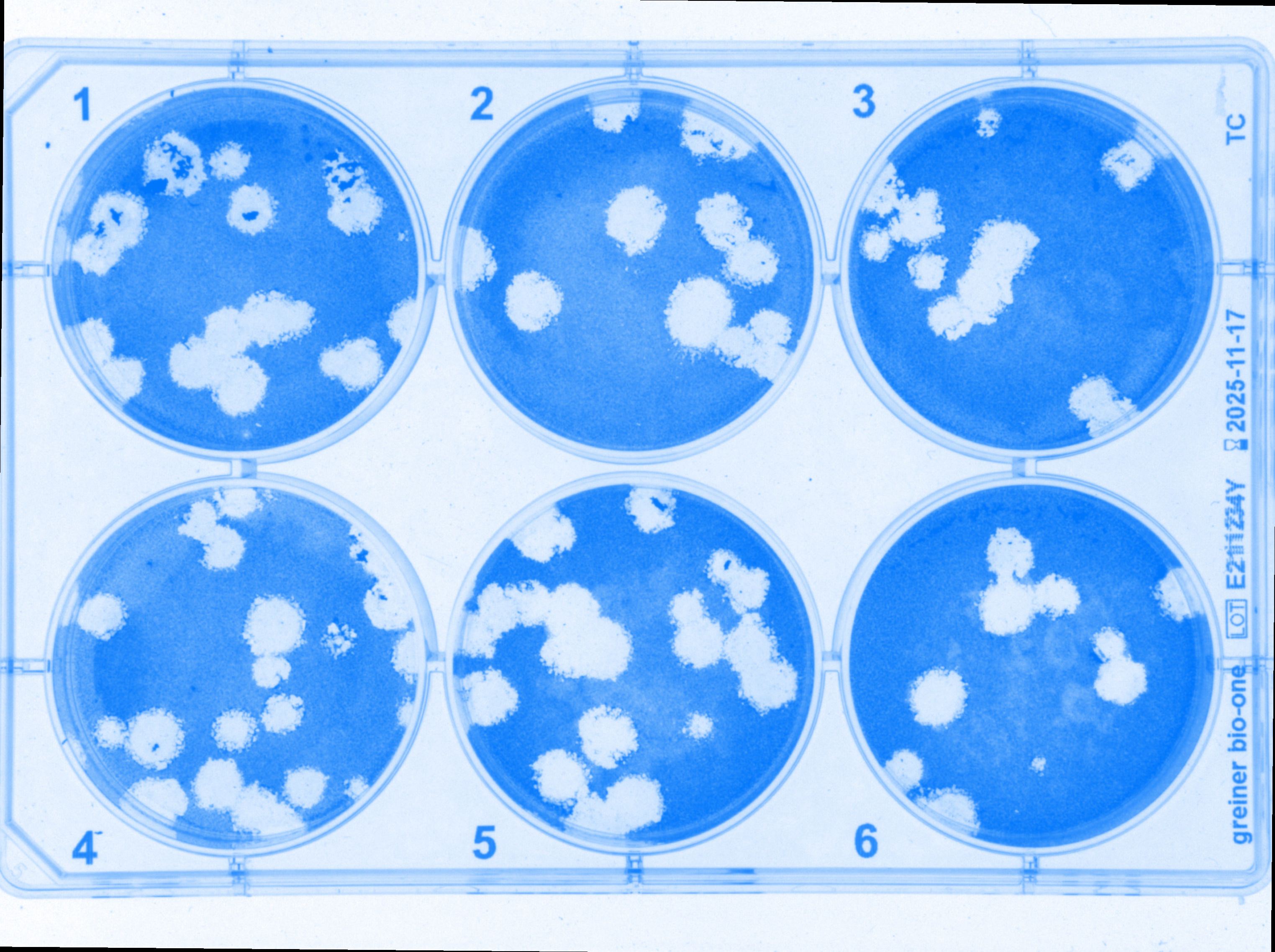

Supplement: Supplementary file 8 — Source Data for Figure 4 [file EMMM-15-e17932-s001.zip › EMM-2023-17932_Figure_4/4E/EMM-2023-17932_0_mg_L_air_PG_p2.tif]

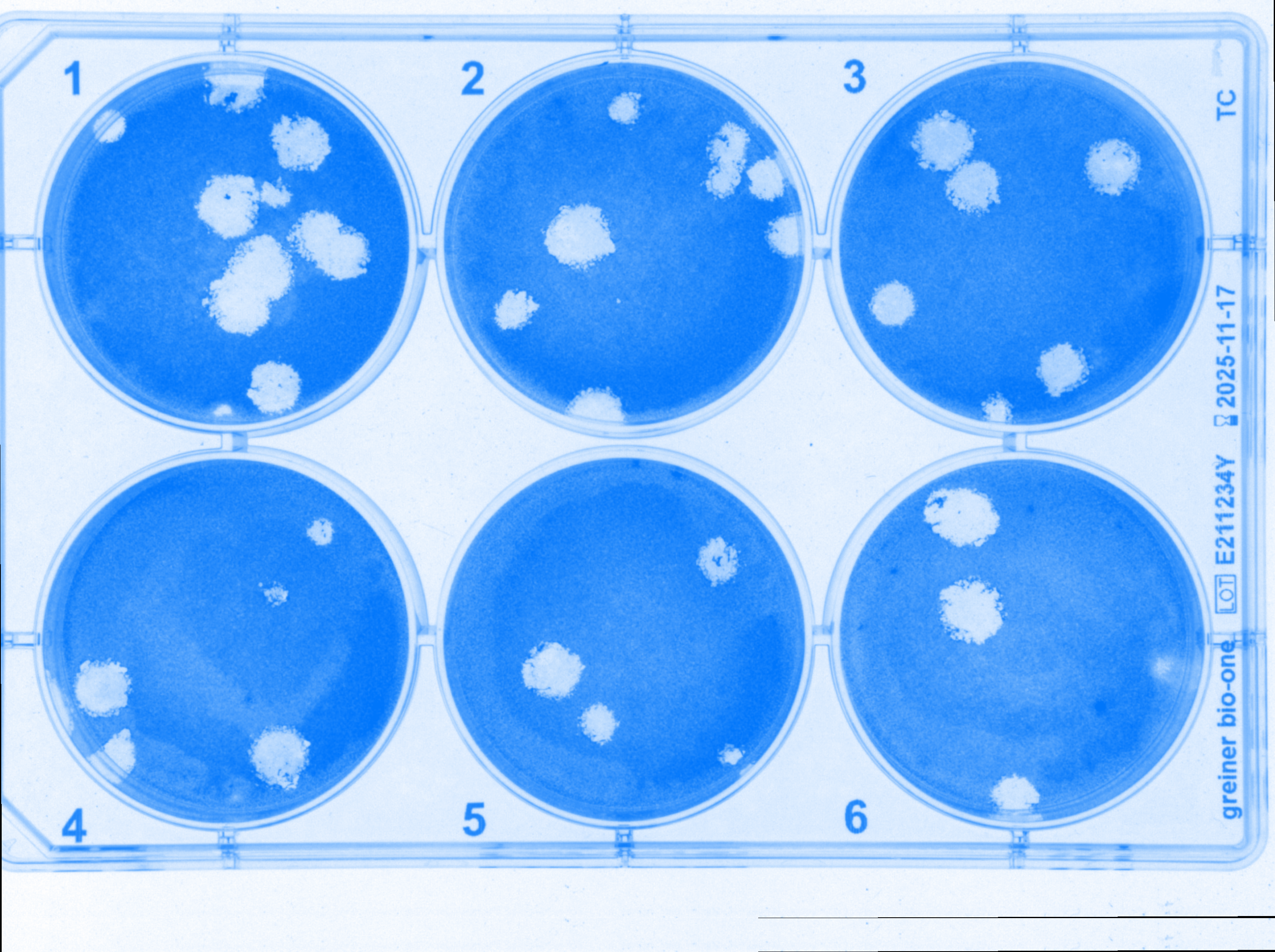

Supplement: Supplementary file 8 — Source Data for Figure 4 [file EMMM-15-e17932-s001.zip › EMM-2023-17932_Figure_4/4E/EMM-2023-17932_0_mg_L_PG_p3.tif]

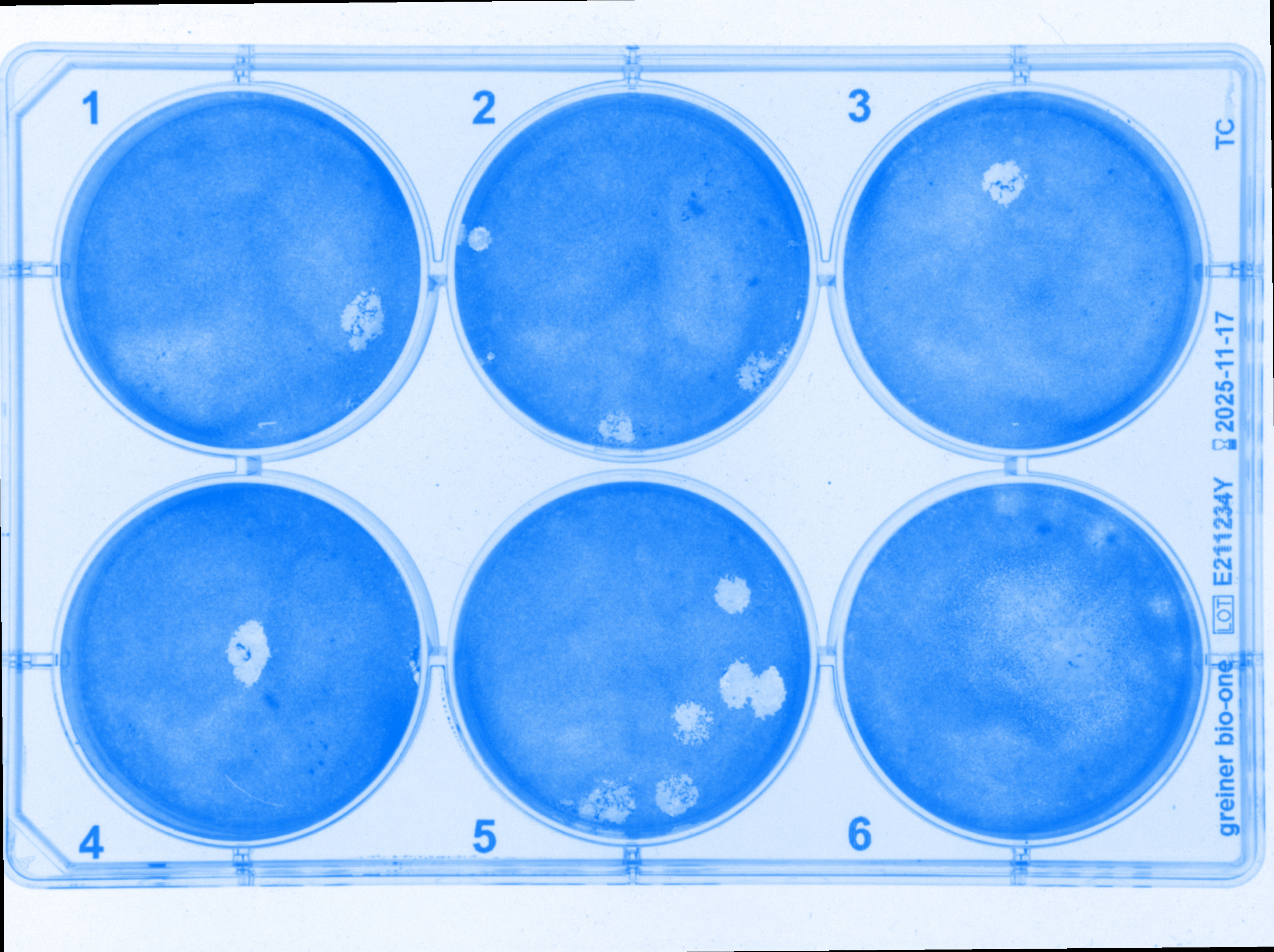

Supplement: Supplementary file 8 — Source Data for Figure 4 [file EMMM-15-e17932-s001.zip › EMM-2023-17932_Figure_4/4E/EMM-2023-17932_11_mg_PG_L_air_p1.tif]

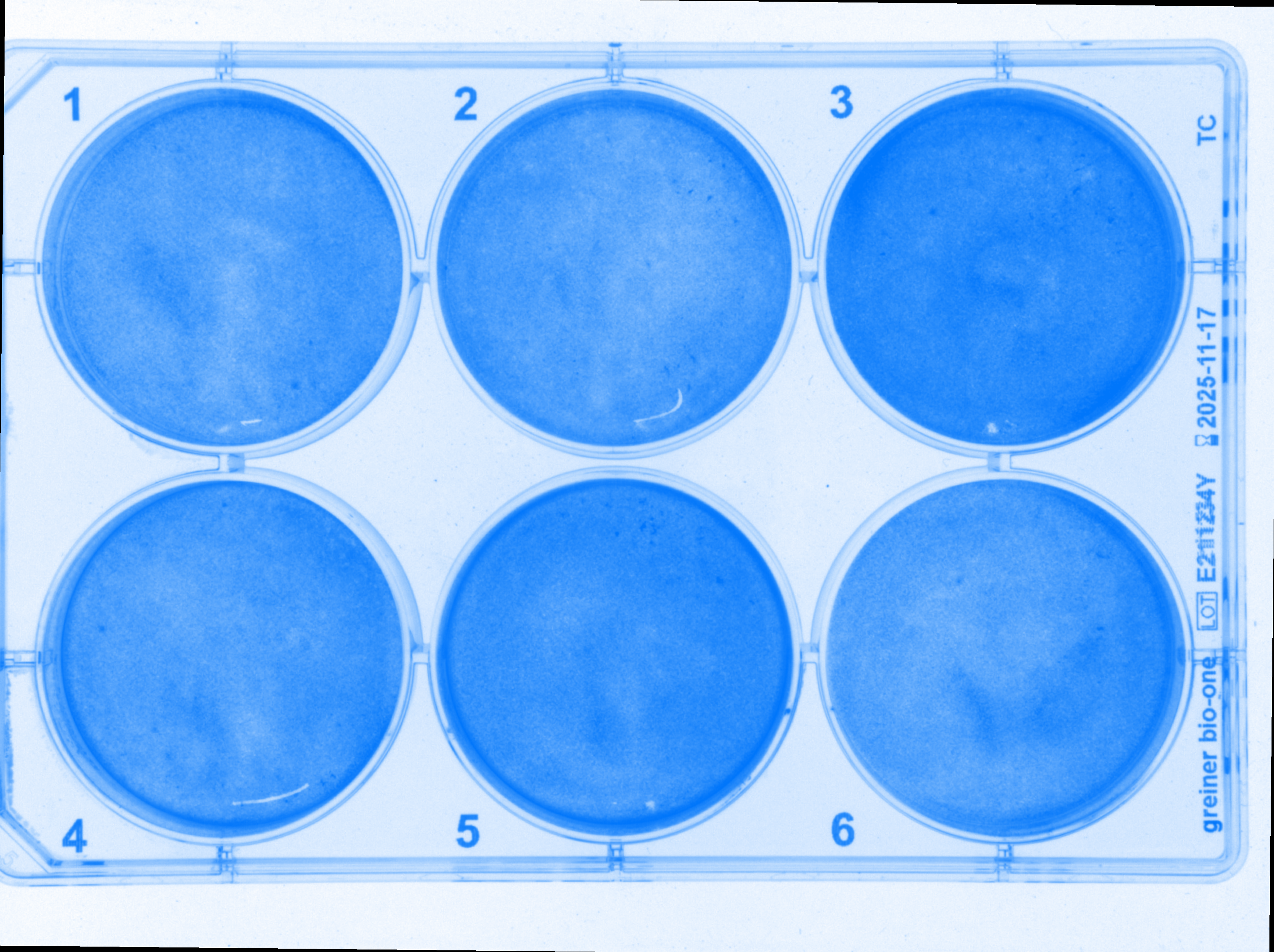

Supplement: Supplementary file 8 — Source Data for Figure 4 [file EMMM-15-e17932-s001.zip › EMM-2023-17932_Figure_4/4E/EMM-2023-17932_11_mg_PG_L_air_p2.tif]

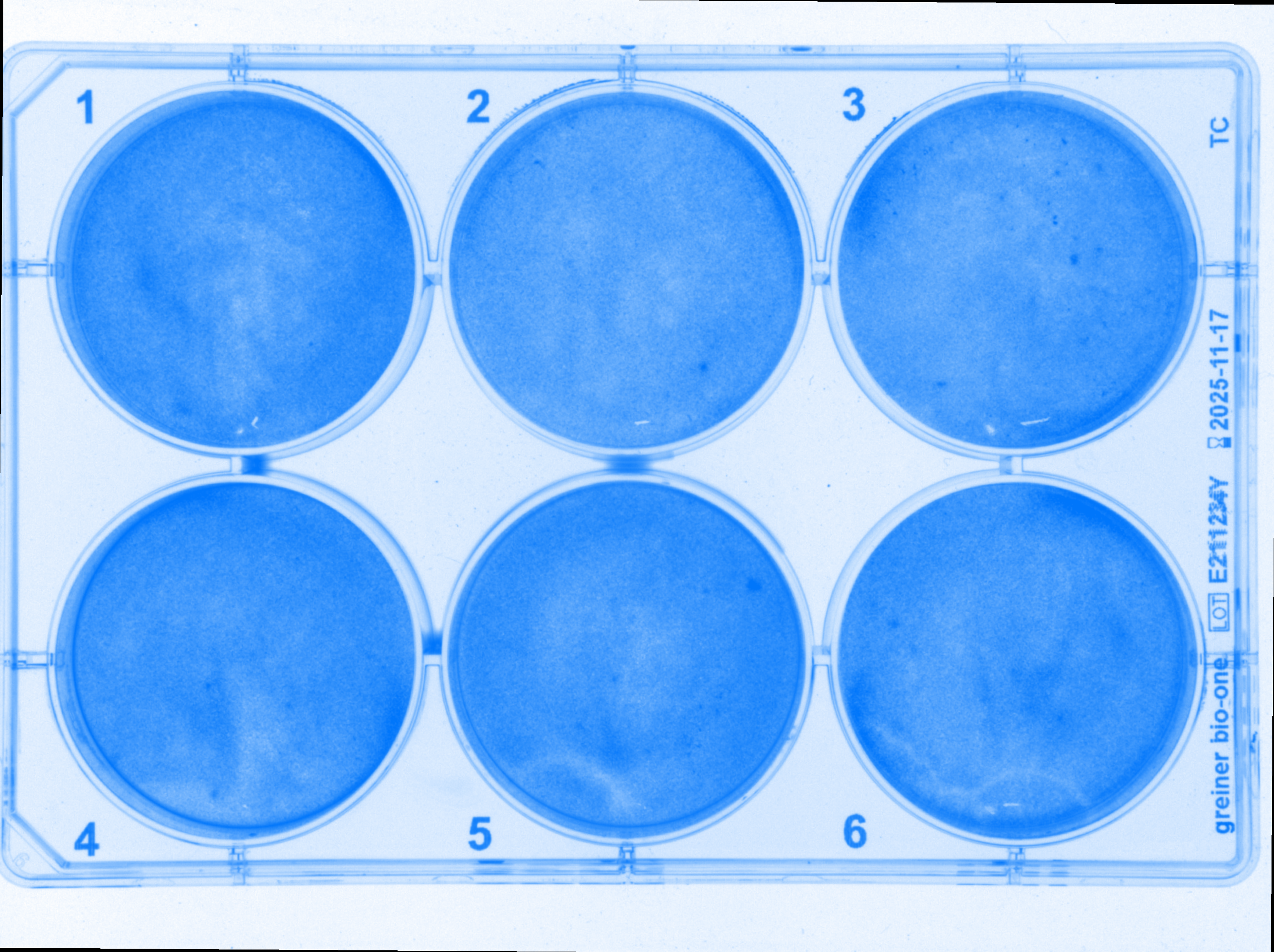

Supplement: Supplementary file 8 — Source Data for Figure 4 [file EMMM-15-e17932-s001.zip › EMM-2023-17932_Figure_4/4E/EMM-2023-17932_11_mg_PG_L_air_p3.tif]

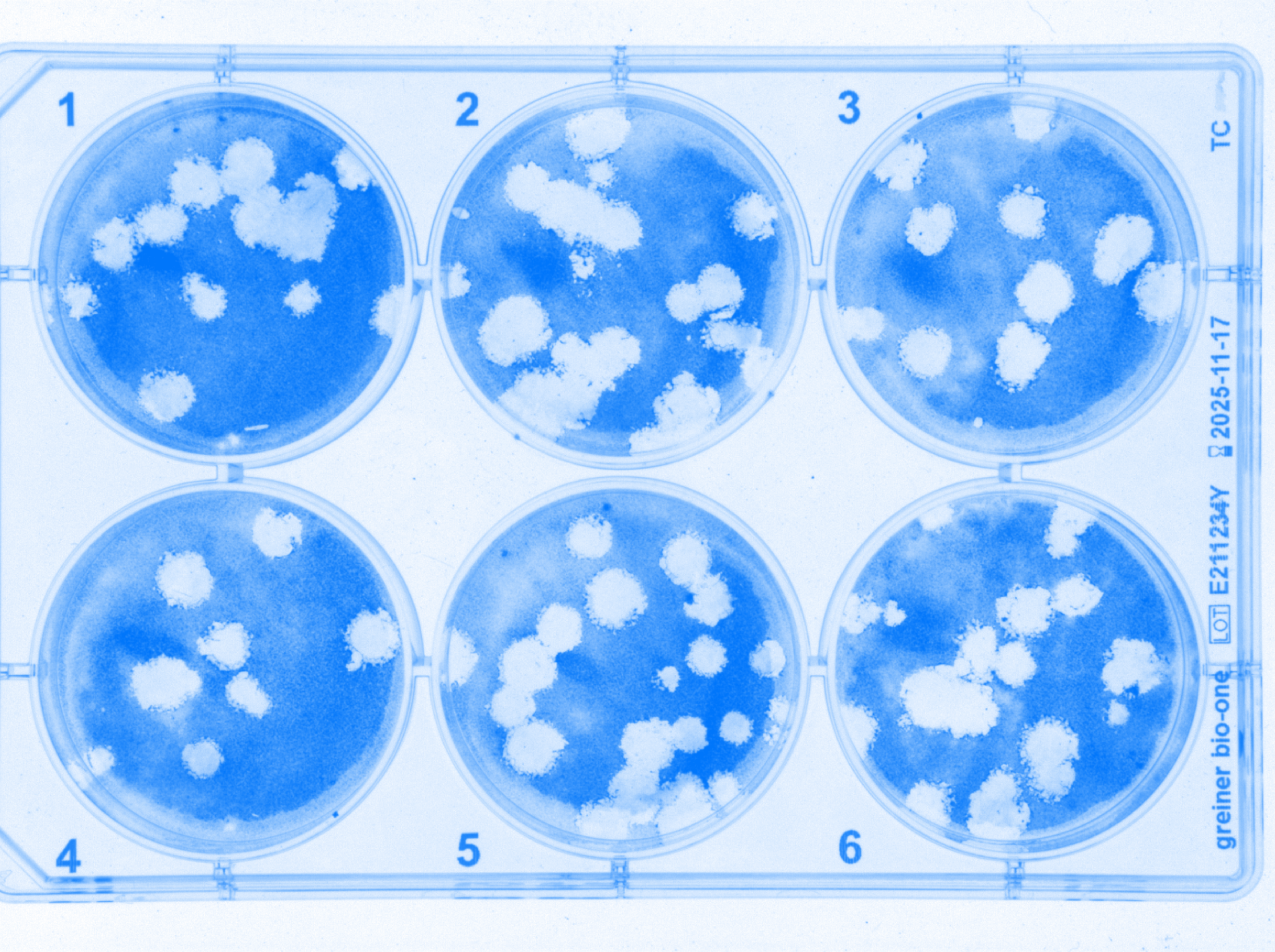

Supplement: Supplementary file 8 — Source Data for Figure 4 [file EMMM-15-e17932-s001.zip › EMM-2023-17932_Figure_4/4E/EMM-2023-17932_2.9_mg_PG_L_air_p1.tif]

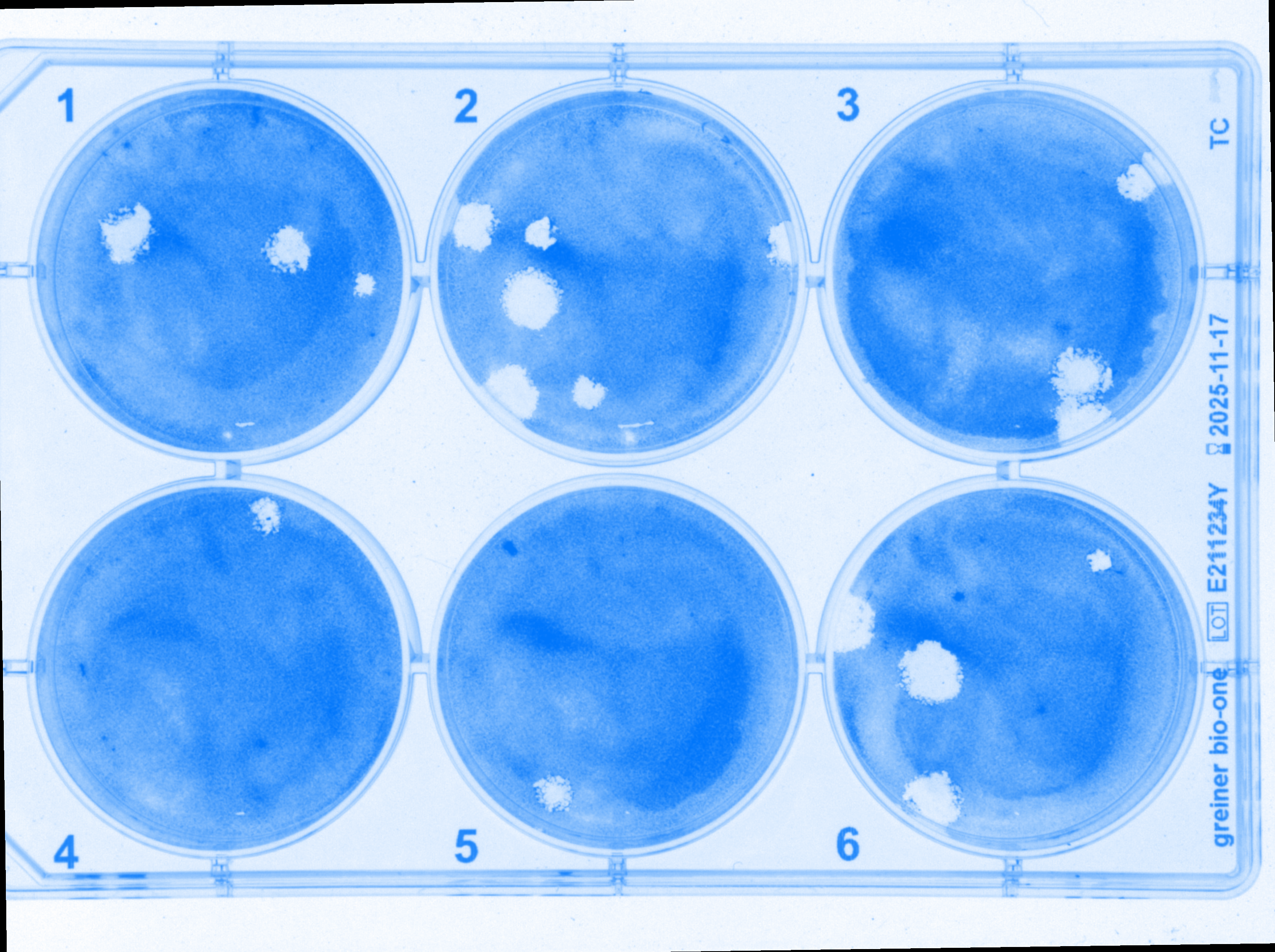

Supplement: Supplementary file 8 — Source Data for Figure 4 [file EMMM-15-e17932-s001.zip › EMM-2023-17932_Figure_4/4E/EMM-2023-17932_2.9_mg_PG_L_air_p2.tif]

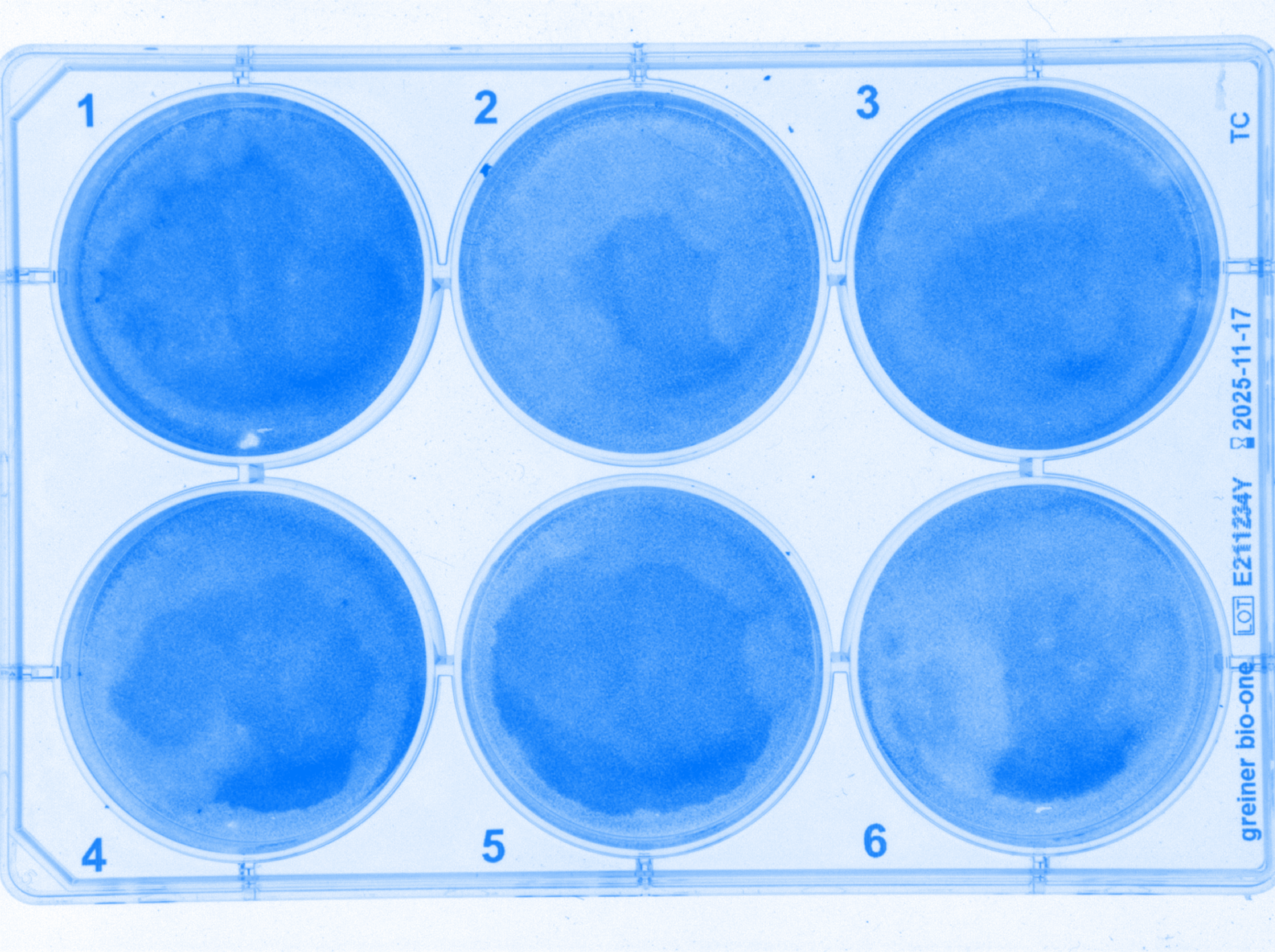

Supplement: Supplementary file 8 — Source Data for Figure 4 [file EMMM-15-e17932-s001.zip › EMM-2023-17932_Figure_4/4E/EMM-2023-17932_2.9_mg_PG_L_air_p3.tif]
