# Supplementary material for: Propylene glycol inactivates respiratory viruses and prevents airborne transmission
Source: EMBO Mol Med. 2023 Nov 16;15(12):e17932. doi: 10.15252/emmm.202317932 (PMC10701621; doi:10.15252/emmm.202317932)
Supplement: Supplementary file 9 [file EMMM-15-e17932-s005.pdf]

EMBO Molecular Medicine

Propylene glycol inactivates respiratory viruses and prevents airborne transmission

Christine Styles, Jie Zhou, katie flight, Jonathan Brown, Charlotte Lewis, Xinyu Wang, Michael Vanden Oever, Thomas Peacock, Ziyin Wang, Rosie Millns, John O'Neill, Alexander Borodavka, Joe Grove, Wendy Barclay, John Tregoning, and Rachel Edgar
DOI: 10.15252/emmm.202317932

Corresponding author: Rachel Edgar (rachel.edgar@imperial.ac.uk)

Review Timeline:

Transferred from Review Commons:	28th Apr 23
Editorial Decision:	3rd May 23
Revision Received:	9th Sep 23
Editorial Decision:	18th Sep 23
Revision Received:	16th Oct 23
Accepted:	17th Oct 23

Editor: Zeljko Durdevic

Transaction Report:

This manuscript was transferred to EMBO Molecular Medicine following peer review at Review Commons.

(Note: With the exception of the correction of typographical or spelling errors that could be a source of ambiguity, letters and reports are not edited. Depending on transfer agreements, referee reports obtained elsewhere may or may not be included in this compilation. Referee reports are anonymous unless the Referee chooses to sign their reports.)

Review #1

1. Evidence, reproducibility and clarity:

Evidence, reproducibility and clarity (Required)

Summary

This paper by Styles et al describes the effect of propylene glycol (PG) on a range of enveloped viruses, focusing mainly on IAV and SARS-Cov2. The direct virucidal effect is shown both in vitro and in vivo, as well as the effect of vaporized PG on airborne IAV and SARS-Cov-2. This is a well-performed and very clear study.

Major comments

The claims and conclusions are in general well supported by the data, yet I have some comments.
- Interestingly, there were variations in the effect of PG on the different pseudoviruses. Surprising as I assumed that the effect of PG would be mainly on the lipid envelope, as you also mention in the discussion. It is suggested that PG therefore also has an effect on the conformation of the surface proteins. This can be easily tested, does the addition of PG result in a changed recognition by antibodies targeting these surface proteins? This information would be very valuable for your manuscript.
- Figure S1 shows that although the clinical scores are better, the viral load is not reduced by the addition of PG to the inoculum. This is not mentioned in the main text. This seems very relevant information and should be presented in the main text. Can you please elaborate on this.
- Optional: It would be very interesting to see the effect of vaporized PG on viral transmission between mice/ferrets of a highly infectious virus (eg Sendai virus). This would give an idea if the PG also works on more biological respiratory droplets.

Minor comments

- The caption of Fig1D mentions that the mice are inoculated with 50ml total volume. I presume this should be µl.
- Please put the axis of the viral titer graph now in Fig s1 in PFU/lung or nasal cavity? This would be more informative.
- Typo in 'PG has broad-spectrum virucidal activity' paragraph: titre should be titer.
- Is allergy to PG an issue that could have an effect on further development of this antiviral strategy?

2. Significance:

Significance (Required)

This study is highly relevant. It proposes an easy and possibly efficacious method to prevent airborne transmission of a broad range of (enveloped) viruses.

A strength of this study is the set-up with the virus transmission tunnel. This nicely shows the effect of the vaporized PG on airborne IAV and SARS-Cov-2. A limitation is that the researchers did not analyze non-enveloped viruses, which should be done in a follow-up study.

There does not seem to be any recent research investigating this compound as an antiviral tool. Although the compound was tested for its antimicrobial properties in the 1940s, this has not been continued for unclear reasons (?). This is very valuable research in this world recovering from the recent SARS-Cov-2 pandemic. More mechanistic studies need to be performed to understand how this compound exerts its antiviral and antimicrobial effect.

A more specialized audience will be interested in this study.

My field of expertise: RSV, PIV, antivirals

3. How much time do you estimate the authors will need to complete the suggested revisions:

Estimated time to Complete Revisions (Required)

(Decision Recommendation)

Between 1 and 3 months

4. *Review Commons* values the work of reviewers and encourages them to get credit for their work. Select 'Yes' below to register your reviewing activity at Web of Science Reviewer Recognition Service (formerly Publons); note that the content of your review will not be visible on Web of Science.

Web of Science Reviewer Recognition

Yes

Review #2

1. Evidence, reproducibility and clarity:

Evidence, reproducibility and clarity (Required)

Summary

This study investigates the inactivation potential of propylene glycol against a number of important human respiratory viruses, including influenza A virus and SARS-CoV-2. Potent antiviral activity of propylene glycol was demonstrated for viruses in bulk solutions, in 2uL droplets on a variety of surfaces, and within aerosols. Additionally, propylene glycol was added to viruses prior to infection of live animals, and this treatment significantly improved animal survival outcomes and reduced their weight loss and clinical scores compared to animals infected with non-treated virus. Levels of propylene glycol utilised in the study were substantially less than those classified as well-tolerated by mammals. Overall, authors demonstrate that propylene glycol is a strong yet safe virucide that could be used to control the spread of viral infections.

Major comments

Claims and conclusions are very well-supported by the data presented. Both the data presentation and method details are sufficient to reproduce the experiments. Authors might like to add precise gram concentrations of PG in addition to the percentages listed in the manuscript, either g/L or g/kg as appropriate. This would also allow easier comparison of the tested PG does to those published as safe doses for mammals, which are often given as g/kg.

Replicates are more than adequate, and statistical analysis is mostly robust. I have one concern of statistical analyses performed in Figure 2D. In the SARS1 coronavirus panel of this figure, statistical analysis indicates that 10% PG causes significantly more infection (in RLU) than 0% PG. The same is true for the SARS-CoV-2 Alpha panel. The differences in infection level between 0% and 10% PG by eye are minimal, and authors should consider whether this statistical test is the most robust/appropriate, and whether these particular statistical differences are biologically meaningful to report. If analyses remain as is, authors should postulate why this small increase in infection level was observed for some lentivirus pseudotypes at 10% PG compared to controls.

OPTIONAL - The study could be further enhanced by preliminary mechanistic investigation into the action of propylene glycol (PG) - for example, authors postulate that PG may act via disruption of the lipid membrane, and this could be supported by EM imaging of a few PG-treated viruses. Alternatively, non-enveloped viruses could be tested for sensitivity to determine the importance of the envelope. Authors mention this in the discussion as being outside the scope, though testing one or two non-enveloped viruses would elevate the work substantially, as it opens the possibility for PG to also protect against non-enveloped respiratory viruses such as rhinovirus, as well as enteric viruses.

OPTIONAL - All influenza work in this study used the lab-adapted isolate A/PR8. This is a very useful and representative virus, though the study may benefit from comparison of the PG-

mediated inactivation kinetic of A/PR8 with that of a more recent clinical isolate of influenza A. This could be a useful supplementary figure.

Minor comments

Could authors generate (or reference) a robust correlation curve between IAV plaque area (px^2) that is calculated by the ColonyArea plugin, in relation to actual PFU/mL as counted by eye? This would help the reader to understand the decrease in viral titre that PG mediates from experiments conducted in the aerosol tunnel, e.g. does a decrease from 10^5 px^2 down to 10^3 px^2 equate to a 2-log10 reduction in PFU, or it is less than this?

This correlation could also be used to generate a detection limit for Plaque Area (px^2) that is equivalent to 1 or 10 PFU/well, which could then be added to graphs in Figure 3, S4, S5. This will help the reader understand if detection of 10^3 px^2 (for example) is quite high, or if it is already close to detection limits.

Can authors specify if the PEG was mixed with virus immediately prior to inoculation of animals, or was it mixed ahead of time (e.g. 30 mins or 1 hour ahead of administration)? In this case, low-level inactivation of viruses in bulk solution may have occurred. Figure 1C shows minimal inactivation should have occurred with 20% PG over this time-course at room-temperature, but this detail would be helpful to specify.

Figure legend of Fig 1. D) currently reads "... inoculated with 50mL..." , should this be 50uL?

2. Significance:

Significance (Required)

This study offers a robust proof of concept that PG can be applied to viral disinfection of a large range of enveloped viruses in a variety of settings (aerosol, fomites, in vivo, etc). This work now requires follow-on clinical studies to establish optimal and safe PG levels for viral inactivation in real-world settings, and comprehensive testing of safety risks from long term PG-exposure before this virucide can become clinically applied. This impact of this primary research could be considered broad and translational.

The audiences anticipated to be interested in these results are varied, and include those in virology fields, medical personnel, aerosol scientists, disinfection experts, and public health/ policy makers.

Field of expertise of reviewer: virology, animal infections, immunology, aerosol-borne influenza virus.

3. How much time do you estimate the authors will need to complete the suggested revisions:

Estimated time to Complete Revisions (Required)

(Decision Recommendation)

Between 1 and 3 months

4. *Review Commons* values the work of reviewers and encourages them to get credit for their work. Select 'Yes' below to register your reviewing activity at Web of Science Reviewer Recognition Service (formerly Publons); note that the content of your review will not be visible on Web of Science.

Web of Science Reviewer Recognition

Yes

We thank the reviewers for their detailed comments, constructive feedback and insightful suggestions to improve our research and manuscript. Our detailed responses are in bold.

Reviewer #1 (Evidence, reproducibility and clarity (Required)):

Summary

This paper by Styles et al describes the effect of propylene glycol (PG) on a range of enveloped viruses, focusing mainly on IAV and SARS-Cov2. The direct virucidal effect is shown both in vitro and in vivo, as well as the effect of vaporized PG on airborne IAV and SARS-Cov-2. This is a well-performed and very clear study.

Major comments

The claims and conclusions are in general well supported by the data, yet I have some comments.

- Interestingly, there were variations in the effect of PG on the different pseudoviruses. Surprising as I assumed that the effect of PG would be mainly on the lipid envelope, as you also mention in the discussion. It is suggested that PG therefore also has an effect on the conformation of the surface proteins. This can be easily tested, does the addition of PG result in a changed recognition by antibodies targeting these surface proteins? This information would be very valuable for your manuscript.

Many thanks for this excellent suggestion. To address this we are working in collaboration with Dr Joe Grove (Centre for Virus Research, University of Glasgow) using immobilised purified pseudovirus particles +/- PG treatment. Immunostaining against pseudovirus glycoproteins and their HIV gagpol capsid core enables comprehensive analysis of particle composition and integrity. Our preliminary experiments since submission have shown treatment with >60% PG increased gagpol signal, indicating compromised envelope integrity of equivalent magnitude to direct permeabilisation (positive control condition). At PG concentrations between 25 – 60% we've observed a significant reduction in glycoprotein signal preceding substantial envelope disruption. We are repeating these experiments in parallel with infectivity assays to assess how these changes in glycoprotein antibody recognition and envelope permeabilization relate to pseudovirus entry. We anticipate these studies will be completed within 8 weeks.

In addition, we are optimising cryo-EM protocols for analysis of SARS-CoV-2 virus particles +/- PG treatment to directly visualise the impact of PG on virus structure, in collaboration with Dr Paul Simpson (EM Centre, Imperial College).

- Figure S1 shows that although the clinical scores are better, the viral load is not reduced by the addition of PG to the inoculum. This is not mentioned in the main text. This seems very relevant information and should be presented in the main text. Can you please elaborate on this.

We agree that the virological and immunological findings are of interest, and have added the following to the main text *"Analysis of the remaining mice showed PG inhalation reduced clinical score and bronchioalveolar lavage cell counts on day 5, despite equivalent nasal and airway viral loads at this late time post-infection."* Examining longitudinal

weight loss and survival was the primary objective of this experimental design, which only allowed analysis of BAL cell counts and viral loads in the remaining mice at the experimental end point (day 5 post-infection). As 3 mice in the IAV only group reached the severity limit on day 3, we did not have the statistical power to detect significant differences in clinical scores, lung infiltrates or viral loads. IAV burden in the nose and lung usually reach peak levels at day 2/3 post-infection, and further experiments to look at the magnitude of viral replication and immunopathology over the course of infection in PG-treated mice will be important for future translational studies. As such, we have added the following to the Fig. S1 legend *"Further investigations are required to determine whether PG treatment reduces inflammatory cell infiltrates or peak IAV loads on days 2/3 post-infection."*

- Optional: It would be very interesting to see the effect of vaporized PG on viral transmission between mice/ferrets of a highly infectious virus (eg Sendai virus). This would give an idea if the PG also works on more biological respiratory droplets.

We wholeheartedly agree. To perform these additional studies requires additional funding and project licence amendments so unfortunately must lie beyond the scope of this study.

Minor comments

- The caption of Fig1D mentions that the mice are inoculated with 50ml total volume. I presume this should be µl.

Absolutely, thank you for spotting this error and it is now corrected.

- Please put the axis of the viral titer graph now in Fig s1 in PFU/lung or nasal cavity? This would be more informative.

Many thanks for this suggestion, now changed to PFU/lung along with BAL cells/lung.

- Typo in 'PG has broad-spectrum virucidal activity' paragraph: titre should be titer.

Titre is the British English spelling whereas titer is the American English spelling. We will comply with the journal specification on American versus British English in the final text.

- Is allergy to PG an issue that could have an effect on further development of this antiviral strategy?

A recent review (Pemberton & Kimber https://doi.org/10.1016/j.yrtph.2023.105341) of the evidence on this topic concludes "..*the weight of evidence points to PG possessing no, or only extremely modest, skin sensitising properties.*" There is anecdotal evidence of PG potentially acting as a very weak allergen in humans with underlying/pre-disposing skin conditions. Further clinical development of PG as a virucide would need to balance its significant benefits against this when "..*the risk for sensitisation to PG on uncompromised skin seems to be extremely low."*

Reviewer #1 (Significance (Required)):

This study is highly relevant. It proposes an easy and possibly efficacious method to prevent airborne transmission of a broad range of (enveloped) viruses.

A strength of this study is the set-up with the virus transmission tunnel. This nicely shows the effect of the vaporized PG on airborne IAV and SARS-Cov-2. A limitation is that the researchers did not analyze non-enveloped viruses, which should be done in a follow-up study.

Many thanks for these comments. Since submission we have also extended our investigations into (non-enveloped) rotaviruses in collaboration with Dr Alex Borodavka (Department of Biochemistry, University of Cambridge). Preliminary experiments have shown that rotavirus infectivity decreased from ~10^8 PFU/mL to undetectable levels (<10^2 PFU/mL) following incubation with 50% PG at 37°C for 1h compared to 0% PG control. We are now testing different temperatures, PG concentrations and incubation durations. We will include these new data in our revised submission.

There does not seem to be any recent research investigating this compound as an antiviral tool. Although the compound was tested for its antimicrobial properties in the 1940s, this has not been continued for unclear reasons (?).

We speculate the rapid development and clinical testing of conventional antibiotics during this period (culminating the Nobel prize for Medicine for Flemming, Florey and Chain in 1945) is responsible for the abrupt cessation of research into PG as an antimicrobial despite its obvious efficacy. We were unaware of these studies when we commenced our research into PG and this literature seems to have flown completely under the radar.

This is very valuable research in this world recovering from the recent SARS-Cov-2 pandemic. More mechanistic studies need to be performed to understand how this compound exerts its antiviral and antimicrobial effect.

A more specialized audience will be interested in this study.

My field of expertise: RSV, PIV, antivirals

Reviewer #2 (Evidence, reproducibility and clarity (Required)):

SUMMARY:
This study investigates the inactivation potential of propylene glycol against a number of important human respiratory viruses, including influenza A virus and SARS-CoV-2. Potent antiviral activity of propylene glycol was demonstrated for viruses in bulk solutions, in 2uL droplets on a variety of surfaces, and within aerosols. Additionally, propylene glycol was added to viruses prior to infection of live animals, and this treatment significantly improved animal survival outcomes and reduced their weight loss and clinical scores compared to animals infected with non-treated virus. Levels of propylene glycol utilised in the study were substantially less than those classified as well-tolerated by mammals. Overall, authors

demonstrate that propylene glycol is a strong yet safe virucide that could be used to control the spread of viral infections.

MAJOR COMMENTS:
Claims and conclusions are very well-supported by the data presented. Both the data presentation and method details are sufficient to reproduce the experiments. Authors might like to add precise gram concentrations of PG in addition to the percentages listed in the manuscript, either g/L or g/kg as appropriate. This would also allow easier comparison of the tested PG does to those published as safe doses for mammals, which are often given as g/kg.

Many thanks for this suggestion. These calculations and the concentration are now in a new "supplementary calculations" section in the supplementary material and the Figure 1 legend directs readers to them: ***"See supplementary materials for % PG solution (v/v) to g/L conversion and mouse PG dose in g/kg."***

Replicates are more than adequate, and statistical analysis is mostly robust. I have one concern of statistical analyses performed in Figure 2D. In the SARS1 coronavirus panel of this figure, statistical analysis indicates that 10% PG causes significantly more infection (in RLU) than 0% PG. The same is true for the SARS-CoV-2 Alpha panel. The differences in infection level between 0% and 10% PG by eye are minimal, and authors should consider whether this statistical test is the most robust/appropriate, and whether these particular statistical differences are biologically meaningful to report. If analyses remain as is, authors should postulate why this small increase in infection level was observed for some lentivirus pseudotypes at 10% PG compared to controls.

Many thanks to the reviewer for highlighting this. Whilst Dunnet's post-test we employed does indeed suggest a significant difference between 10% and 0% PG for some of the pseudoviruses, another post-test (Bonferroni's) does not. The reviewer correctly questions the biological significance of these very small differences in luciferase activity. In our experience, effect sizes of less than half a log unit are not biologically meaningful and certainly we make no claims that 10% PG is affecting pseudovirus entry in these experiments. To avoid any ambiguity therefore, we have removed the "*" from the relevant graphs and have restricted reporting of statistical significance to those conditions with $p<0.01$ vs the relevant controls.

OPTIONAL - The study could be further enhanced by preliminary mechanistic investigation into the action of propylene glycol (PG) - for example, authors postulate that PG may act via disruption of the lipid membrane, and this could be supported by EM imaging of a few PG-treated viruses. Alternatively, non-enveloped viruses could be tested for sensitivity to determine the importance of the envelope. Authors mention this in the discussion as being outside the scope, though testing one or two non-enveloped viruses would elevate the work substantially, as it opens the possibility for PG to also protect against non-enveloped respiratory viruses such as rhinovirus, as well as enteric viruses.

As explained in detail in our response to reviewer 1 and our revision plan, we are addressing these excellent suggestions in three ways for the revised manuscript:

1) **Collaboration with Dr Joe Grove's group to address PG's action on viral surface glycoproteins, virus composition and envelope integrity.**
2) **EM imaging of PG-treated SARS-CoV-2 with Dr Paul Simpson.**
3) **Collaboration with Dr Alex Borodavka's group to investigate PG-mediated virucidal activity against non-enveloped rotaviruses.**

OPTIONAL - All influenza work in this study used the lab-adapted isolate A/PR8. This is a very useful and representative virus, though the study may benefit from comparison of the PG-mediated inactivation kinetic of A/PR8 with that of a more recent clinical isolate of influenza A. This could be a useful supplementary figure.

Many thanks for this suggestion. We agree that examining the inactivation kinetics of different flu strains *in vitro* would be informative and will endeavour to include this in our revised manuscript if these experiments can be performed within an appropriate time frame. We know PG is effective against the 2009 pandemic strain H1N1 A/California/7/2009 as this was used for our *in vivo* experiments as listed in the methods section. For clarity we have added this information into the main text.

MINOR COMMENTS:

Could authors generate (or reference) a robust correlation curve between IAV plaque area (px^2) that is calculated by the ColonyArea plugin, in relation to actual PFU/mL as counted by eye? This would help the reader to understand the decrease in viral titre that PG mediates from experiments conducted in the aerosol tunnel, e.g. does a decrease from 10^5 px^2 down to 10^3 px^2 equate to a 2-log10 reduction in PFU, or it is less than this?

This correlation could also be used to generate a detection limit for Plaque Area (px^2) that is equivalent to 1 or 10 PFU/well, which could then be added to graphs in Figure 3, S4, S5. This will help the reader understand if detection of 10^3 px^2 (for example) is quite high, or if it is already close to detection limits.

The reviewer makes an interesting suggestion. As can be seen from the requested representation of those data (reviewer figure 1, replotted from Fig 3D), for lower nebulised input virus amounts where infection did not transmit beyond the first plate there is a very respectable linear correlation between plaque number and plaque area (R^2=0.72, Spearman's r=0.98, p<0.0001). This demonstrates that the plaque area detection limit of e.g. 10^2 – 10^3 px^2 reflects the 1 – 10 plaque detection limit. However, when performing initial controls we found this relationship soon departs from linearity as input virus amount increases; essentially plaques begin to overlap with each other on the plates closest to the nebuliser. We are concerned it might be inappropriate to present PFU/well extrapolated from plaque areas collected from lower input virus experiments, since this could potentially mislead or confuse the reader about the fidelity and nature of the transmission tunnel experimental system. Moreover, for this particular type of assay plaque area affords a much greater dynamic range than PFU/well, which is essential for

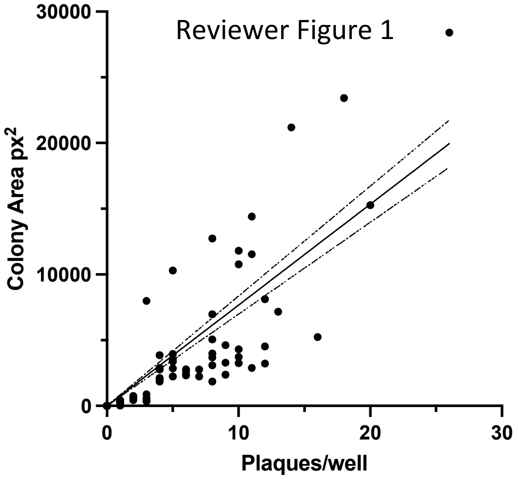

demonstrating the range of PG's virucidal activity with higher input virus, range of vapor concentrations and over different transmission distances. Taking these considerations into account, and unavoidable idiosyncrasies of testing virus transmission under more "natural" conditions (e.g. direct virus deposition on cell monolayers prohibiting serial dilution for exact PFU determination), we have decided to continue reporting plaque area only for quantification of transmission tunnel data - the parameter that was actually measured. This accurately reflects the extensive cell clearance under control conditions, where the monolayer is completely disrupted, compared to the protective effect of PG vapor, where the monolayer is largely intact with limited plaques. We hope the reviewer understands our reasoning.

To communicate this, we have added the following sentence to the methods section: "Plaque area shows a strong linear correlation with plaque number at lower viral inputs (R^2=0.72, Spearman's r=0.98, p<0.0001), that becomes non-linear at higher titres as plaques begin to overlap. To encompass the broadest dynamic range therefore, plaque area is reported for transmission tunnel data."

Can authors specify if the PEG was mixed with virus immediately prior to inoculation of animals, or was it mixed ahead of time (e.g. 30 mins or 1 hour ahead of administration)? In this case, low-level inactivation of viruses in bulk solution may have occurred. Figure 1C shows minimal inactivation should have occurred with 20% PG over this time-course at room-temperature, but this detail would be helpful to specify.

Many thanks for bringing this oversight to our attention. IAV was mixed with PG immediately prior to inoculation of animals (<1 minute) such that negligible inactivation occurs *in vitro* as shown in Fig S5D: when IAV is combined with PG then diluted for plaque assay after 1 minute the viral titre shows no significant change. We have clarified this in the revised manuscript by stating *"Mice were inoculated immediately after IAV and PG were combined (<1 min), with negligible PG-mediated inactivation prior to infection (Fig. S5D)."*

Figure legend of Fig 1. D) currently reads "... inoculated with 50mL..." , should this be 50uL?

It should, thank you for spotting this error and it is now corrected.

Reviewer #2 (Significance (Required)):

This study offers a robust proof of concept that PG can be applied to viral disinfection of a large range of enveloped viruses in a variety of settings (aerosol, fomites, in vivo, etc). This work now requires follow-on clinical studies to establish optimal and safe PG levels for viral inactivation in real-world settings, and comprehensive testing of safety risks from long term PG-exposure before this virucide can become clinically applied. This impact of this primary research could be considered broad and translational.

The audiences anticipated to be interested in these results are varied, and include those in virology fields, medical personnel, aerosol scientists, disinfection experts, and public health/ policy makers.

Field of expertise of reviewer: virology, animal infections, immunology, aerosol-borne influenza virus.

3rd May 2023

Dear Dr. Edgar,

Thank you for the submission of your research manuscript to our editorial offices. I have now had the opportunity to read the manuscript, the referee reports, revision plan and your point-by-point response and to discuss it with the other members of our editorial team. We all agreed that the manuscript fits the scope of EMBO Molecular Medicine but also that the referees raised important concerns that should be addressed in major revision. In our opinion your revision plan addresses the referees' criticism adequately and therefore we would like to invite submission of the revised manuscript to our journal.

Further consideration of a revision that addresses reviewers' concerns in full will entail a second round of review. EMBO Molecular Medicine encourages a single round of revision only and therefore, acceptance or rejection of the manuscript will depend on the completeness of your responses included in the next, final version of the manuscript. For this reason, and to save you from any frustrations in the end, I would strongly advise against returning an incomplete revision.

We would welcome the submission of a revised version within three months for further consideration. Please let us know if you require longer to complete the revision.

I look forward to receiving your revised manuscript.

Yours sincerely,

Zeljko Durdevic

Zeljko Durdevic
Editor
EMBO Molecular Medicine

*****

When submitting your revised manuscript, please carefully review the instructions that follow below. We perform an initial quality control of all revised manuscripts before re-review; failure to include requested items will delay the evaluation of your revision.

We require:

1) A .docx formatted version of the manuscript text (including legends for main figures, EV figures and tables). Please make sure that the changes are highlighted to be clearly visible.

2) Individual production quality figure files as .eps, .tif, .jpg (one file per figure). For guidance, download the 'Figure Guide PDF': (https://www.embopress.org/page/journal/17574684/authorguide#figureformat).

3) A .docx formatted letter INCLUDING the reviewers' reports and your detailed point-by-point responses to their comments. As part of the EMBO Press transparent editorial process, the point-by-point response is part of the Review Process File (RPF), which will be published alongside your paper.

4) A complete author checklist, which you can download from our author guidelines (https://www.embopress.org/page/journal/17574684/authorguide#submissionofrevisions). Please insert information in the checklist that is also reflected in the manuscript. The completed author checklist will also be part of the RPF.

5) Please note that all corresponding authors are required to supply an ORCID ID for their name upon submission of a revised manuscript.

6) It is mandatory to include a 'Data Availability' section after the Materials and Methods. Before submitting your revision, primary

datasets produced in this study need to be deposited in an appropriate public database, and the accession numbers and database listed under 'Data Availability'. Please remember to provide a reviewer password if the datasets are not yet public (see https://www.embopress.org/page/journal/17574684/authorguide#dataavailability).

In case you have no data that requires deposition in a public database, please state so in this section. Note that the Data Availability Section is restricted to new primary data that are part of this study.

7) For data quantification: please specify the name of the statistical test used to generate error bars and P values, the number (n) of independent experiments (specify technical or biological replicates) underlying each data point and the test used to calculate p-values in each figure legend. The figure legends should contain a basic description of n, P and the test applied. Graphs must include a description of the bars and the error bars (s.d., s.e.m.). See also 'Figure Legend' guidelines: https://www.embopress.org/page/journal/17574684/authorguide#figureformat

8) At EMBO Press we ask authors to provide source data for the main manuscript figures. Our source data coordinator will contact you to discuss which figure panels we would need source data for and will also provide you with helpful tips on how to upload and organize the files.

9) Our journal encourages inclusion of *data citations in the reference list* to directly cite datasets that were re-used and obtained from public databases. Data citations in the article text are distinct from normal bibliographical citations and should directly link to the database records from which the data can be accessed. In the main text, data citations are formatted as follows: "Data ref: Smith et al, 2001" or "Data ref: NCBI Sequence Read Archive PRJNA342805, 2017". In the Reference list, data citations must be labeled with "[DATASET]". A data reference must provide the database name, accession number/identifiers and a resolvable link to the landing page from which the data can be accessed at the end of the reference. Further instructions are available at .

10) We replaced Supplementary Information with Expanded View (EV) Figures and Tables that are collapsible/expandable online. A maximum of 5 EV Figures can be typeset. EV Figures should be cited as 'Figure EV1, Figure EV2" etc... in the text and their respective legends should be included in the main text after the legends of regular figures.

- For the figures that you do NOT wish to display as Expanded View figures, they should be bundled together with their legends in a single PDF file called *Appendix*, which should start with a short Table of Content. Appendix figures should be referred to in the main text as: "Appendix Figure S1, Appendix Figure S2" etc.

- Additional Tables/Datasets should be labeled and referred to as Table EV1, Dataset EV1, etc. Legends have to be provided in a separate tab in case of .xls files. Alternatively, the legend can be supplied as a separate text file (README) and zipped together with the Table/Dataset file.

See detailed instructions here:

.

11) The paper explained: EMBO Molecular Medicine articles are accompanied by a summary of the articles to emphasize the major findings in the paper and their medical implications for the non-specialist reader. Please provide a draft summary of your article highlighting

- the medical issue you are addressing,

- the results obtained and

- their clinical impact.

This may be edited to ensure that readers understand the significance and context of the research. Please refer to any of our published articles for an example.

12) For more information: There is space at the end of each article to list relevant web links for further consultation by our readers. Could you identify some relevant ones and provide such information as well? Some examples are patient associations, relevant databases, OMIM/proteins/genes links, author's websites, etc...

13) Author contributions: the contribution of every author must be detailed in a separate section (before the acknowledgments).

14) A Conflict of Interest statement should be provided in the main text.

15) Every published paper now includes a 'Synopsis' to further enhance discoverability. Synopses are displayed on the journal

webpage and are freely accessible to all readers. They include a short stand first (maximum of 300 characters, including space) as well as 2-5 one-sentences bullet points that summarizes the paper. Please write the bullet points to summarize the key NEW findings. They should be designed to be complementary to the abstract - i.e. not repeat the same text. We encourage inclusion of key acronyms and quantitative information (maximum of 30 words / bullet point). Please use the passive voice. Please attach these in a separate file or send them by email, we will incorporate them accordingly.

Please also suggest a striking image or visual abstract to illustrate your article as a PNG file 550 px wide x 300-600 px high.

EMBO Molecular Medicine has a "scooping protection" policy, whereby similar findings that are published by others during review or revision are not a criterion for rejection. Should you decide to submit a revised version, I do ask that you get in touch after three months if you have not completed it, to update us on the status.

***

Rev_Com_number: RC-2023-01877
New_manu_number: EMM-2023-17932
Corr_author: Edgar
Title: Propylene glycol inactivates respiratory viruses and prevents airborne transmission

---

We thank the reviewers for their detailed comments, constructive feedback and insightful suggestions to improve our research & manuscript. Our detailed responses are in bold.

Reviewer #1 (Evidence, reproducibility and clarity (Required)):

Summary
This paper by Styles et al describes the effect of propylene glycol (PG) on a range of enveloped viruses, focusing mainly on IAV and SARS-Cov2. The direct virucidal effect is shown both in vitro and in vivo, as well as the effect of vaporized PG on airborne IAV and SARS-Cov-2. This is a well-performed and very clear study.

Major comments
The claims and conclusions are in general well supported by the data, yet I have some comments.

- Interestingly, there were variations in the effect of PG on the different pseudoviruses. Surprising as I assumed that the effect of PG would be mainly on the lipid envelope, as you also mention in the discussion. It is suggested that PG therefore also has an effect on the conformation of the surface proteins. This can be easily tested, does the addition of PG result in a changed recognition by antibodies targeting these surface proteins? This information would be very valuable for your manuscript.

Many thanks for this excellent suggestion. We addressed this in collaboration with Dr Grove's laboratory (Centre for Virus Research, University of Glasgow) using immobilised purified SARS-CoV-2 and VSV pseudovirus particles +/- PG treatment. Immunostaining against pseudovirus glycoproteins and their HIV gagpol capsid core enables comprehensive analysis of particle composition and integrity. Treatment with >60% PG increased gagpol signal, indicating compromised envelope integrity, and at

75% (v/v) PG we observe membrane permeabilization of equivalent magnitude to treatment with the detergent NP-40. We observed no consistent changes in glycoprotein immunostaining upon PG treatment with the antibodies used in this study. However, the concentration of PG required to significantly disrupt the pseudovirus envelope was much higher than that required to abolish SARS-CoV-2 and VSV pseudovirus infectivity (>60% versus 40% v/v PG), indicating that PG-mediated particle inactivation is not solely due to membrane effects. These findings are presented in revised Figure 3A-C, with additions to the text as follows:

"One way that PG could restrict infection is by disrupting the viral phospholipid envelope, as shown for bacterial membranes (Rubiano *et al*, 2020)***. To assess this, we employed a lentivirus-based pseudovirus assay whereby concentrated SARS-CoV-2 (Wuhan strain) pseudovirus was immobilised onto coverslips and then incubated with PG before staining with anti-capsid and anti-glycoprotein antibodies, followed by fluorescent secondary antibodies (Figure 3A). Signal intensity in the capsid and glycoprotein channels was assessed to determine envelope and glycoprotein integrity, with a higher capsid signal indicative of membrane permeabilization. A significant loss of SARS-CoV-2 pseudovirus envelope integrity occurs between 65 – 75% PG treatment, with capsid antibody binding at 75% PG solution equivalent to the NP-40 detergent control (Figure 3B, C). The same pattern was observed for an alternative pseudovirus containing glycoprotein from vesicular stomatitis virus (VSV-G; Figure EV4A).***

SARS-CoV-2 Wuhan strain pseudovirus infectivity is restricted at PG concentrations above 20% (v/v) and abolished with 40% PG (Figure 2D), much lower than the PG concentrations required for significant permeabilization of the viral envelope. Whilst PG-mediated membrane disruption likely contributes to pseudovirus inactivation, direct effects on viral proteins cannot be discounted, although we observed no consistent difference in glycoprotein signal upon PG treatment with the antibodies used in this study (Figure 3B, C; Figure EV4A)."

- Figure S1 shows that although the clinical scores are better, the viral load is not reduced by the addition of PG to the inoculum. This is not mentioned in the main text. This seems very relevant information and should be presented in the main text. Can you please elaborate on this.

We agree that the virological and immunological findings are of interest, and have added Figure EV2 and the following to the main text:
"Analysis of the remaining mice on day 5 post-infection showed PG inhalation reduced clinical score and bronchioalveolar lavage cell counts, despite equivalent nasal and airway viral loads at this late time post-infection (Figure EV2). Although further investigations are required to determine whether PG treatment reduces peak inflammatory cell infiltrates and viral loads at earlier times post-infection, we conclude that PG can safely reduce the infectivity of influenza A virus in vitro and in vivo."

Examining longitudinal weight loss and survival was the primary objective of this experimental design, which only allowed analysis of BAL cell counts and viral loads in the remaining mice at the experimental end point (day 5 post-infection). As 3 mice in the IAV only group reached the severity limit on day 3, we did not have the statistical power to detect significant differences in clinical scores, lung infiltrates or viral loads. IAV burden in the nose and lung usually reach peak levels at day 2/3 post-infection, and further experiments to look at the magnitude of viral replication and immunopathology over the course of infection in PG-treated mice will be important for future translational studies.

- Optional: It would be very interesting to see the effect of vaporized PG on viral transmission between mice/ferrets of a highly infectious virus (eg Sendai virus). This would give an idea if the PG also works on more biological respiratory droplets.

We wholeheartedly agree. These experiments require substantial additional funding and project licence amendments so unfortunately lie beyond the scope of this current study.

Minor comments

- The caption of Fig1D mentions that the mice are inoculated with 50ml total volume. I presume this should be µl.

Thank you for spotting this error and it is now corrected.

- Please put the axis of the viral titer graph now in Fig s1 in PFU/lung or nasal cavity? This would be more informative.

Many thanks for this suggestion, now changed to PFU/lung or PFU/nasal lavage, along with BAL cells/lung.

- Typo in 'PG has broad-spectrum virucidal activity' paragraph: titre should be titer.

Titre is the British English spelling whereas titer is the American English spelling. We had inconsistently used British and American English throughout the document and have now converted all text to British English spelling (e.g. vapour rather than vapor). We will comply with the journal specification on American versus British English in the final text.

- Is allergy to PG an issue that could have an effect on further development of this antiviral strategy?

A recent review (Pemberton & Kimber https://doi.org/10.1016/j.yrtph.2023.105341) of the evidence on this topic concludes "..the weight of evidence points to PG possessing no, or only extremely modest, skin sensitising properties." There is anecdotal evidence of PG potentially acting as a very weak allergen in humans with underlying/pre-disposing skin conditions. Further clinical development of PG as a virucide would need to balance its significant benefits against this when "..the risk for sensitisation to PG on uncompromised skin seems to be extremely low."

Reviewer #1 (Significance (Required)):

This study is highly relevant. It proposes an easy and possibly efficacious method to prevent airborne transmission of a broad range of (enveloped) viruses. A strength of this study is the set-up with the virus transmission tunnel. This nicely shows the effect of the vaporized PG on airborne IAV and SARS-Cov-2. A limitation is that the researchers did not analyze non-enveloped viruses, which should be done in a follow-up study.

Many thanks for these comments. We have extended our investigations to non-enveloped rotaviruses in collaboration with Dr Borodavka's laboratory (Department of Biochemistry, University of Cambridge). PG reduces rotavirus infectivity in a time-, temperature- and concentration-dependent manner and these results are presented in Figure 3D, with additions to the main text as follows:

Therefore, we assessed whether PG treatment could reduce the infectivity of non-enveloped rotavirus (RV), the causative agent of severe gastroenteritis in children (Crawford et al, 2017). PG could exert a virucidal effect against non-enveloped viruses by altering the structure of surface proteins that mediate host membrane penetration or by direct capsid disruption (Kumar et al, 2018). Incubation of the simian RV strain SA11 with PG resulted in time-, temperature- and concentration-dependent virus inactivation (Figure 3D; Figure EV4B), with similar kinetics to enveloped IAV and EBV (Figure 1A-F, 2B; Figure EV1, EV3B). This indicates that PG can interfere with viral protein structure to render RV and potentially other non-enveloped viruses non-infectious.

There does not seem to be any recent research investigating this compound as an antiviral tool. Although the compound was tested for its antimicrobial properties in the 1940s, this has not been continued for unclear reasons (?).

We speculate the rapid development and clinical testing of conventional antibiotics during this period is responsible for the abrupt cessation of research into PG as an antimicrobial, despite its obvious efficacy. We were unaware of these studies when we commenced our research into PG and this literature seems to have flown completely under the radar.

This is very valuable research in this world recovering from the recent SARS-Cov-2 pandemic. More mechanistic studies need to be performed to understand how this compound exerts its antiviral and antimicrobial effect.

A more specialized audience will be interested in this study.

My field of expertise: RSV, PIV, antivirals

Reviewer #2 (Evidence, reproducibility and clarity (Required)):

SUMMARY:
This study investigates the inactivation potential of propylene glycol against a number of important human respiratory viruses, including influenza A virus and SARS-CoV-2. Potent antiviral activity of propylene glycol was demonstrated for viruses in bulk solutions, in 2uL droplets on a variety of surfaces, and within aerosols. Additionally, propylene glycol was added to viruses prior to infection of live animals, and this treatment significantly improved animal survival outcomes and reduced their weight loss and clinical scores compared to animals infected with non-treated virus. Levels of propylene glycol utilised in the study were substantially less than those classified as well-tolerated by mammals. Overall, authors demonstrate that propylene glycol is a strong yet safe virucide that could be used to control the spread of viral infections.

MAJOR COMMENTS:
Claims and conclusions are very well-supported by the data presented. Both the data presentation and method details are sufficient to reproduce the experiments. Authors might like to add precise gram concentrations of PG in addition to the percentages listed in the manuscript, either g/L or g/kg as appropriate. This would also allow easier comparison of the tested PG does to those published as safe doses for mammals, which are often given as g/kg.

Many thanks for this suggestion. The calculations for conversion of % PG (v/v) to both g/L and g/kg mouse weight are now in Appendix Table 1 and we have added the following text to the Figure 1 legend:
"For % PG (v/v) conversion to g/L or g/kg see Appendix Table 1."

Replicates are more than adequate, and statistical analysis is mostly robust. I have one concern of statistical analyses performed in Figure 2D. In the SARS1 coronavirus panel of this figure, statistical analysis indicates that 10% PG causes significantly more infection (in RLU) than 0% PG. The same is true for the SARS-CoV-2 Alpha panel. The differences in infection level between 0% and 10% PG by eye are minimal, and authors should consider whether this statistical test is the most robust/appropriate, and whether these particular statistical differences are biologically meaningful to report. If analyses remain as is, authors should postulate why this small increase in infection level was observed for some lentivirus pseudotypes at 10% PG compared to controls.

We agree that effect sizes of less than half a log are not biologically meaningful and certainly we make no claims that 10% PG is affecting pseudovirus entry in these experiments. We realised that we had erroneously performed our ANOVA analyses on raw data that does not conform to the normal distribution assumed by these tests, rather than on log_{10}-transformed data. Many thanks to the reviewer for highlighting this error, which is now corrected. To make this clear, we have added the following text to the Statistical Analysis methods section:
"Statistical tests for virus assays were performed on log_{10}-transformed data ($Y=log_{10}[Y+1]$)."

OPTIONAL - The study could be further enhanced by preliminary mechanistic investigation into the action of propylene glycol (PG) - for example, authors postulate that PG may act via disruption of the lipid membrane, and this could be supported by EM imaging of a few PG-treated viruses. Alternatively, non-enveloped viruses could be tested for sensitivity to determine the importance of the envelope. Authors mention this in the discussion as being outside the scope, though testing one or two non-enveloped viruses would elevate the work substantially, as it opens the possibility for PG to also protect against non-enveloped respiratory viruses such as rhinovirus, as well as enteric viruses.

As explained in detail in our responses above, we have explored the mechanistic basis for PG virucidal activity using immunostaining of immobilised pseudoviruses to examine particle integrity, and by directly testing PG efficacy against non-enveloped rotavirus.

OPTIONAL - All influenza work in this study used the lab-adapted isolate A/PR8. This is a very useful and representative virus, though the study may benefit from comparison of the PG-mediated inactivation kinetic of A/PR8 with that of a more recent clinical isolate of influenza A. This could be a useful supplementary figure.

Many thanks for this suggestion. We know PG is effective against the 2009 pandemic strain H1N1 A/California/7/2009 as this was used for our *in vivo* experiments as listed in the methods section. For clarity we have added this information into the main text. In addition, we have further tested the 2009 pandemic clinical isolate H1N1 A/England/195/09 *in vitro*. PG inactivates this E195 IAV with similar kinetics to PR8. This new data is presented in Figure 1, EV1 and additional text:
"These results were mirrored with the 2009 influenza pandemic clinical isolate H1N1 A/England/195/09 ([E195)]; Figure 1D-F, Figure EV1D-F)."

MINOR COMMENTS:

Could authors generate (or reference) a robust correlation curve between IAV plaque area (px^2) that is calculated by the ColonyArea plugin, in relation to actual PFU/mL as counted by eye? This would help the reader to understand the decrease in viral titre that PG mediates from experiments conducted in the aerosol tunnel, e.g. does a decrease from 10^5 px^2 down to 10^3 px^2 equate to a 2-log10 reduction in PFU, or it is less than this?
This correlation could also be used to generate a detection limit for Plaque Area (px^2) that is equivalent to 1 or 10 PFU/well, which could then be added to graphs in Figure 3, S4, S5. This will help the reader understand if detection of 10^3 px^2 (for example) is quite high, or if it is already close to detection limits.

The reviewer makes an interesting suggestion. As can be seen from the requested representation of those data (reviewer figure 1, replotted from Fig 3D), for lower nebulised input virus amounts where infection did not transmit beyond the first plate there is a very respectable linear correlation between plaque number and plaque area (R^2=0.72, Spearman's r=0.98, p<0.0001). This demonstrates that the plaque area detection limit of e.g. 10^2 – 10^3 px^2 reflects the 1 – 10 plaque detection limit. However, when performing initial controls we found this relationship soon departs from linearity as input virus amount increases; essentially plaques begin to overlap with each other on the plates closest to the nebuliser. We are concerned it might be inappropriate to present PFU/well extrapolated from plaque areas collected from lower input virus experiments, since this could potentially mislead or confuse the reader about the fidelity and nature of the transmission tunnel experimental system. Moreover, for this particular type of assay plaque area affords a much greater dynamic range than PFU/well, which is essential for demonstrating the range of PG's virucidal activity with higher input virus, range of vapour concentrations and over different transmission distances. Taking these considerations into account, and unavoidable idiosyncrasies of testing virus transmission under more "natural" conditions (e.g. direct virus deposition on cell monolayers prohibiting serial dilution for exact PFU determination), we have decided to continue reporting plaque area only for quantification of transmission tunnel data - the parameter that was actually measured. This accurately reflects the extensive cell clearance under control conditions, where the monolayer is completely disrupted, compared to the protective effect of PG vapour, where the monolayer is largely intact with limited plaques. We hope the reviewer understands our reasoning.

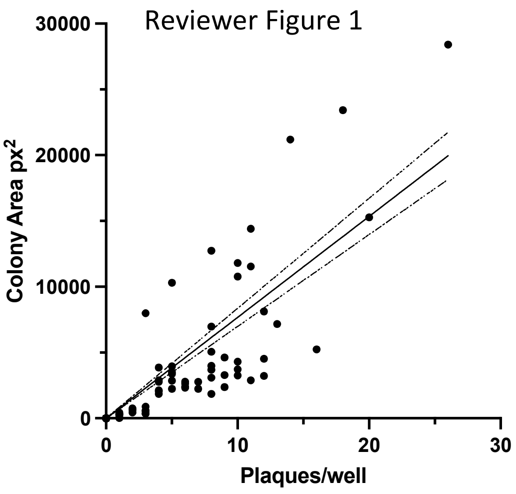

To communicate this, we have added the following sentence to the methods section:
"Plaque area shows a strong linear correlation with plaque number at lower viral inputs (R^2=0.72, Spearman's r=0.98, p<0.0001), that becomes non-linear at higher titres as plaques begin to overlap. To encompass the broadest dynamic range therefore, plaque area is reported for transmission tunnel data."

Can authors specify if the PEG was mixed with virus immediately prior to inoculation of animals, or was it mixed ahead of time (e.g. 30 mins or 1 hour ahead of administration)? In this case, low-level inactivation of viruses in bulk solution may have occurred. Figure 1C shows minimal inactivation should have occurred with 20% PG over this time-course at room-temperature, but this detail would be helpful to specify.

Many thanks for bringing this oversight to our attention. IAV was mixed with PG immediately prior to inoculation of animals (<1 minute) such that negligible inactivation occurs *in vitro* as shown in Figure EV4D: when IAV is combined with PG then diluted for plaque assay after 1 minute the viral titre shows no significant change. We have clarified this in the revised manuscript by stating
"Mice were inoculated immediately after IAV and PG were combined (<1 min), with negligible PG-mediated inactivation prior to infection (Figure EV5D)."

Figure legend of Fig 1. D) currently reads "... inoculated with 50mL..." , should this be 50uL?

It should, thank you for spotting this error and it is now corrected.

Reviewer #2 (Significance (Required)):

This study offers a robust proof of concept that PG can be applied to viral disinfection of a large range of enveloped viruses in a variety of settings (aerosol, fomites, in vivo, etc). This work now requires follow-on clinical studies to establish optimal and safe PG levels for viral inactivation in real-world settings, and comprehensive testing of safety risks from long term PG-exposure before this virucide can become clinically applied. This impact of this primary research could be considered broad and translational.

The audiences anticipated to be interested in these results are varied, and include those in virology fields, medical personnel, aerosol scientists, disinfection experts, and public health/ policy makers.

Field of expertise of reviewer: virology, animal infections, immunology, aerosol-borne influenza virus.

18th Sep 2023

Dear Dr. Edgar,

Thank you for the submission of your revised manuscript to EMBO Molecular Medicine. I am pleased to inform you that we will be able to accept your manuscript pending the following final amendments:

1) Please address the referee #1 minor comment.
2) In the main manuscript file, please do the following:
- Add up to 5 keywords.
- Remove "One-sentence summary" on the title page.
- Please callout all individual panels for figures EV2-4.
- In M&M, please specify the biosafety level for the experiments with viruses by adding and amending the following sentence: All experiments with viruses were performed in a ... level laboratory and with approval from...
- In M&M, add the following paragraph:

Graphics:
(some of the... OR Figure #... OR synopsis) Graphics were created with BioRender.com.

Please remove the reference to BioRender from figure legends.
- In M&M, statistical paragraph should reflect all information that you have filled in the Authors Checklist, especially regarding randomization, blinding, replication.
- Remove "List of Appendix material".
- Please rename "Conflict of interest statement" to "Disclosure Statement & Competing Interests". We updated our journal's competing interests policy in January 2022 and request authors to consider both actual and perceived competing interests. Please review the policy https://www.embopress.org/competing-interests and update your competing interests if necessary.
- Author contributions: Please remove it from the manuscript and specify author contributions in our submission system. CRediT has replaced the traditional author contributions section because it offers a systematic machine-readable author contributions format that allows for more effective research assessment. You are encouraged to use the free text boxes beneath each contributing author's name to add specific details on the author's contribution. More information is available in our guide to authors:
https://www.embopress.org/page/journal/17574684/authorguide#authorshipguidelines
- Place "Data availability" after M&M and replace the current text with following sentence: This study includes no data deposited in external repositories.
3) Funding: Please merge it with "Acknowledgments" and make sure that information about all sources of funding are complete in both our submission system and in the manuscript. Currently Royal Society of Chemistry (RSC) is missing in the manuscript.
4) Appendix: Please add page numbers and correct nomenclature to "Appendix Figure S1" etc and "Appendix Table S1" etc, also in the main manuscript text.
5) The Paper Explained: Please add it to the main manuscript file.
6) Synopsis:
- Synopsis image: Please provide a striking image or visual abstract as a high-resolution jpeg file 550 px-wide x (250-400)-px high to illustrate your article.
- Synopsis text: Please upload it as a separate .doc file.
- Please check your synopsis text and image before submission with your revised manuscript. Please be aware that in the proof stage minor corrections only are allowed (e.g., typos).
7) For more information: This space should be used to list relevant web links for further consultation by our readers. Could you identify some relevant ones and provide such information as well? Some examples are patient associations, relevant databases, OMIM/proteins/genes links, author's websites, etc...
8) As part of the EMBO Publications transparent editorial process initiative (see our Editorial at http://embomolmed.embopress.org/content/2/9/329), EMBO Molecular Medicine will publish online a Review Process File (RPF) to accompany accepted manuscripts. This file will be published in conjunction with your paper and will include the anonymous referee reports, your point-by-point response and all pertinent correspondence relating to the manuscript. Let us know whether you agree with the publication of the RPF and as here, if you want to remove or not any figures from it prior to publication. Please note that the Authors checklist will be published at the end of the RPF.
9) Please provide a point-by-point letter INCLUDING my comments as well as the reviewer's reports and your detailed responses (as Word file).

I look forward to reading a new revised version of your manuscript as soon as possible.

Yours sincerely,

Zeljko Durdevic

Zeljko Durdevic
Editor
EMBO Molecular Medicine

*** Instructions to submit your revised manuscript ***

*** PLEASE NOTE *** As part of the EMBO Publications transparent editorial process initiative (see our Editorial at https://www.embopress.org/doi/pdf/10.1002/emmm.201000094), EMBO Molecular Medicine will publish online a Review Process File to accompany accepted manuscripts.

In the event of acceptance, this file will be published in conjunction with your paper and will include the anonymous referee reports, your point-by-point response and all pertinent correspondence relating to the manuscript. If you do NOT want this file to be published, please inform the editorial office at contact@embomolmed.org.

When submitting your revised manuscript, please include:

1) a .docx formatted version of the manuscript text (including Figure legends and tables)

2) Separate figure files*

3) supplemental information as Expanded View and/or Appendix. Please carefully check the authors guidelines for formatting Expanded view and Appendix figures and tables at https://www.embopress.org/page/journal/17574684/authorguide#expandedview

4) a letter INCLUDING the reviewer's reports and your detailed responses to their comments (as Word file).

5) The paper explained: EMBO Molecular Medicine articles are accompanied by a summary of the articles to emphasize the major findings in the paper and their medical implications for the non-specialist reader. Please provide a draft summary of your article highlighting
- the medical issue you are addressing,
- the results obtained and
- their clinical impact.
This may be edited to ensure that readers understand the significance and context of the research.
Please refer to any of our published articles for an example.

6) For more information: There is space at the end of each article to list relevant web links for further consultation by our readers. Could you identify some relevant ones and provide such information as well? Some examples are patient associations, relevant databases, OMIM/proteins/genes links, author's websites, etc...

7) Author contributions: the contribution of every author must be detailed in a separate section.

8) EMBO Molecular Medicine now requires a complete author checklist (https://www.embopress.org/page/journal/17574684/authorguide) to be submitted with all revised manuscripts. Please use the checklist as guideline for the sort of information we need WITHIN the manuscript. The checklist should only be filled with page numbers were the information can be found. This is particularly important for animal reporting, antibody dilutions (missing) and exact values and n that should be indicted instead of a range.

9) Every published paper now includes a 'Synopsis' to further enhance discoverability. Synopses are displayed on the journal webpage and are freely accessible to all readers. They include a short stand first (maximum of 300 characters, including space) as well as 2-5 one sentence bullet points that summarise the paper. Please write the bullet points to summarise the key NEW findings. They should be designed to be complementary to the abstract - i.e. not repeat the same text. We encourage inclusion of key acronyms and quantitative information (maximum of 30 words / bullet point). Please use the passive voice. Please attach

these in a separate file or send them by email, we will incorporate them accordingly.

You are also welcome to suggest a striking image or visual abstract to illustrate your article. If you do please provide a jpeg file 550 px-wide x 400-px high.

10) A Conflict of Interest statement should be provided in the main text

11) Please note that we now mandate that all corresponding authors list an ORCID digital identifier. This takes <90 seconds to complete. We encourage all authors to supply an ORCID identifier, which will be linked to their name for unambiguous name identification.

Currently, our records indicate that the ORCID for your account is 0000-0002-3348-0851.

Please click the link below to modify this ORCID:
Link Not Available

12) The system will prompt you to fill in your funding and payment information. This will allow Wiley to send you a quote for the article processing charge (APC) in case of acceptance. This quote takes into account any reduction or fee waivers that you may be eligible for. Authors do not need to pay any fees before their manuscript is accepted and transferred to our publisher.

*Additional important information regarding Figures

Each figure should be given in a separate file and should have the following resolution:
Graphs 800-1,200 DPI
Photos 400-800 DPI
Colour (only CMYK) 300-400 DPI"

Figures are not edited by the production team. All lettering should be the same size and style; figure panels should be indicated by capital letters (A, B, C etc). Gridlines are not allowed except for log plots. Figures should be numbered in the order of their appearance in the text with Arabic numerals. Each Figure must have a separate legend and a caption is needed for each panel.

*Additional important information regarding figures and illustrations can be found at https://bit.ly/EMBOPressFigurePreparationGuideline. See also figure legend preparation guidelines: https://www.embopress.org/page/journal/17574684/authorguide#figureformat

***** Reviewer's comments *****

Referee #1 (Remarks for Author):

Thank you for adding these extra data to your manuscript, it has increased its quality consideraby.

I have one minor comment still. You show PFU/nasal lavage and PFU/lung in figure EV2D and E, but these values seem very low, around 20 PFU per lung at 5 DPI while the mice were inoculated with 5*10^4 PFU. I assume a multiplication of your values was forgotten somewhere in the analysis of the plaque assay.

Referee #2 (Comments on Novelty/Model System for Author):

Authors have suitably addressed all reviewer suggestions/comments.

***
Rev_Com_number: RC-2023-01877
New_manu_number: EMM-2023-17932-V2
Corr_author: Edgar
Title: Propylene glycol inactivates respiratory viruses and prevents airborne transmission

1) Please address the referee #1 minor comment.
Reviewers comments:
Referee #1 (Remarks for Author):

Thank you for adding these extra data to your manuscript, it has increased its quality considerably.

I have one minor comment still. You show PFU/nasal lavage and PFU/lung in figure EV2D and E, but these values seem very low, around 20 PFU per lung at 5 DPI while the mice were inoculated with 5*10^4 PFU. I assume a multiplication of your values was forgotten somewhere in the analysis of the plaque assay.

Thank you to the reviewer for highlighting this error. The viable virus we recovered was from bronchioalveolar lavage and nasal lavage rather than assessing whole tissue. We assayed 200uL of lavage fluid and missed a multiplication factor of 5 to convert the plaque assay results to PFU/mL nasal lavage or PFU/mL BAL. This has now been rectified in Figure EV2.

Referee #2 (Comments on Novelty/Model System for Author):
Authors have suitably addressed all reviewer suggestions/comments.
Many thanks to the reviewer.

17th Oct 2023

Dear Dr. Edgar,

We are pleased to inform you that your manuscript is accepted for publication and is now being sent to our publisher to be included in the next available issue of EMBO Molecular Medicine.

Please read below for additional IMPORTANT information regarding your article, its publication and the production process.

Congratulations on your interesting work,

Zeljko Durdevic

Zeljko Durdevic
Editor
EMBO Molecular Medicine

Follow us on Twitter @EmboMolMed
Sign up for eTOCs at embopress.org/alertsfeeds

*** *** *** IMPORTANT INFORMATION *** *** ***

SPEED OF PUBLICATION
The journal aims for rapid publication of papers, using using the advance online publication "Early View" to expedite the process: A properly copy-edited and formatted version will be published as "Early View" after the proofs have been corrected. Please help the Editors and publisher avoid delays by providing e-mail address(es), telephone and fax numbers at which author(s) can be contacted.

Should you be planning a Press Release on your article, please get in contact with embomolmed@wiley.com as early as possible, in order to coordinate publication and release dates.

LICENSE AND PAYMENT:

All articles published in EMBO Molecular Medicine are fully open access: immediately and freely available to read, download and share.

EMBO Molecular Medicine charges an article processing charge (APC) to cover the publication costs. You, as the corresponding author for this manuscript, should have already received a quote with the article processing fee separately. Please let us know in case this quote has not been received.

Once your article is at Wiley for editorial production you will receive an email from Wiley's Author Services system, which will ask you to log in and will present you with the publication license form for completion. Within the same system the publication fee can be paid by credit card, an invoice, pro forma invoice or purchase order can be requested.

Payment of the publication charge and the signed Open Access Agreement form must be received before the article can be published online.

PROOFS

You will receive the proofs by e-mail approximately 2 weeks after all relevant files have been sent o our Production Office. Please return them within 48 hours and if there should be any problems, please contact the production office at embopressproduction@wiley.com.

Please inform us if there is likely to be any difficulty in reaching you at the above address at that time. Failure to meet our

deadlines may result in a delay of publication.

All further communications concerning your paper proofs should quote reference number EMM-2023-17932-V3 and be directed to the production office at embopressproduction@wiley.com.

Thank you,

Zeljko Durdevic
Editor
EMBO Molecular Medicine

EMBO Press Author Checklist

Corresponding Author Name: Siu Kwan Sze	
Journal Submitted to: EMBO Molecular Medicine	
Manuscript Number: EMM-2023-18526	

USEFUL LINKS FOR COMPLETING THIS FORM
[The EMBO Journal - Author Guidelines]
[EMBO Reports - Author Guidelines]
[Molecular Systems Biology - Author Guidelines]
[EMBO Molecular Medicine - Author Guidelines]

Reporting Checklist for Life Science Articles (updated January
This checklist is adapted from Materials Design Analysis Reporting (MDAR) Checklist for Authors. MDAR establishes a minimum set of requirements in transparent reporting in the life sciences (see Statement of Task: 10.31222/osf.io/9sm4x). Please follow the journal's guidelines in preparing your
Please note that a copy of this checklist will be published alongside your article.

Abridged guidelines for figures
1. Data
The data shown in figures should satisfy the following conditions:
- the data were obtained and processed according to the field's best practice and are presented to reflect the results of the experiments in an accurate and unbiased manner.
- ideally, figure panels should include only measurements that are directly comparable to each other and obtained with the same assay.
- plots include clearly labeled error bars for independent experiments and sample sizes. Unless justified, error bars should not be shown for technical
- if n<5, the individual data points from each experiment should be plotted. Any statistical test employed should be justified.
- Source Data should be included to report the data underlying figures according to the guidelines set out in the authorship guidelines on Data

2. Captions
Each figure caption should contain the following information, for each panel where they are relevant:
- a specification of the experimental system investigated (eg cell line, species name).
- the assay(s) and method(s) used to carry out the reported observations and measurements.
- an explicit mention of the biological and chemical entity(ies) that are being measured.
- an explicit mention of the biological and chemical entity(ies) that are altered/varied/perturbed in a controlled manner.
- the exact sample size (n) for each experimental group/condition, given as a number, not a range;
- a description of the sample collection allowing the reader to understand whether the samples represent technical or biological replicates (including how many animals, litters, cultures, etc.).
- a statement of how many times the experiment shown was independently replicated in the laboratory.
- definitions of statistical methods and measures:
  - common tests, such as t-test (please specify whether paired vs. unpaired), simple χ2 tests, Wilcoxon and Mann-Whitney tests, can be unambiguously identified by name only, but more complex techniques should be described in the methods section;
  - are tests one-sided or two-sided?
  - are there adjustments for multiple comparisons?
  - exact statistical test results, e.g., P values = x but not P values < x;
  - definition of 'center values' as median or average;
  - definition of error bars as s.d. or s.e.m.

Please complete ALL of the questions below.
Select "Not Applicable" only when the requested information is not relevant for your study.

Materials

Newly Created Materials	Information included in the manuscript?	In which section is the information available? (Reagents and Tools Table, Materials and Methods, Figures, Data Availability Section)
New materials and reagents need to be available; do any restrictions apply?	Not Applicable	

Antibodies	Information included in the manuscript?	In which section is the information available? (Reagents and Tools Table, Materials and Methods, Figures, Data Availability Section)
For **antibodies** provide the following information:<br>- Commercial antibodies: RRID (if possible) or supplier name, catalogue number and or/clone number<br>- Non-commercial: RRID or citation	Yes	Materials and Methods

DNA and RNA sequences	Information included in the manuscript?	In which section is the information available? (Reagents and Tools Table, Materials and Methods, Figures, Data Availability Section)
**Short novel DNA or RNA including primers, probes**: provide the sequences.	Not Applicable	

Cell materials	Information included in the manuscript?	In which section is the information available? (Reagents and Tools Table, Materials and Methods, Figures, Data Availability Section)
**Cell lines:** Provide species information, strain. Provide accession number in repository **OR** supplier name, catalog number, clone number, and/**OR** RRID.	Yes	Materials and Methods
**Primary cultures:** Provide species, strain, sex of origin, genetic modification status.	Not Applicable	
Report if the cell lines were recently **authenticated** (e.g., by STR profiling) and tested for mycoplasma contamination.	Not Applicable	

Experimental animals	Information included in the manuscript?	In which section is the information available? (Reagents and Tools Table, Materials and Methods, Figures, Data Availability Section)
**Laboratory animals or Model organisms:** Provide species, strain, sex, age, genetic modification status. Provide accession number in repository **OR** supplier name, catalog number, clone number, **OR** RRID.	Yes	Materials and Methods Jackson Lab (B6;129S4-Pcmt1tm1Scl/J) Strain #:023343 RRID:IMSR_JAX:023343Info
**Animal observed in or captured from the field:** Provide species, sex, and age where possible**.**	Not Applicable	
Please detail **housing and husbandry conditions**.	Yes	Materials and Methods

Plants and microbes	Information included in the manuscript?	In which section is the information available? (Reagents and Tools Table, Materials and Methods, Figures, Data Availability Section)
**Plants:** provide species and strain, ecotype and cultivar where relevant, unique accession number if available, and source (including location for collected wild specimens).	Not Applicable	
**Microbes:** provide species and strain, unique accession number if available, and source.	Not Applicable	

Human research participants	Information included in the manuscript?	In which section is the information available? (Reagents and Tools Table, Materials and Methods, Figures, Data Availability Section)
If collected and within the bounds of privacy constraints report on age, sex and gender or ethnicity for all study participants.	Not Applicable	

Core facilities	Information included in the manuscript?	In which section is the information available? (Reagents and Tools Table, Materials and Methods, Figures, Data Availability Section)
If your work benefited from core facilities, was their service mentioned in the acknowledgments section?	Not Applicable	

Design

Study protocol	Information included in the manuscript?	In which section is the information available? (Reagents and Tools Table, Materials and Methods, Figures, Data Availability Section)

If study protocol has been **pre-registered, provide DOI in the manuscript**. For clinical trials, provide the trial registration number **OR** cite DOI.	Not Applicable	
Report the **clinical trial registration number** (at ClinicalTrials.gov or equivalent), where applicable.	Not Applicable	

Laboratory protocol	Information included in the manuscript?	In which section is the information available? (Reagents and Tools Table, Materials and Methods, Figures, Data Availability Section)
Provide DOI OR other citation details if **external detailed step-by-step protocols** are available.	Not Applicable	

Experimental study design and statistics	Information included in the manuscript?	In which section is the information available? (Reagents and Tools Table, Materials and Methods, Figures, Data Availability Section)
Include a statement about **sample size** estimate even if no statistical methods were used.	Yes	Materials and Methods, Figures
Were any steps taken to minimize the effects of subjective bias when allocating animals/samples to treatment (e.g. **randomization procedure**)? If yes, have they been described?	Not Applicable	
Include a statement about **blinding** even if no blinding was done.	Not Applicable	
Describe **inclusion/exclusion criteria** if samples or animals were excluded from the analysis. Were the criteria pre-established?<br><br>If sample or data points were omitted from analysis, report if this was due to attrition or intentional exclusion and provide justification.	Not Applicable	
For every figure, are **statistical tests** justified as appropriate? Do the data meet the assumptions of the tests (e.g., normal distribution)? Describe any methods used to assess it. Is there an estimate of variation within each group of data? Is the variance similar between the groups that are being statistically compared?	Yes	Materials and Methods, Figures

Sample definition and in-laboratory replication	Information included in the manuscript?	In which section is the information available? (Reagents and Tools Table, Materials and Methods, Figures, Data Availability Section)
In the figure legends: state number of times the experiment was **replicated** in laboratory.	Yes	Materials and Methods, Figures
In the figure legends: define whether data describe **technical or biological replicates**.	Yes	Materials and Methods, Figures

Ethics

Ethics	Information included in the manuscript?	In which section is the information available? (Reagents and Tools Table, Materials and Methods, Figures, Data Availability Section)
Studies involving **human participants**: State details of **authority granting ethics approval** (IRB or equivalent committee(s), provide reference number for approval.	Not Applicable	
Studies involving **human participants**: Include a statement confirming that **informed consent** was obtained from all subjects and that the experiments conformed to the principles set out in the WMA Declaration of Helsinki and the Department of Health and Human Services Belmont Report.	Not Applicable	
Studies involving **human participants:** For publication of **patient photos**, include a statement confirming that consent to publish was obtained.	Not Applicable	
Studies involving experimental **animals**: State details of **authority granting ethics approval** (IRB or equivalent committee(s), provide reference number for approval. Include a statement of compliance with ethical regulations.	Yes	Materials and Methods
Studies involving **specimen and field samples:** State if relevant **permits** obtained, provide details of authority approving study; if none were required, explain why.	Not Applicable	

Dual Use Research of Concern (DURC)	Information included in the manuscript?	In which section is the information available? (Reagents and Tools Table, Materials and Methods, Figures, Data Availability Section)
Could your study fall under dual use research restrictions? Please check biosecurity documents and list of **select agents and toxins** (CDC): https://www.selectagents.gov/sat/list.htm	Not Applicable	
If you used a select agent, is the security level of the lab appropriate and reported in the manuscript?	Not Applicable	
If a study is subject to dual use research of concern regulations, is the name of the **authority granting approval and reference number** for the regulatory approval provided in the manuscript?	Not Applicable	

Reporting

The MDAR framework recommends adoption of discipline-specific guidelines, established and endorsed through community initiatives. Journals have their own policy about requiring specific guidelines and recommendations to complement MDAR.

Adherence to community standards	Information included in the manuscript?	In which section is the information available? (Reagents and Tools Table, Materials and Methods, Figures, Data Availability Section)
State if relevant guidelines or checklists (e.g., **ICMJE, MIBBI, ARRIVE, PRISMA**) have been followed or provided.	Not Applicable	
For **tumor marker prognostic studies,** we recommend that you follow the **REMARK** reporting guidelines (see link list at top right). See author guidelines, under 'Reporting Guidelines'. Please confirm you have followed these guidelines.	Not Applicable	
For **phase II and III randomized controlled trials**, please refer to the **CONSORT** flow diagram (see link list at top right) and submit the CONSORT checklist (see link list at top right) with your submission. See author guidelines, under 'Reporting Guidelines'. Please confirm you have submitted this list.	Not Applicable	

Data Availability

Data availability	Information included in the manuscript?	In which section is the information available? (Reagents and Tools Table, Materials and Methods, Figures, Data Availability Section)
Have **primary datasets** been deposited according to the journal's guidelines (see 'Data Deposition' section) and the respective accession numbers provided in the Data Availability Section?	Not Applicable	
Were **human clinical and genomic datasets** deposited in a public access-controlled repository in accordance to ethical obligations to the patients and to the applicable consent agreement?	Not Applicable	
Are **computational models** that are central and integral to a study available without restrictions in a machine-readable form? Were the relevant accession numbers or links provided?	Not Applicable	
If publicly available data were reused, provide the respective **data citations in the reference list**.	Not Applicable	
